# Synovial fibroblast derived small extracellular vesicles miRNA15-29148 promotes articular chondrocyte apoptosis in rheumatoid arthritis

**DOI:** 10.1038/s41413-025-00430-3

**Published:** 2025-06-12

**Authors:** Zhenyu Zhang, Lulu Liu, Huibo Ti, Minnan Chen, Yuechun Chen, Deyan Du, Wenjing Zhan, Tongtong Wang, Xian Wu, Junjie Wu, Dong Mao, Zhengdong Yuan, Jingjing Ruan, Genxiang Rong, Feng-lai Yuan

**Affiliations:** 1 Institute of Integrated Chinese and Western Medicine, Affiliated Hospital of Jiangnan University, Jiangsu, China; 2 Biomedical engineering facility of National Infrastructures for Translational Medicine, State Key Laboratory of Complex Severe and Rare Diseases in Peking Union Medical College Hospital, Chinese Academy of Medical Science and Peking Union Medical College, Beijing, China; 3 Nantong First People’s Hospital, Nantong, China; 4 The Key Laboratory of Synthetic and Biological Colloids, Ministry of Education, School of Chemical and Material Engineering, Jiangnan University, Wuxi, China; 5 The Key Laboratory of Anti-Inflammatory and Immune Medicine, Ministry of Education, Anhui Medical University, Hefei, China; 6 Orthopaedic Institute, Wuxi 9th People’s Hospital Affiliated to Soochow University, Wuxi, China; 7 Department of Respiratory and Critical Care Medicine, The First Affiliated Hospital of Anhui Medical University, Hefei, China; 8 Department of Orthopedics, The First Affiliated Hospital of Anhui Medical University, Hefei, China

**Keywords:** Pathogenesis, Bone

## Abstract

Rheumatoid arthritis (RA) is a systemic autoimmune disease in which synovial fibroblasts (SFs) maintain chronic inflammation by secreting proinflammatory mediators, leading to joint destruction. While the role of proinflammatory mediators in this process is well-established, the contribution of non-inflammatory regulators in SFs to joint pathology remains poorly understood. In this study, we investigated the non-inflammatory role of SFs in RA using a co-culture model, and found that SFs from RA patients promote apoptosis of human chondrocytes. Mechanistic investigations reveal that SFs can secrete small extracellular vesicles (sEVs), which are taken up by chondrocytes and induce chondrocyte apoptosis in both normal chondrocytes and chondrocytes from patients with RA. sEV-derived miRNA 15-29148 are identified as key signaling molecules mediating the apoptosis effects of chondrocytes. Further studies reveal that SF-derived miRNA 15-29148 targeting CIAPIN1 results in increased chondrocyte apoptosis. We further demonstrate that SF-derived miRNA 15-29148 is transferred to chondrocytes, exacerbating cartilage damage in vivo. Moreover, chondrocyte-specific aptamer-modified polyamidoamine nanoparticles not only ameliorated RA but also prevented its onset. This study suggests that, in RA, the secretion of specific sEV-miRNAs from SFs plays a crucial role in promoting chondrocyte apoptosis, potentially through non-inflammatory regulation, and that sEV-miRNA inhibition in SFs may represent an early preventive treatment strategy for cartilage degradation in RA.

## Introduction

Rheumatoid arthritis (RA) is a chronic autoimmune disorder characterized by the persistent inflammation of the synovial joints, leading to the progressive destruction of cartilage and bone, engendering severe morbidity and disability.^1,2^ The role of inflammatory mediators in this destructive process in RA pathophysiology has been examined extensively,^3,4^ and significant advancements in the treatment of RA have been achieved through targeting inflammatory mediators. However, once structural damage to the cartilage and bone has occurred, it becomes irreversible; thus, the effective inhibition of joint destruction remains one of the most important goals of RA therapy.^5,6^ Aside from the well-documented proinflammatory regulatory mechanism of synovial fibroblasts (SFs) in cartilage destruction, accumulating evidence has indicated that SFs non-inflammatory mediators also contribute to cartilage damage, either by direct invasion or indirectly via proteases and adhesion-facilitating factors.^7–10^ However, it remains unclear whether other non-inflammatory regulatory factors from SFs cause cartilage damage in RA.

Chondrocytes are chiefly responsible for maintaining cartilage homeostasis and contribute to the pathogenesis of RA.^11,12^ In the pathological state of RA, the secretion capacity of chondrocytes is reduced, as reflected in extracellular matrix (ECM) components such as collagen, glycoproteins, proteoglycans, and hyaluronic acid.^13,14^ Apoptotic chondrocytes are found increasingly frequently in articular cartilage samples, resulting in RA.^15,16^ We previously demonstrated that an acidic microenvironment in the joints induces an increase in calcium ions in chondrocytes, which promotes chondrocyte apoptosis and is an important cause of cartilage damage in RA.^17,18^ Other groups have also reported that the inhibition of apoptotic changes in chondrocytes reduced articular damage in animal models of RA and displayed clinical efficacy.^19,20^ Thus, advances in this field could be crucial for revealing the mechanism of chondrocyte apoptosis in the RA milieu. However, how articular chondrocytes (ACs) are regulated by SFs, especially through non-inflammatory mechanisms, is not yet known.

Small extracellular vesicles (sEVs) are cellularly secreted extracellular vesicles with diameters ranging from 30 to 200 nm, which have emerged as significant regulators of intercellular signaling and promising biomarkers.^21,22^ sEVs transport intricate biological molecules, encompassing proteins, lipids, DNA, and RNA. Among these substances, microRNAs (miRNAs) are considered one of the most important signaling molecules because they can be secreted into extracellular fluid and transported to target cells through vesicles.^23,24^ The specific substances carried by sEVs can profoundly influence the fate of recipient cells, highlighting their potential as targeted therapeutic agents.^25,26^ Functional studies have demonstrated that sEV-exerted miRNA delivery acts as an intercellular communication mediator, participating in RA pathophysiology.^24^ However, it remains unknown whether sEVs can mediate crosstalk between SFs and fibroblasts.

In this study, we observed that SFs derived from patients with RA induce chondrocyte apoptosis, while acknowledging the importance of considering the overall inflammatory burden in the disease model. Detailed mechanism investigations revealed that RASF-derived sEV-miRNA 15-29148 induced chondrocyte apoptosis. We then investigated increased RASF-derived sEV-miRNA 15-29148 levels, observing associations with both elevated synovia miRNA 15-29148 levels and increased cartilage damage. We present in vitro and in vivo evidence to demonstrate that RASF-derived sEV-miRNA 15-29148 can transfer to chondrocytes to promote chondrocyte apoptosis. Moreover, we show that chondrocyte-targeted antago miRNA 15-29148 treatment could prevent cartilage damage in collagen-induced arthritis (CIA) mice.

## Results

### Apoptosis of human ACs under co‑culture conditions between ACs and RASFs derived from RA human joint specimens

To enhance the clinical relevance of our study, all in vitro experiments were conducted using human primary cells. Specifically, we isolated human primary synovial fibroblasts (Fig. S1a) and human primary chondrocytes, followed by cell characterization (Fig. S1b, c).

**Fig. 1:**
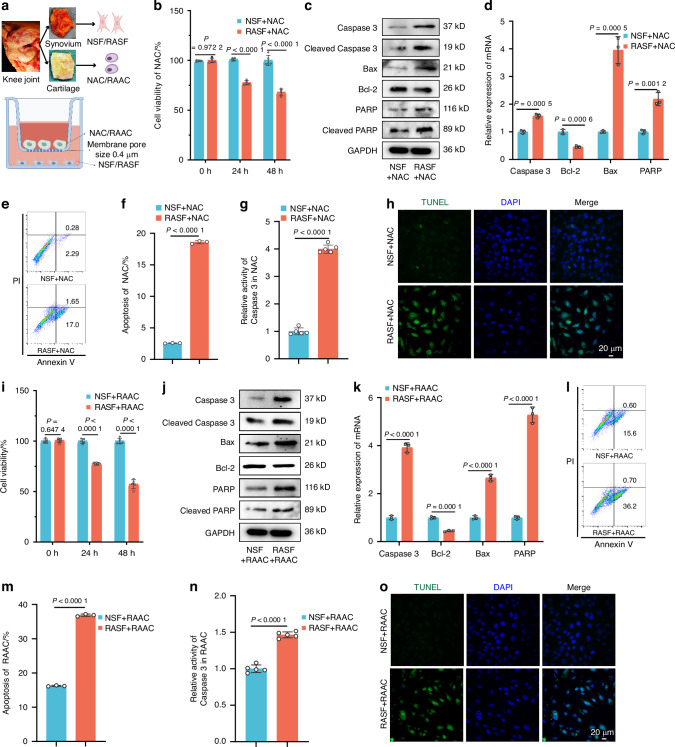
Synovial Fibroblasts from RA Patients Promote AC Apoptosis. **a** Strategy for co-culture of human primary synovial fibroblasts and human primary chondrocytes. **b**, **i** Cell viability of NAC and RAAC co-cultured with NSF or RASF was analyzed using CCK8 over different time periods (*n* = 5). **c**, **j** Western blot analysis detected the expression levels of apoptosis-related proteins in NAC and RAAC after 48 h of co-culture with NSF/RASF (*n* = 3). **d**, **k** RT-qPCR was used to determine the expression levels of apoptosis-related genes in NAC and RAAC (*n* = 3). **e**, **f**, **l**, **m** Apoptosis analysis of NAC and RAAC was performed using flow cytometry with Annexin V-FITC/PI staining (*n* = 5). **g**, **n** Caspase-3 activity in NAC and RAAC co-cultured with NSF or RASF for 48 h was measured (*n* = 5). **h**, **o** TUNEL staining of NAC and RAAC co-cultured with NSF or RASF for 48 h was performed. (**b**, **d**, **f**, **g**, **i**, **k**–**n**) Data are presented as mean ± SD. Student’s t-test was used to calculate *P*-values between NSF and RASF

To investigate the regulatory effect of synovial fibroblasts on chondrocytes, we non-contact co-cultured normal synovial fibroblasts (NSFs) or synovial fibroblasts from patients with RA (RASFs) with normal primary articular chondrocytes (NACs) or primary articular chondrocytes from patients with RA (RAACs), respectively (Fig. 1a). We found that the viability of NACs was significantly reduced in co-culture with RASFs (compared to NSF co-culture), with RASFs decreasing NAC cell viability by 30% after 48 h of co-culture (compared to that at 24 h) (Fig. 1b). This indicates that RASFs can remotely and persistently reduce NAC viability within 48 h. To confirm cross-contamination in our co-culture system, we stained recipient cells with ACs (Collagen II) and SFs (Podoplanin) markers. Collagen II expression was reduced in chondrocytes co-cultured with RASF, suggesting an effect on chondrocyte function. Podoplanin was absent in both groups, indicating no SFs contamination. Our co-culture design effectively avoided cross-contamination, ensuring experimental accuracy (Fig. S2). Given that chondrocyte apoptosis is the most studied and common mode of cell death, we assessed apoptosis levels in chondrocytes co-cultured with synovial fibroblasts and found significant alterations in both apoptosis-related proteins (Fig. 1c) and genes (Fig. 1d) in NACs co-cultured with RASFs. Additionally, flow cytometry showed that co-culture with RASFs resulted in a 7.26-fold increase in NAC apoptosis (from 2.57% to 18.65%) compared to NSF co-culture (Fig. 1e, f). Caspase 3, a key protease in the apoptosis pathway, exhibited a fourfold increase in activity in NACs co-cultured with RASFs (Fig. 1g). TUNEL staining further indicated that RASFs promoted NAC apoptosis (Fig. 1h). These findings suggest that RASFs can induce apoptosis in NACs. To determine whether RASFs can also promote apoptosis in RAACs, we simultaneously investigated the apoptosis levels in RAACs co-cultured with NSFs or RASFs. The results showed that RASFs could also promote the apoptosis of RAACs (Fig. 1i–o).

### RASF-derived sEVs induce AC apoptosis

**Fig. 2:**
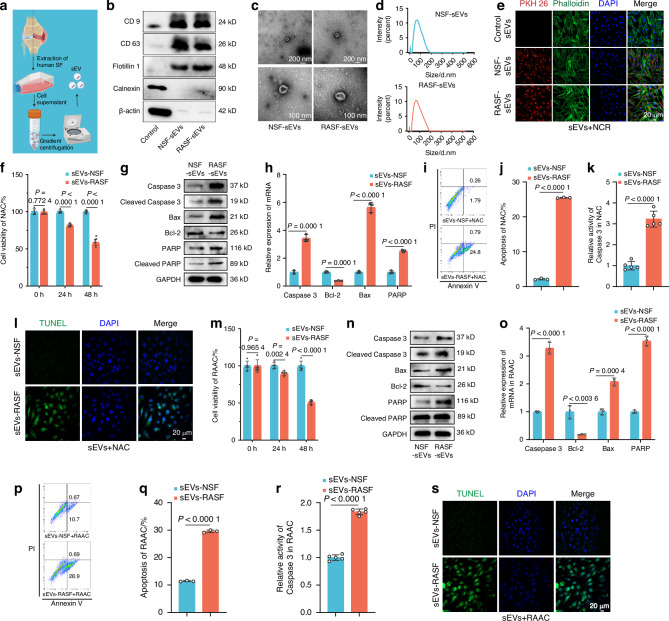
RASF Promotes Apoptosis of AC by Secreting sEVs. **a** Flowchart depicting the sEVs purification procedure via differential ultracentrifugation. **b** Western blot analysis of CD9, CD63, Flotillin 1, Calnexin, and β-actin expression levels in NSF/RASF sEVs (*n* = 3). **c** Transmission electron microscopy (TEM) images of sEVs purified from NSF or RASF. **d** Nanoparticle tracking analysis of the size distribution of NSF/RASF sEVs. **e** NSF/RASF sEVs stained with PKH26 red membrane dye. **f**, **m** CCK8 assay of cell viability of NAC and RAAC treated with NSF/RASF sEVs over different time periods (*n* = 5). **g**, **n** Western blot analysis detected the expression levels of apoptosis-related proteins in NAC and RAAC treated with NSF/RASF sEV for 48 h (*n* = 3). **h**, **o** The expression levels of apoptosis-related genes in NAC and RAAC (*n* = 3). **i**, **j**, **p**, **q** Apoptosis analysis of NAC and RAAC using flow cytometry with Annexin V-FITC/PI staining (*n* = 3). **k**, **r** Caspase-3 activity in NAC and RAAC after 48 h of culture with NSF or RASF sEVs (*n* = 5). **l**, **s** TUNEL staining of NAC and RAAC cultured with NSF or RASF sEVs for 48 h. (**f**, **h**, **j**, **k**, **m**, **o**, **q**, **r**) Data are presented as mean ± SD. Student’s *t*-test was used to calculate p-values between NSF and RASF sEV treatments

To determine whether RASFs regulate chondrocyte apoptosis through sEV secretion, we isolated and purified sEVs from the culture supernatants of NSFs and RASFs after 48 h using differential ultracentrifugation (Figs. 2a, S3a). Western blotting confirmed the expression of sEV markers CD9, CD63, and Flotillin 1, while negative results were observed for the expression of Calnexin and β-actin (Fig. 2b). Transmission electron microscopy (TEM) revealed characteristic cup-shaped vesicles with an average diameter of 110 nm (Figs. 2c, S3b). Nanoparticle tracking analysis (NTA) revealed particle sizes ranging from 30 to 200 nm, consistent with expected sEV dimensions (Figs. 2d, S3c). These findings confirmed the successful isolation and high purity of the sEVs.

To investigate whether sEVs secreted by RASFs or NSFs can be recognized, captured, and internalized by chondrocytes, we labeled sEVs with PKH26 and co-incubated them with chondrocytes for 12 h. Laser confocal microscopy revealed the predominant capture and internalization of the sEVs by the chondrocytes (Figs. 2e, S3d). To explore the effects of RASF-sEVs on the expression of aggrecan in NAC, we detected the expression level of aggrecan in NAC after RASF-sEVs was administered. Results showed that RASF-sEVs down-regulated aggrecan mRNA and protein levels in NAC (Fig. S4a–c).

To investigate whether sEVs promote chondrocyte apoptosis, we co-incubated sEVs secreted by NSFs or RASFs with NACs or RAACs for 48 h. A CCK8 assay revealed that sEVs secreted by RASFs significantly reduced the viability of NACs and RAACs (compared to those secreted by NSFs) (Fig. 2f, m). Western blotting and RT-qPCR revealed that sEVs from RASFs significantly upregulated pro-apoptotic proteins and genes while downregulating anti-apoptotic proteins and genes (Fig. 2g, h, n, o). Flow cytometry revealed that RASF-secreted sEVs increased the percentage of apoptosis from 2.05% to 25.59% in NACs (Fig. 2i, j), and from 10.74% to 29.59% in RAACs (Fig. 2p, q). Additionally, compared to NSF-derived sEVs, RASF-derived sEVs increased caspase 3 protease activity by 3.2-fold in NACs (Fig. 2k) and by 1.8-fold in RAACs (Fig. 2r). TUNEL staining further confirmed that RASF-secreted sEVs significantly promoted apoptosis in both NACs and RAACs (Fig. 2l, s).

To investigate whether RASF-sEVs exhibit similar effects in vivo as compared to those from NSF-sEVs, we administered NSF-sEVs or RASF-sEVs to mice *via* intra-articular injection. The results demonstrated that, in contrast to NSF-sEVs, administration of RASF-sEVs to mice promoted the apoptosis of ACs and exacerbated the arthritic manifestations in CIA mice (Fig. S5a–m). These findings suggest that RASF-sEVs can also aggravate the progression of RA by inducing AC apoptosis in vivo.

Collectively, these findings suggest that sEV secreted by RASFs or NSFs can be captured and internalized by chondrocytes and that RASF-sEVs significantly promote apoptosis in NACs and enhance apoptosis in RAACs.

### miRNA 15-29148 is enriched in RASF-derived sEVs and responsible for the RASF-derived sEV-induced apoptosis of ACs

**Fig. 3:**
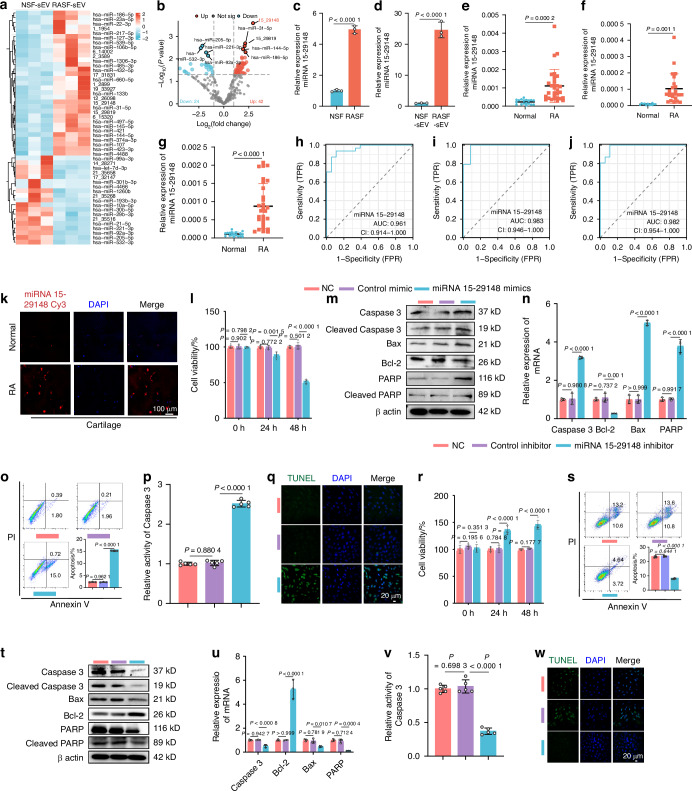
miRNA 15-29148 in SEV Secreted by RASF Promotes Apoptosis of AC. **a** Heat map depicting the top 50 differentially expressed miRNAs in RASF sEV. **b** Volcano plot of miRNA expression level differences between RA patients and controls in sEV. **c**, **d** miRNA 15-29148 expression levels in human primary synovial fibroblasts and their secreted sEVs. **e**–**g** miRNA 15-29148 expression levels in synovial tissues synovial fluid cartilage of RA patients or controls. (**c**–**g)** All bar graphs are presented as the mean ± SD. by Student’s *t*-test. **h**–**j** Sensitivity and specificity of the ROC curve in assessing the prediction of RA by miRNA 15-29148 expression level in synovial tissue, joint fluid and cartilage tissue. **k** FISH analysis of RA cartilage tissues levels of miRNA 15-29148. **l**, **r** CCK8 analysis of NAC and RAAC cell viability (*n* = 5). **m**, **t** Western blot analysis detected the expression levels of apoptosis-related proteins in NCR and RAAC (*n* = 3). **n**, **u** The expression levels of apoptosis-related genes in NAC and RAAC (*n* = 3). **o**, **s** Apoptosis analysis of NAC and RAAC using flow cytometry with Annexin V-FITC/PI staining (*n* = 3). **p**, **v** Caspase-3 activity in NAC and RAAC (*n* = 5). **q**, **w** TUNEL staining of NAC and RAAC. (**l**, **n**–**p**, **r**, **s**, **u**, **v**) Data are presented as mean ± SD. One-way ANOVA was used to calculate *P*-values

To systematically identify the key miRNAs within RASF-sEVs, we conducted ultra-trace miRNA profiling using microarrays on sEVs secreted by NSFs and RASFs (Fig. S6a–d). Sequencing revealed 42 upregulated and 24 downregulated miRNAs in RASF-sEVs compared to those in NSF-sEVs (Figs. 3a, b, S6e). The top 10 miRNAs with the highest differential expression were miRNA 15-29148, hsa-miR-31-5p, miRNA 15-29819, hsa-miR-186-5p, hsa-miR-145-5p, hsa-miR-144-5p, hsa-miR-205-5p, hsa-miR-532-3p, hsa-miR-221-3p, and hsa-miR-92a-3p (Fig. S6h, i). These findings were validated using human primary synoviocytes and their sEVs, confirming the dysregulation of all 10 miRNAs by RT-qPCR (Figs. 3c, d; S7a–r).

Subsequent functional clustering and literature review indicated that miRNA 15-29148, miRNA 15-29819, hsa-miR-144-5p, and hsa-miR-221-3p might be associated with apoptosis (Fig. S6f, g, j, k). Mimics and inhibitors were constructed for these four miRNAs and transfected into primary chondrocytes; successful transfection was confirmed by RT-qPCR and fluorescence validation (Fig. S8a–q). Further investigation revealed that only miRNA 15-29148 mimics promoted chondrocyte apoptosis, leading us to focus on miRNA 15-29148 for subsequent study(Fig. S9a–j). Basic information of miRNA 15-29148 was shown in the Fig. S10a, b.

Given the potential significance of miRNA 15-29148 in RA and chondrocyte apoptosis, we explored it in detail. Initially, we examined an independent cohort comprising 18 controls and 30 patients with RA to assess miRNA 15-29148 expression levels in synovial tissue, sEVs within joint fluid, and cartilage tissue. RT-qPCR analysis revealed a 4.844-fold upregulation of miRNA 15-29148 in synovial tissues, a 12.436-fold increase in the levels of sEVs from joint fluid, and an 8.162-fold elevation in cartilage tissues of patients with RA compared to controls (Fig. 3e–g).

To investigate the diagnostic potential of miRNA 15-29148 for RA, we performed ROC curve analysis on its expression levels in synovial tissues, sEVs from joint fluids, and cartilage tissues. We found that the miRNA 15-29148 levels in these tissues could serve as diagnostic markers for RA, with AUC values exceeding 0.95 (Fig. 3h–j). Fluorescence in situ hybridization (FISH) further confirmed the significant upregulation of miRNA 15-29148 in both the synovial and cartilage tissues of patients with RA compared to that in controls (Figs. 3k, S7s). To evaluate the inflammatory effects of miRNA 15-29148 in vitro, we found that its direct impact is insignificant (Fig. S11a–f).

To assess the regulatory role of miRNA 15-29148 in chondrocyte apoptosis, we examined the apoptosis levels in chondrocytes following transfection with miRNA 15-29148 mimics or inhibitors in both NACs and RAACs. CCK8 assays demonstrated that after 48 h of incubation, miRNA 15-29148 mimics decreased NAC cell viability by 51.49% (Fig. 3l) and RAAC cell viability by 67.39% (Fig. S12a) compared to controls, while miRNA 15-29148 inhibitors increased NAC cell viability by 43.94% (Fig. S13a) and RAAC cell viability by 45.46% (Fig. 3r) compared to controls. Western blotting and RT-qPCR revealed that miRNA 15-29148 mimics elevated the expression of pro-apoptotic proteins and genes while reducing that of anti-apoptotic proteins and genes in both NACs and RAACs after 48 h of incubation (Figs. 3m, n, S12b, c). Similarly, miRNA 15-29148 inhibitors increased the expression of pro-apoptotic proteins and genes and decreased that of anti-apoptotic proteins and genes in both NACs and RAACs (Figs. 3t, u, S13b, c). Flow cytometry results showed that miRNA 15-29148 mimics increased the proportion of apoptotic NACs from 2.19% to 15.72% (Fig. 3o) and RAACs from 17.11% to 33.07% (Fig. S12d, e) compared to controls, whereas miRNA 15-29148 inhibitors reduced the proportion of apoptotic RAACs from 23.80% to 8.36% compared to controls (Fig. 3s). Additionally, miRNA 15-29148 mimics increased caspase 3 protease activity in NACs and RAACs by 2.52-fold and 4.01-fold, respectively (Figs. 3p, S12f), while miRNA 15-29148 inhibitors decreased caspase 3 protease activity in NACs and RAACs by 2.79-fold and 2.70-fold, respectively (Figs. 3v, S13d). TUNEL staining further confirmed that miRNA 15-29148 mimics promoted apoptosis in both NACs and RAACs (Figs. 3q, S12g), whereas miRNA 15-29148 inhibitors inhibited apoptosis in both cell types (Figs. 3w, S13e). To explore miRNA 15-29148 effects on AC aggrecan, we measured aggrecan levels after miRNA 15-29148 transfection. Both down-regulated aggrecan mRNA and protein (Fig. S4d–f). Finally, we studied the impact of miRNA 15-29148 on NAC pyroptosis by assessing NLRP3, caspase-1, and c-caspase-1 levels in NAC. Results showed no significant changes in chondrocyte volume, NLRP3, caspase-1, or c-caspase-1 expression. Thus, miRNA 15-29148 likely does not affect NACs *via* pyroptosis (Fig. S14a–f, S15a–f). Overall, these findings indicate that RASFs can deliver highly expressed miRNA 15-29148 into human primary chondrocytes *via* sEV secretion, promoting apoptosis.

### miRNA 15-29148 exacerbates AC apoptosis by downregulating CIAPIN1 expression in ACs

**Fig. 4:**
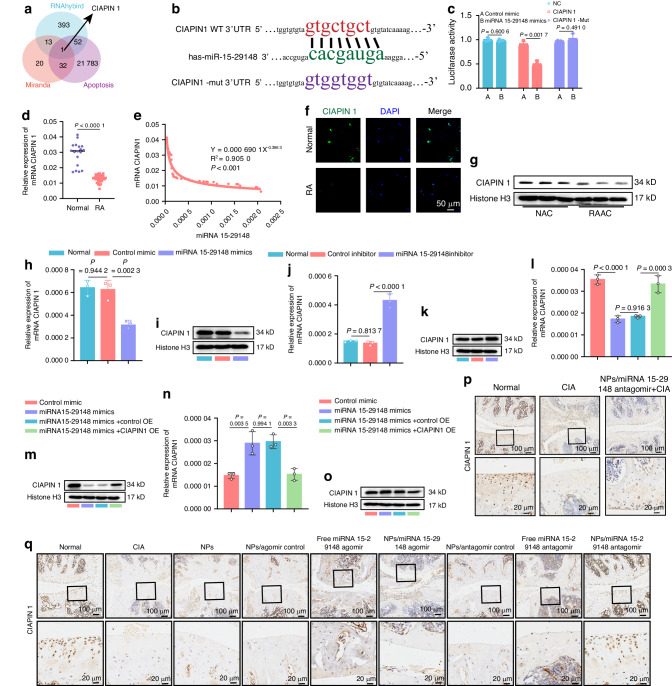
CIAPIN 1, the Target of miRNA 15-29148, is Downregulated in RA. **a** Target genes of miRNA 15-29148 predicted using miRanda, RNAhybrid, and apoptosis databases. **b** Schematic representation of putative miRNA 15-29148 binding sites on the 3’ UTR of CIAPIN 1 in humans. **c** Human primary chondrocytes transfected with wild-type or mutated CIAPIN 1 3’ UTR luciferase constructs and control mimic or miRNA 15-29148 mimics. **d** mRNA CIAPIN 1 expression levels downregulated in RA patients (*n* = 30) compared with controls (*n* = 18). Data are presented as mean value ± SD (*n* = 3). Student’s *t*-test was used to calculate *P* values between Normal and RA groups. **e** Spearman’s correlation coefficient analysis between miRNA 15-29148 levels in cartilage and CIAPIN 1 mRNA levels in cartilage from patients. **f** Immunofluorescence staining of CIAPIN 1 in cartilage tissue. Nucleus, blue (DAPI); CIAPIN 1, green. Scale: 50 μm. Representative images of three biologically independent samples in each group. **g** Western blot analysis of CIAPIN 1 expression levels in NAC and RAAC (*n* = 3). **h**, **i** Gene and protein levels of CIAPIN 1 after transfection with miRNA 15-29148 mimics. **j**, **k** Gene and protein levels of CIAPIN 1 after transfection with miRNA 15-29148 inhibitor. **l**, **m** Gene and protein levels of CIAPIN 1 after transfection with miRNA 15-29148 mimics and CIAPIN 1 overexpression constructs. **n**, **o** Gene and protein levels of CIAPIN 1 after transfection with miRNA 15-29148 inhibitor and SI-CIAPIN 1 inhibitor. (*n* = 3). (**h**, **j**, **l**, **n**) Data are presented as mean ± SD. One-way ANOVA was used to calculate *P*-values. **p**, **q** Expression of CIAPIN 1 in arthritic joints detected by immunohistochemistry. Representative images of three biologically independent samples in each group

To investigate the mechanism by which miRNA 15-29148 promotes chondrocyte apoptosis, we used the miRanda and RNAhybrid algorithms to predict target genes, identifying 14 common targets. By intersecting these with apoptosis-related gene datasets, we identified CIAPIN1 as a key apoptosis-related target (Fig. 4a). CIAPIN1 is a potent apoptosis regulator in mammalian cells, with a conserved miRNA 15-29148 binding site within its 3’UTR in primates (Fig. 4b). This binding site was validated using dual luciferase reporter assays (Fig. 4c). CIAPIN1 expression in cartilage tissues from 18 control participants and 30 patients with RA was assessed using RT-qPCR, western blotting, and immunofluorescence; the results revealed significantly lower CIAPIN1 expression in patients with RA (Fig. 4d, f, g). Correlation analysis of miRNA 15-29148 and CIAPIN1 expression in cartilage tissues from 48 cases indicated an exponential negative correlation (R^2^ > 0.9), suggesting that increased miRNA 15-29148 expression leads to an exponential decrease in CIAPIN1 expression (Fig. 4e).

**Fig. 5:**
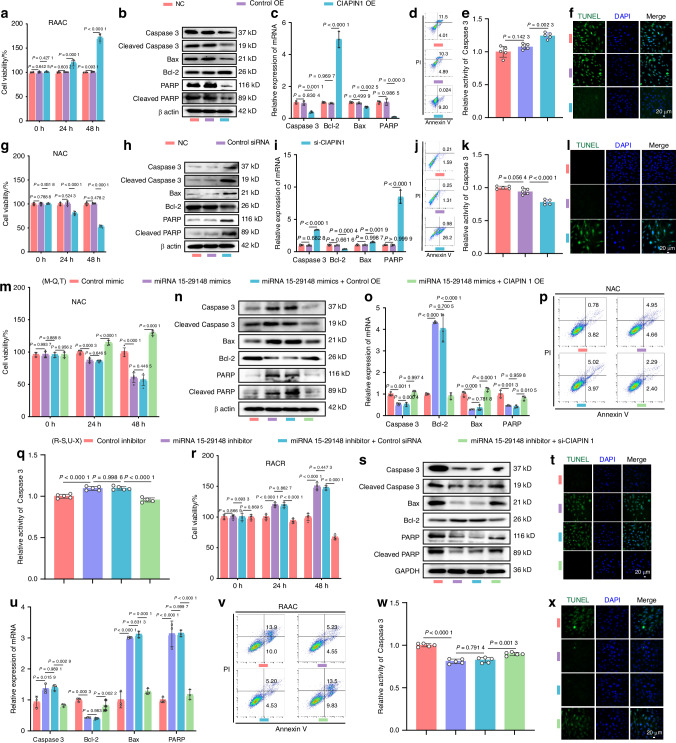
CIAPIN 1 Promotes Apoptosis of Human Primary Chondrocytes. **a**, **g**, **m**, **r** Cell viability was measured using CCK8 at 0, 24 and 48 h (*n* = 5). **b**, **h**, **n**, **s** Western blot analysis detected the expression levels of apoptosis-related proteins. **c**, **i**, **o**, **u** the expression levels of apoptosis-related genes (*n* = 3). **d**, **j**, **p**, **v** Apoptosis was analyzed using flow cytometry with Annexin V-FITC/PI staining (*n* = 3). **e**, **k**, **q**, **w** Caspase-3 activity (*n* = 5). **f**, **l**, **t**, **x** TUNEL staining of RAAC was performed. **a**, **c**, **e**, **g**, **i**, **k**, **n**, **o**, **q**, **r**, **u**, **w** Data are presented as mean value ± SD. One-way ANOVA was used to calculate *P* values

CIAPIN1 expression levels were also examined in chondrocytes transfected with miRNA 15-29148 mimics in NACs and miRNA 15-29148 inhibitors in RAACs (Fig. S16a–d). Expression level of miRNA 15-29148 after transfection with miRNA 15-29148 mimics and inhibitor, and the results were shown in Fig. S17a–d. RT-qPCR and western blotting showed that miRNA 15-29148 mimics suppressed CIAPIN1 expression, while miRNA 15-29148 inhibitors promoted it (Fig. 4h–k). Overexpression of CIAPIN1 after miRNA 15-29148 mimic addition restored CIAPIN1 levels in NACs, while si-CIAPIN1 addition post-miRNA 15-29148 inhibitor reduced CIAPIN1 levels in RAACs (Fig. 4l–o). These results were consistent with the CIAPIN1 immunohistochemistry results from the animal experiments (Fig. 4p, q). To confirm whether miRNA 15-29148 promotes chondrocyte apoptosis through CIAPIN1 inhibition, we assessed apoptotic levels in RAACs after CIAPIN1 overexpression and in NACs after CIAPIN1 silencing. CIAPIN1 overexpression in RAACs significantly reduced apoptosis, whereas CIAPIN1 silencing in NACs increased apoptosis (Figs. 5a–l, S18a, b). We used CCK8 and RT-qPCR to assess AC proliferation. Both methods showed that RASF-SEVs and miRNA 15-29148 inhibit AC proliferation, with decreased expression of Ki67 and Ki67 and proliferating cell nuclear antigen (PCNA) markers (Fig. S19a–f). Additionally, overexpressing CIAPIN1 in high miRNA 15-29148 conditions decreased chondrocyte apoptosis, while silencing CIAPIN1 in low miRNA 15-29148 conditions increased apoptosis (Figs. 5m–x, S18c, d).

These findings strongly suggest that miRNA 15-29148 promotes chondrocyte apoptosis by inhibiting CIAPIN1 expression, underscoring the critical role of CIAPIN1 in the apoptotic pathway regulated by miRNA 15-29148.

### Therapeutic effect of NPs/miRNA 15-29148 antagomir in experimental rheumatoid arthritis models

**Fig. 6:**
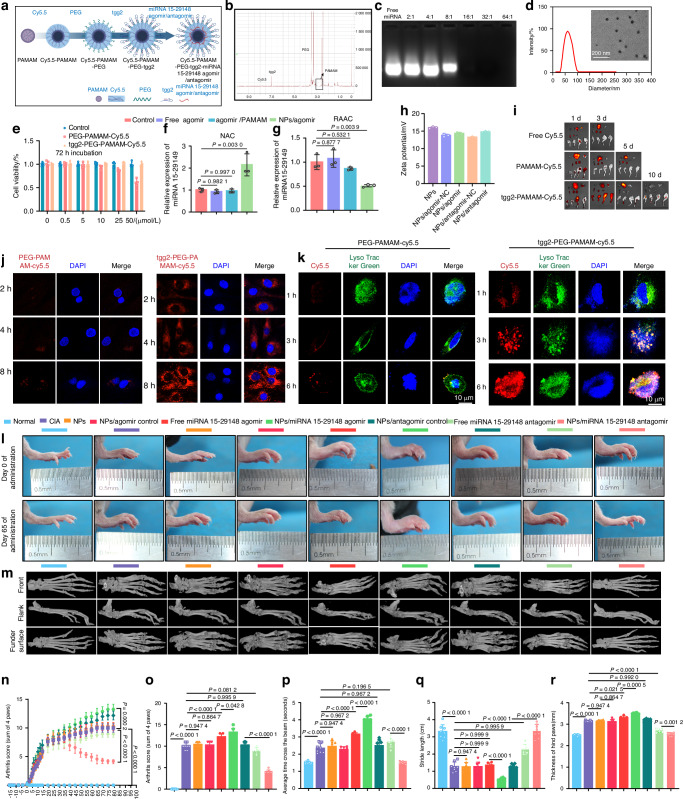
Bone Function and Mobility Analysis of CIA Mice after tgg2-PEG2000-PAMAM6.0-Cy5.5/miRNA 15-29148 Antagomir Delivery. **a** Schematic representation of tgg2-PEG2000-PAMAM6.0-Cy5.5 synthesis. **b** ^1H NMR spectrum of nanoparticles (NPs). **c** Electrophoretic mobility analysis of miRNA in agarose gel. **d** TEM image and NTA results of the NPs/miRNA at a 16:1 weight ratio. **e** In vitro viability of chondrocytes (*n* = 5). **f**, **g** Relative mRNA expression of miRNA 15-29148 in NAC and RAAC (*n* = 3). **h** Zeta potential changes of the resulting nanocomplexes monitored under different mixtures. **i** nanoparticles vivo biodistribution in mice. **j**, **k** Representative images in NAC after 2, 4 and 8 h of transfection with PEG-PAMAM-Cy5.5 or tgg2-PEG-PAMAM-Cy5.5. **l** Evaluation of claw clinical indicators (*n* = 8). **m** Clinical indexes of the anterior and posterior paws (*n* = 8). **n** Beam walking test. **o** Step length measured by hind paw distance. **p** Hind paw thickness (*n* = 8). **q** Severity of soft tissue swelling and bone erosion in the paws of mice evaluated by macroscopic observation. **r** Representative microCT images of a mouse foot paw. **e**–**h**, **m**–**p** Data are presented as mean ± SD. One-way analysis of variance and LSD test were used for statistical analysis

To investigate the in vivo properties of miRNA 15-29148, we employed a targeted nanocarrier system to enhance its stability and delivery efficiency. Dendritic PAMAM, known for its effective gene delivery properties, was modified with the chondrocyte-targeting aptamer tgg2 to synthesize tgg2-PEG-PAMAM-Cy5.5 NPs (Fig. 6a, b). Various weight ratios of NPs to miRNA 15-29148 agomir or antagomir were tested to optimize complexation, with a 16:1 ratio showing the complete disappearance of free miRNA (Fig. 6c). Dynamic light scattering and TEM confirmed that the NPs were spherical with an average size of approximately 60 nm (Fig. 6d). Biocompatibility assessments demonstrated that the NPs exhibited minimal cytotoxicity after 72 h of co-incubation at a concentration of 50 μmol/L (in contrast to PEG-PAMAM-Cy5.5, which showed significant cytotoxicity) (Fig. 6e). In vivo targeting was evaluated by injecting free Cy5.5, PAMAM-Cy5.5, or NPs into mice via arthrocentesis. Free Cy5.5 and PEG-PAMAM-Cy5.5 lost their signals within 1 and 3 days, respectively, while NPs retained a detectable signal for up to 10 days around the foot (Fig. 6i). In vitro studies demonstrated that NPs showed significant signals in human primary chondrocytes after 2 h of co-incubation, whereas PEG-PAMAM-Cy5.5 showed delayed expression after 8 h (Fig. 6j). The stability of the delivery system after complexation with miRNA 15-29148 agomir or antagomir was assessed by examining the zeta potentials of the NPs before and after complexation. Although there was a slight decrease in potential post-complexation, the absolute value remained above 14 (Fig. 6h). To evaluate the delivery efficiency, equal amounts of free miRNA 15-29148 agomir or antagomir were compared with NP-complexed miRNA in NACs and RAACs, respectively. NPs/miRNA 15-29148 agomir significantly increased miRNA 15-29148 expression in NACs, while NPs/miRNA 15-29148 antagomir decreased its expression in RAACs (Fig. 6f, g).

The internalization of NPs into chondrocytes via endosomes/lysosomes was investigated using PEG-PAMAM-Cy5.5 or tgg2-PEG-PAMAM-Cy5.5, which demonstrated co-localization with LysoTracker Green at 3 h and successful escape from the endosomes/lysosomes at 6 h, with tgg2-PEG-PAMAM-Cy5.5 showing higher internalization and escape rates than PEG-PAMAM-Cy5.5 (Fig. 6k). These findings indicate that NPs effectively deliver miRNA 15-29148 to chondrocytes with high biocompatibility and targeted efficiency, highlighting their potential for therapeutic applications in RA. We used RT-qPCR to assess miRNA 15-29148 expression in various mouse cell types before and after CIA induction. Normally, it’s highly expressed in SFs and macrophages, moderately in ACs. In CIA, its expression increases significantly in SFs and NACs (Fig. S20a). Next, we examined the effect of different miRNA 15-29148 agomir formulations on cell expression 48 h post intra-articular administration. Free miRNA 15-29148 agomir slightly elevated expression in five cell types, including SFs, ACs, bone marrow mesenchymal stem cells (BMSC), synovial macrophages (SM), and bone marrow mononuclear cells (BMM) (Fig. S20b). sEV-miRNA 15-29148 agomir significantly increased expression in ACs and SFs, likely due to the autocrine effect of sEVs. In contrast, NPs/miRNA 15-29148 agomir, targeting chondrocytes specifically with aptamers, significantly elevated expression only in ACs.

Meanwhile, we further investigated the stability and resistance to degradation of miRNA 15-29148. The results presented in Fig. S21a, b, indicate that free miRNA 15-29148 undergoes degradation within 6 h in synovial fluid, with over 90% degraded after 1 day. In contrast, significant degradation of sEV-miRNA 15-29148 occurs on the third day. However, NPs/miRNA 15-29148 exhibits no significant degradation even on the seventh day. In summary, NPs/miRNA 15-29148 demonstrates robust stability and resistance to degradation, whereas free miRNA 15-29148 exhibits the poorest stability and resistance to degradation.

To evaluate the regulatory effects of miRNA 15-29148 on RA, we administered NPs containing miRNA 15-29148 agomir or miRNA 15-29148 antagomir via the joint cavity in a mouse model of CIA (Fig. S22a). After 79 days of treatment, we assessed RA-related behavioral indicators. The severity of arthritis was evaluated by clinical index scores throughout the treatment period and before euthanasia. We found no significant difference between the NP, NPs/agomir control, and NPs/antagomir control groups compared to the CIA group. Although the agomir of free miRNA 15-29148 to some extent promoted the progression of arthritis in mice, the antagomir of miRNA 15-29148 can inhibit the continued progression of arthritis in mice. However, NPs/miRNA 15-29148 agomir significantly exacerbated arthritis severity in the mice, while NPs/miRNA 15-29148 antagomir significantly ameliorated arthritis severity (Fig. 6l, n, o). Joint mobility was assessed using the beam walking test and footprint assay. In the beam walking test, normal mice passed smoothly (average of 1.5 s), while CIA mice required an average of 2.24 s. There was no significant difference between the NP, NPs/agomir control, and NPs/antagomir control groups compared to the CIA group. However, mice treated with NPs/miRNA 15-29148 agomir showed prolonged walking times (3.37 s), and some mice were unable to complete the test. In contrast, NPs/miRNA 15-29148 antagomir reduced the walking time to approximately 1.5 s, similar to that observed for normal mice (Fig. 6p). Additionally, the footprint assay showed that post-treatment hind paw stride was reduced in mice treated with NPs/miRNA 15-29148 agomir, while it was significantly increased in mice treated with NPs/miRNA 15-29148 antagomir (Fig. 6q). Measurement of hind paw thickness with calipers revealed a 125% increase in the CIA group compared to that in the normal group. There was no significant difference between the NP, NPs/agomir control, and NPs/antagomir control groups compared to the CIA group. However, NP/miRNA 15-29148 agomir increased hind paw thickness by 140% (compared to the normal group), whereas NPs/miRNA 15-29148 antagomir reduced hind paw thickness by 135% compared to the CIA group (Fig. 6r).

As bone destruction is a prominent feature of RA, we performed three-dimensional (3D) micro-CT reconstruction of bone to assess whether miRNA 15-29148 antagomir could mitigate bone destruction in CIA mice. We reconstructed the foot paw, knee joint, and femur in 3D, revealing that NPs/miRNA 15-29148 agomir significantly promoted bone destruction in these areas, especially deforming the calcaneus of the foot paw. In contrast, treatment with NPs/miRNA 15-29148 antagomir significantly restored bone integrity in CIA mice (Figs. 6m, S23a, S25a). Bone morphometric analysis further showed that NPs/miRNA 15-29148 agomir significantly decreased bone mineral density, bone volume fraction, trabecular number, and trabecular thickness while increasing trabecular separation. In contrast, NPs/miRNA 15-29148 antagomir improved all these parameters (Fig. S25b–f).

**Fig. 7:**
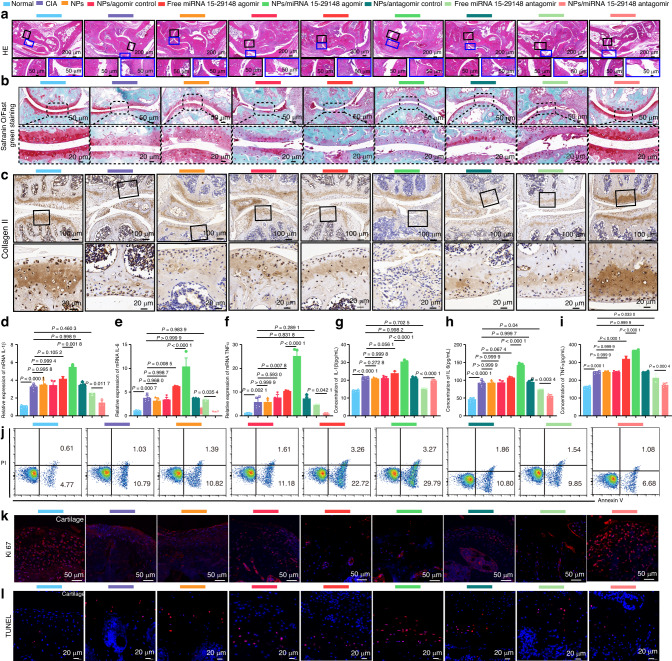
tgg2-PEG2000-PAMAM6.0-Cy5.5/miRNA 15-29148 Antagomir Nanoparticles Reverse Cartilage Damage, Bone Erosion, and Inflammation Levels in CIA Mice. **a** Histological changes in the ankle joint analyzed by H&E staining. **b** Articular cartilage of the ankle identified by Safranin O/Fast Green staining. **c** Expression of Collagen II in arthritic joints detected by immunohistochemistry. **d**–**f** Relative mRNA expression levels of IL1β, IL6 and TNF-α in knee tissue detected by RT-qPCR after 8 weeks of administration. **g**–**i** Serum concentrations of proinflammatory cytokines (IL1β, IL6, TNF-α) measured by ELISA after 8 weeks of administration. **d**–**i** Data are expressed as mean ± SD (*n* = 5). One-way analysis of variance and LSD test were used for statistical analysis. **j** Apoptosis analysis of CIA mouse chondrocytes after 8 weeks of administration using flow cytometry based on Annexin V-FITC/PI staining (*n* = 3). **k**, **l** Anti-proliferation and anti-apoptosis effects of NPs/miRNA 15-29148 antagomir nanoparticles on the synovial tissue or cartilage tissue of CIA mice observed by TUNEL and Ki67 immunofluorescence staining

Synovial tissue hyperplasia and the destruction of cartilage and bone tissue are predominant pathological features of RA. Therefore, we conducted histopathological analyses of mouse ankle joints, including H&E staining. The results revealed that CIA significantly promoted synovial tissue proliferation and cartilage destruction compared to the normal group. Furthermore, treatment with NPs/miRNA 15-29148 agomir exacerbated RA progression, resulting in more severe synovial tissue hyperplasia and cartilage destruction (Fig. 7a). To further investigate the regulatory role of miRNA 15-29148 in the context of cartilage tissue in CIA mice, Safranin O/Fast Green staining and Collagen II immunohistochemistry were performed on mouse ankle joints. The findings showed that NPs/miRNA 15-29148 agomir significantly promoted cartilage destruction compared to that in the CIA group mice, while NPs/miRNA 15-29148 antagomir markedly restored cartilage tissue destruction in CIA mice (Fig. 7b, c). To explore the effects of miRNA 15-29148 on bone tissue in CIA mice, we performed osteocalcin immunohistochemistry on the subchondral bone of mouse knee joints. We found that NPs/miRNA 15-29148 agomir significantly exacerbated bone tissue destruction compared to that in the CIA group mice, whereas NPs/miRNA 15-29148 antagomir effectively restored bone tissue integrity in CIA mice (Fig. S22b). Furthermore, the levels of inflammatory factors in serum and joint tissues were assessed to evaluate inflammation in RA. ELISA and RT-qPCR showed that NP/miRNA 15-29148 agomir significantly elevated the levels of inflammatory markers in CIA mice (compared to those in the CIA group), while NPs/miRNA 15-29148 antagomir significantly reduced the inflammation levels (Fig. 7d–l).

To investigate the role of miRNA 15-29148 in regulating chondrocyte apoptosis in CIA mice, knee chondrocytes were isolated and analyzed. TUNEL assay and Ki67 immunofluorescence of the knee joints was performed to assess synoviocyte proliferation and chondrocyte apoptosis. The expression levels of miRNA 15-29148 in knee chondrocytes were validated, showing that NPs/miRNA 15-29148 agomir significantly increased miRNA 15-29148 levels in the knee chondrocytes of CIA mice (compared to those in the CIA group), whereas NPs/miRNA 15-29148 antagomir decreased miRNA 15-29148 levels (Fig. S24b). RT-qPCR, Flow cytometry, Ki67, and TUNEL analyses indicated that NPs/miRNA 15-29148 agomir significantly promoted synoviocyte proliferation and chondrocyte apoptosis, whereas NPs/miRNA 15-29148 antagomir exhibited opposite effects (Figs. 7j, l, S24a, c–f). The serum levels of ALT, AST, ALP, CREA, and BUN, along with H&E staining of major organs (heart, liver, spleen, lungs, and kidneys), showed that NPs/miRNA 15-29148 agomir/antagomir did not impair liver and kidney function or cause lesions in major organs (Figs. S26a–e, S27).

These findings suggest that miRNA 15-29148 plays a crucial role in regulating RA progression and bone destruction in a CIA mouse model, highlighting its potential as a therapeutic target in RA treatment.

### Preventive effects of miRNA 15-29148 inhibitor against joint destruction in CIA

To evaluate the potential of miRNA 15-29148 antagomir in preventing collagen-induced RA, we first injected NP/miRNA 15-29148 antagomir into the articular cavity of DBA mice four times (once per week). The DBA mice were initially immunized with complete adjuvant, and were injected with collagen 28 days later. The mice then received two more injections of NPs/miRNA 15-29148 antagomir, followed by a final booster immunization with collagen and incomplete adjuvant. Behavioral experiments and subsequent biological assessments related to RA were performed 60 days after the initial immunization (Fig. S28a).

**Fig. 8:**
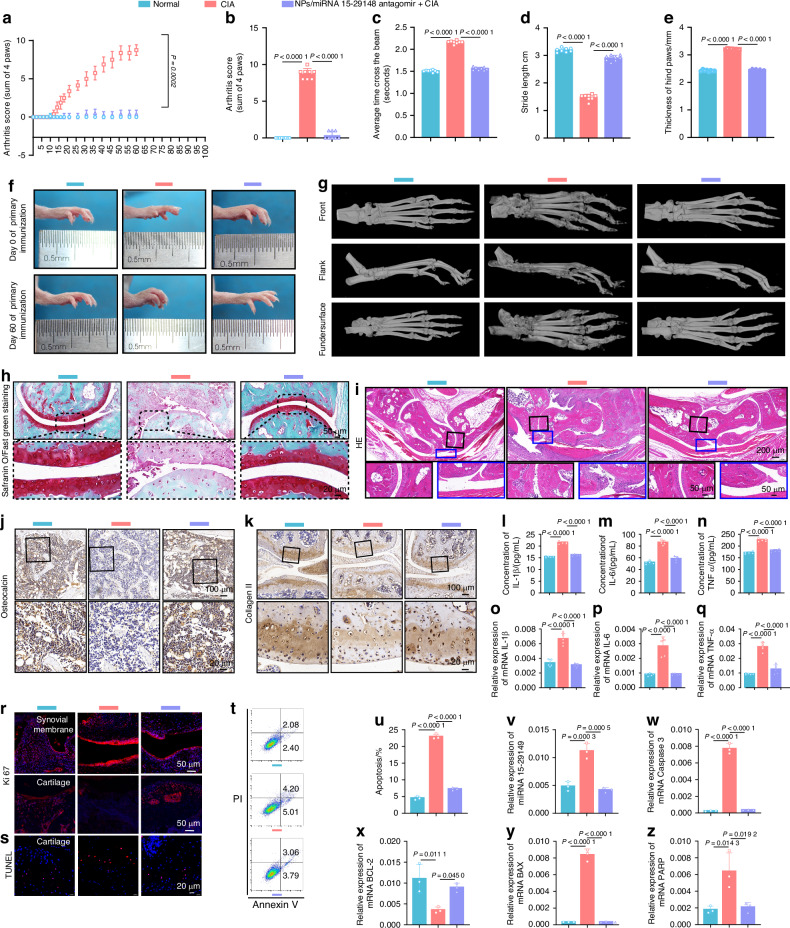
tgg2-PEG2000-PAMAM6.0-Cy5.5/miRNA 15-29148 Antagomir Can Prevent the Occurrence of Arthritis in Mice. **a** Changes in clinical scores within 60 days of initial immunization. (*n* = 8). **b** clinical scores of CIA mice. (*n* = 8). **c** Beam walking test in CIA mice, with the time to cross the 72 cm beam recorded. **d** Step length measured by hind paw distance. **e** Hind paw thickness. (*n* = 8). **f** Severity of soft tissue swelling and bone erosion in the paws of mice evaluated by macroscopic observation. **g** Representative microCT images of a mouse foot paw. **h** Articular cartilage of the ankle identified by Safranin O/Fast Green staining. **i** Histological changes in the ankle joint analyzed by H&E staining. **j**, **k** Expression of Osteocalcin and Collagen II in arthritic joints detected by immunohistochemistry. **l–n** Serum concentrations of proinflammatory cytokines (IL1β, IL6, TNF-α) measured by ELISA after 60 days of initial immunization. **o**–**q** Relative mRNA expression levels of IL1β, IL6, and TNF-α in knee tissue (*n* = 5). **s**, **r** Proliferation and apoptosis effects of NPs/miRNA 15-29148 antagomir nanoparticles on the synovial tissue or cartilage tissue of CIA mice observed by TUNEL and Ki67 immunofluorescence staining. **t**, **u** CIA mouse chondrocyte apoptosis analysis using flow cytometry based on Annexin V-FITC/PI staining (*n* = 3). **v–z** Relative mRNA expression of miRNA 15-29148 in cartilage. Relative mRNA expression of Caspase-3, BCL-2, BAX, and PARP in cartilage measured by RT-qPCR (*n* = 3). **a**–**e**, **l**–**q**, **u**–**z** Data are presented as mean ± SD. One-way analysis of variance and LSD test were used for statistical analysis

Arthritis scores showed that the CIA group mice exhibited RA symptoms starting 10 days post-immunization, with scores reaching 9 by day 50. In contrast, NPs/miRNA 15-29148 antagomir-treated mice did not show significant RA symptoms following their immunizations (both initial and booster) (Fig. 8a, b). The beam walking test and footprint assay revealed that untreated mice took an average of 2.16 s to pass, whereas NPs/miRNA 15-29148 antagomir-treated and normal mice took 1.50 and 1.56 s, respectively (Fig. 8c). The footprint assay also showed that NPs/miRNA 15-29148 antagomir-treated mice had stride lengths similar to mice of the normal group, while untreated mice exhibited more than a two-fold reduction in stride length compared to the normal group (Fig. 8d). Hind paw thickness in untreated mice increased by 132%, whereas there was no significant difference in it between the prophylactic treatment group and normal group (Fig. 8e, f). Three-dimensional micro-CT reconstruction showed that NPs/miRNA 15-29148 antagomir prophylaxis preserved foot paw, knee joint, and femur integrity in CIA mice, while untreated mice exhibited varying degrees of bone damage (Figs. 8g, S28b, S29a). Bone morphological analysis indicated that NPs/miRNA 15-29148 antagomir significantly increased the bone mineral density, bone volume fraction, trabecular number, and trabecular thickness (compared to those in the untreated group) and decreased the trabecular separation to levels similar to those observed in the normal group (Fig. S29b–f).

Histological examinations with H&E staining revealed that prophylactic NP/miRNA 15-29148 antagomir treatment prevented the synovial tissue hyperplasia and cartilage and bone destruction observed in untreated mice (Fig. 8i). Safranin O/Fast Green staining and Collagen II immunohistochemistry of mouse ankle joints showed that NPs/miRNA 15-29148 antagomir significantly inhibited cartilage tissue destruction (compared to that in the untreated group) (Fig. 8h, k). Immunohistochemistry of osteocalcin in the subchondral bone of mouse knee joints revealed that NPs/miRNA 15-29148 antagomir prevented the bone destruction caused by CIA (Fig. 8j). ELISA and RT-qPCR indicated that prophylactic administration of NPs/miRNA 15-29148 antagomir prevented the increase in inflammatory factor levels in the serum and joint tissues, maintaining levels comparable to those in the normal group (Fig. 8l–q). Ki67 and TUNEL staining of knee tissues showed that NPs/miRNA 15-29148 antagomir prophylaxis prevented abnormal synovial proliferation and chondrocyte apoptosis compared to the non-treated group (Fig. 8r, s). Flow cytometry and RT-qPCR of knee chondrocytes further confirmed that NPs/miRNA 15-29148 antagomir prevented chondrocyte apoptosis (Fig. 8t–z). The serum levels of ALT, AST, ALP, CREA, and BUN, along with H&E staining of major organs (heart, liver, spleen, lungs, and kidneys), showed that NPs/miRNA 15-29148 antagomir did not impair liver and kidney function or cause lesions in major organs (Fig. S30a–e, S31a). These findings collectively suggest that miRNA 15-29148 antagomir NPs targeting cartilaginous tissue may prevent Collagen II and Freund’s adjuvant-induced RA in a mouse model.

## Discussion

Current research on RA predominantly centers on studying synoviocytes and chondrocytes individually, as well as investigating the impact of inflammation and immunity on the synovium and cartilage.^27–29^ A substantial network links synovitis and cartilage degradation throughout the advancement of RA.^5^ However, most research primarily focuses on the involvement of inflammatory components from RASFs in the pathology of RA.^30,31^ In this study, we provide evidence of RASF-derived sEVs being potential mediators that are critical to AC apoptosis, contributing to cartilage and bone damage in RA. This study also uncovers miRNA 15-29148 enrichment in RASF-derived sEVs, which act as intercellular mediators to achieve communication with RASFs and ACs. Importantly, we found that increased miRNA 15-29148 levels were correlated with cartilage degradation in RA. We performed a series of in vitro and in vivo studies to confirm that RASF-derived sEVs act by transporting miRNA 15-29148 into ACs to promote cartilage and bone damage, proposing a paradigm of sEV participation in the pathological progression of RA. More importantly, we found that the AC-targeted inhibition of miRNA 15-29148 could exert therapeutic and preventive effects by controlling cartilage and bone damage in a CIA mouse model.

The interaction between the synovium and cartilage induces RA symptoms and may also play a role in the progression of RA.^32,33^ Therefore, elucidating the specific crosstalk mechanism between synovium and cartilage is crucial for in-depth research on the developmental process and pathogenic mechanism of RA.^34^ Interactions between the synovium and cartilage ultimately occur between synovial cells and chondrocytes. In the present study, we developed a co-culture model of RASFs and ACs and found that crosstalk between these two cell types promoted the apoptosis of ACs.

The sEVs originate from endocytosis-mediated invagination of the plasma membrane.^35^ There is increasing evidence that sEVs act as important mediators of cell-to-cell crosstalk and interorgan communication through the delivery of sEV cargoes to target cells.^22,36,37^ SF-secreted sEVs play a crucial role in coordinating the communication between SFs and other key cells in the animal model of RA.^38,39^ Current research mainly focuses on exploring the role of SF-secreted sEVs in regulating communication between SFs and other cells in animal models, but there is a lack of human data.^40^ In this study, we utilized human RASFs and human ACs or ACs from RA to investigate the mechanisms of cell–cell communication. Strikingly, sEVs derived from RASF cultures significantly enhanced AC apoptosis. These findings suggest that RASFs may exert effects on ACs via a non-contact mechanism, possibly by secreting sEVs that promote AC apoptosis, contributing to articular cartilage damage in RA. However, our TUNEL assay results presented an discrepancy, where positive signals did not fully coincide with DAPI staining. This inconsistency could be attributed to several technical factors, including potential issues during the staining process or the presence of non-apoptosis-related DNA damage in the cells. These technical considerations highlight the need for further investigation to clarify this observation.

miRNAs are short RNA molecules that bind to specific target mRNAs, and play a key role in modulating immune and inflammatory responses by targeting genes involved in activation, differentiation, and apoptosis.^23,24^ Thus, we postulated that miRNAs derived from the sEVs isolated from RASFs may promote joint remodeling and bone degeneration. We conducted targeted ultra-trace miRNA sequencing of sEVs, a rarely explored area compared to the traditional RNA sequencing of synovial tissue or synoviocytes. Our analysis identified a novel 18-nucleotide miRNA, miRNA 15-29148, which emerged as a potentially crucial regulator in RA. miRNA 15-29148 mimics significantly promoted apoptosis in ACs and further exacerbated apoptosis in ACs from RA. In contrast, the inhibition of miRNA 15-29148 markedly reduced AC apoptosis, underscoring its pivotal role in chondrocyte apoptosis regulation. To validate these findings, we assessed miRNA 15-29148 expression across more than 45 human knee samples. Interestingly, we found that miRNA 15-29148 expression levels not only correlated with AC apoptosis but also exhibited a significant association with the cartilage degradation of RA. Subsequently, we conducted a study to address whether miRNA 15-29148 released from RASF-derived sEVs could be transferred into ACs to induce AC apoptosis.

To further investigate the in vivo therapeutic potential of miRNA 15-29148, we developed a cationic nanocarrier, tgg2-PEG-PAMAM-Cy5.5, designed for cartilage-targeted delivery.^41–43^ This approach aimed to mitigate interference from other sEV components and address the inherent instability and degradation susceptibility of miRNA.^44,45^ Additionally, the primary and secondary amines of PAMAM within the carrier exhibited enhanced protonation in the acidic environment of the RA joint cavity.^46^ This phenomenon facilitated a proton sponge effect, significantly enhancing the intracellular escape of encapsulated miRNA 15-29148. In vivo experiments demonstrated that NPs/miRNA 15-29148 antagomir effectively treated RA induced by collagen and Freund’s adjuvant. Importantly, prophylactic administration of the drug prevented collagen- and Freund’s adjuvant-induced RA without significant clinical manifestations. These findings indicate that miRNA 15-29148 antagomir holds promise not only for RA treatment but also for RA prevention. This study represents the first exploration of RASF-sEV miRNA 15-29148 and its pivotal role in RA, offering a new perspective on RA pathogenesis and clinical treatment strategies.

This study highlights miRNA 15-29148’s direct role in modulating chondrocyte fate and uncovers a novel pathway in cartilage degeneration. Intriguingly, animal model experiments revealed alterations in inflammation levels, suggesting an indirect effect of miRNA 15-29148 on inflammation within the joint milieu. One plausible explanation is that miRNA 15-29148-induced chondrocyte apoptosis leads to the activating of macrophages and amplifying the inflammatory response.^47,48^ The complexity of the in vivo environment likely contributes to these indirect effects, which were not observable in vitro. Future studies are crucial to elucidate the mechanisms of miRNA 15-29148’s indirect effects on inflammation, particularly its interactions with macrophages and other immune cells. Additionally, investigating miRNA 15-29148’s specific targets and pathways in chondrocytes and joint cells, as well as its therapeutic potential in cartilage degeneration and joint inflammation, is essential. Such studies could provide valuable insights into the pathogenesis of RA and other inflammatory joint diseases, potentially leading to the development of novel therapeutic strategies.

We acknowledge that direct mechanistic evidence demonstrating the effect of miRNA 15-29148 on osteoclasts is absent from our current study. Our findings suggest an indirect effect, possibly via the chondrocyte microenvironment or interactions with other cell types. Future studies will focus on the direct effects of miRNA 15-29148 on osteoclastogenesis and activity to fully understand its role in joint health and disease.^49,50^

Our study found that osteocalcin, a marker of bone formation, is key in rheumatoid arthritis (RA) bone tissue destruction. miRNA 15-29148 regulates bone integrity in a mouse model of RA. The agomir of this miRNA decreased osteocalcin, worsening bone damage, while the antagomir increased it, restoring bone integrity. Osteocalcin affects osteoblasts and osteoclasts, and its modulation by miRNA 15-29148 has broader therapeutic potential in RA, impacting energy metabolism, insulin sensitivity, and muscle function.

To delve deeper into the underlying mechanism by which RASF-derived sEV miRNA 15-29148 promotes chondrocyte apoptosis, we identified and validated its target gene as CIAPIN through database predictions and dual luciferase reporter assays. CIAPIN1 expression was exponentially negatively correlated with miRNA 15-29148. CIAPIN1 acts as a cell death resistance factor by inhibiting apoptosis induced by apoptotic signaling.^51,52^ It forms a complex that interferes with mitochondrial apoptotic processing through intermolecular cooperation with cofactors such as Tah18 or TXNL2.^52^ The role of CIAPIN1 in RA or ACs has not been extensively studied. To investigate its role in the regulation of AC apoptosis, we overexpressed or inhibited CIAPIN1 in ACs. CIAPIN1 significantly inhibited AC apoptosis, whereas its inhibition markedly increased AC apoptosis levels. Experimental evidence from our in vivo studies strongly suggests that CIAPIN1 could be a functional target of RASF-derived sEV miRNA 15-29148 and may mediate its regulatory role in the articular cartilage damage of RA.

Taken together, our study provides a novel finding that sEVs miRNA 15-29148 could serve as an intercellular messenger, mediating SF-to-AC communication to induce AC apoptosis. Our results suggest that targeting sEV miRNA 15-29148 may prove useful in the development of therapeutic or preventive agents against cartilage and bone destruction in RA.

## Materials and methods

### Study design

The primary objective of this study was to investigate the therapeutic and preventive effects of miRNA 15-29148 antagomir targeting chondrocytes in CIA mice. To assess the therapeutic efficacy, normal mice and surgically induced RA models were randomly divided into seven groups: Normal, CIA, NP, NP/agomir control, NP/miRNA 15-29148 agomir, NP/antagomir control, and NP/miRNA 15-29148 antagomir, with each group comprising eight animals. The treatment was administered via intra-articular injection at a dose of 10 µL every 5 days for 80 days, and disease severity was evaluated at the end of this period. To evaluate the preventive effects, normal mice were randomized into three groups: Normal, CIA, and NP/miRNA 15-29148 antagomir. Prophylactic administration was conducted for 6 weeks before inducing the CIA model, with each group also comprising eight animals. The drug was administered intra-articularly at a dose of 10 µL once every 7 days for a total of 8 weeks, and disease severity was assessed 21 days post-modeling. Specific experimental procedures and additional methodological details are provided in the Detailed Experimental Methods section.

### Ethics statement

Animal experiments were performed in accordance with the “Guide for Laboratory Animals” of Jiangnan University and received approval from the Laboratory Animal Ethics Committee of Jiangnan University, Wuxi, China (Approval No. JN. No20240115d0480520[047]). Informed consent was obtained from all patients involved in the study, and the research protocol was approved by the Research Ethics Committee of Anhui Medical University, Hefei, China (Approval No. S20210085).

### Inclusion and exclusion criteria

Patients with RA were diagnosed according to the 2010 American College of Rheumatology/European League Against Rheumatism (ACR/EULAR) classification criteria for RA and were classified based on the Disease Activity Score 28 (DAS28). The inclusion criteria for patients with RA were age >18 years and a disease duration of at least 6 weeks. The exclusion criteria were as follows: (1) a known diagnosis of autoimmune diseases other than RA, (2) a history of severe chronic infections or any current infection, (3) a diagnosis of cancer, (4) shift work, (5) pregnancy and breastfeeding, and (6) receipt of antibiotic treatment within 1 month before participation in this study. For normal/osteoarthritis (OA) controls, individuals had to meet the following inclusion criteria: age >18 years and recent average liver and kidney function screening values. The exclusion criteria were as follows: (1) a known diagnosis of autoimmune diseases, (2) a history of severe chronic infections or any current infection, (3) a diagnosis of cancer, (4) shift work, (5) pregnancy and breastfeeding, and (6) receipt of antibiotic treatment within 1 month before participation in this study.

### Human specimen collection

Knee joint effusions from patients with RA were examined using ultrasound, and synovial fluid was extracted. Control synovial fluid samples were obtained from patients with OA. The exclusion criteria included the presence of cancer, infections, and autoimmune diseases. Synovial and cartilage tissues from patients with RA were collected during arthroplasty procedures, with the exclusion criteria being cancer, infections, and autoimmune diseases, excluding RA. Normal synovial and cartilage tissues were obtained from individuals who underwent amputation due to a car accident or from patients with OA undergoing arthroplasty. The exclusion criteria for these individuals included the presence of cancer, infections, and autoimmune diseases.

### Isolation and passage of human primary synovial fibroblasts

Synovial tissues obtained from knee replacement surgeries of patients with RA or OA were transported to a laminar flow hood within 30 min of collection. The tissues were washed 3–5 times with 75% ethanol and sterile phosphate-buffered saline (PBS) (RG-CE-10, Ketu Biotech). Residual fat and other extraneous tissues were meticulously removed from the synovial tissues using scissors. The cleaned tissues were then placed in Dulbecco’s modified Eagle’s medium (DMEM) high-glucose medium (Gibco) containing 20% fetal bovine serum (FBS; Gibco). The synovial tissues were cut into approximately 25 mm² pieces using sterile ophthalmic scissors and forceps. These pieces were transferred to the bottom of a sterile culture flask with a sterile Pasteur pipette. The culture flasks were inverted, and an appropriate volume of DMEM high-glucose medium with 20% FBS was added. The inverted flasks were carefully placed in an incubator and left undisturbed for 6 h to allow the tissue pieces to adhere to the flask surface. After tissue adherence, the flasks were gently turned upright and placed back in the incubator. The culture medium was not changed for the first 3 days. Starting from day 4, the medium was changed every 3 days until the density of synovial fibroblasts migrating outward from the tissue pieces exceeded 70%. Subsequently, the cells were passaged using trypsin digestion. Human primary synovial fibroblasts from passages 3 to 6 were used for in vitro experiments.

### Isolation and passage of human primary chondrocytes

Cartilage tissues obtained from knee replacement surgeries of patients with RA or OA were promptly transported to a laminar flow hood within 30 min. The tissues were thoroughly washed 3–5 times with 75% ethanol and sterile PBS. Using sterile ophthalmic forceps and scissors, the cartilage tissues were cut into approximately 4 mm² pieces. The tissue pieces were softened by treating them with trypsin at 37 °C for 30 min, with intermittent shaking every 5 min. Following this softening step, the cartilage tissues were digested with 0.3% type II collagenase (Sigma) prepared in DMEM high-glucose medium without FBS for 8–12 h. The resulting cell suspension was then filtered through a sterile 75 μm cell strainer (NEST). The single-cell suspension was centrifuged and subsequently cultured in DMEM high-glucose medium (RG-CE-2, Ketu Biotech) containing 10% FBS. Any undigested tissue pieces were subjected to additional digestion if necessary. Once the cell confluence exceeded 70%, the cells were passaged using trypsin (Gibco) digestion. Human primary chondrocytes from passages 3 to 5 were used for in vitro experiments.

### Identification of primary human chondrocytes

Third-generation primary human chondrocytes were seeded into 6-well plates (Corning). When the cells reached 70%–80% confluence, the medium was discarded, and the cells were washed three times with PBS. The cells were subsequently fixed with 4% paraformaldehyde (Biosharp) for 30 min. Following fixation, the chondrocytes were stained using Toluidine Blue (Solarbio) or subjected to type II collagen immunohistochemistry. The positive rate of the cells was then observed under a microscope.

### Co-culture of primary synovial fibroblasts and primary chondrocytes

Primary synovial fibroblasts and primary chondrocytes from passages 3-5, in their logarithmic growth phase, were selected for co-culture. The cells were washed with PBS and digested with 0.25% trypsin for further use. A transwell plate (Corning) with a pore size of 0.4 μm was employed for the co-culture. Synovial fibroblasts (donor cells) were seeded in the lower chamber, while chondrocytes (recipient cells) were seeded in the upper chamber.

### Cell viability assay

Cell viability was assessed by seeding cells into a 96-well culture plate. The Cell Counting Kit-8 (CCK-8; Sigma-Aldrich) was used to measure viability, which involves the use of 2-(2-methoxy-4-nitrophenyl)-3-(4-nitrophenyl)-5-(2,4-disulfophenyl)-2H-tetrazolium monosodium salt. Absorbance was recorded using a microplate reader (Thermo Scientific, Waltham, MA, USA).

### Annexin V-FITC/PI dual staining for apoptosis detection

Cells were digested with 0.25% trypsin without EDTA, and the digestion was stopped by adding culture medium. The cell suspension was centrifuged at 2 000 r/min for 8 minutes. After centrifugation, the supernatant was discarded, and 1× Annexin V binding buffer was added for another round of centrifugation. The supernatant was discarded again, and 300 μL of 1× Annexin V binding buffer was added to each tube. The cells were resuspended using a pipette and transferred to flow cytometry tubes. In a dark environment, 5 μL of FITC dye (BD Biosciences) was added to the cell suspension and incubated for 15 minutes. Subsequently, 6.5 μL of PI dye (BD Biosciences) was added and incubated for 5 minutes. After incubation, 200 μL of 1× Annexin V binding buffer was added to each tube to bring the total volume to 500 μL. Apoptosis levels were then detected using a flow cytometer (Beckman Coulter, CA, USA).

#### TUNEL staining for apoptosis detection

Cells intended for apoptosis analysis were suspended in PBS buffer at a concentration of 2 × 10^7^ cells/mL. A volume of 50–100 μL of cell suspension was dropped onto poly-L-lysine-coated slides and gently spread using a clean slide after cell adhesion. The cells were fixed with 4% paraformaldehyde solution for 25 minutes. After fixation, the slides were washed twice with PBS for 5 minutes each time, and excess PBS was removed. Cells were permeabilized with 0.2% Triton X-100 solution in PBS at room temperature for 5 minutes. Subsequently, the slides were washed three times with PBS for 5 minutes each. Excess PBS was removed, and 100 μL of 1× Equilibration Buffer was added. The slides were then incubated at room temperature for 30 minutes. The Equilibration Buffer was removed, and 50 μL of TdT reaction buffer (abcam) was added to the slides, followed by incubation at 37 °C for 60 minutes. After incubation, the slides were washed three times with PBS for 5 minutes each in a dark environment. After the final wash, the slides were washed three times with PBS containing 0.1% Triton X-100 and 5 mg/mL BSA to reduce cellular background. Subsequently, 2 μg/mL DAPI solution (Thermo Scientific) was added and incubated for 5 minutes. After three additional washes with PBS, the slides were immediately observed under a confocal laser scanning microscope to visualize green and blue fluorescence.

### Caspase-3 activity analysis

Caspase-3 activity was assessed using the Caspase-3 Colorimetric Assay Kit (R&D Systems) following the manufacturer’s protocol.

### Western Blotting

Protein lysates from tissues or cells were prepared using RIPA buffer supplemented with protease and phosphatase inhibitors. The protein concentration was determined using the BCA Protein Assay Kit (ThermoFisher Scientific). Samples were separated by 10% SDS-PAGE and transferred onto PVDF membranes. The membranes were then incubated overnight at 4°C with specific primary antibodies (refer to Table S1) in TBS-T buffer containing 5% BSA. After washing with TBS-T, the membranes were incubated with HRP-conjugated anti-rabbit IgG or anti-mouse IgG antibodies for 2 h. Immunocomplexes were visualized using an ECL detection kit (ThermoFisher Scientific) and detected by chemiluminescence.

### Total RNA isolation, cDNA synthesis, and RT-qPCR

Total RNA was isolated from tissues or cells using TRIzol reagent (Thermo Scientific, Waltham, MA, USA). The quantity and quality of mRNA were assessed using a nanodrop spectrophotometer (Thermo Scientific, Waltham, MA, USA). cDNA was synthesized with the High-Capacity cDNA Reverse Transcription Kit with RNase Inhibitor (Applied Biosystems, Foster City, CA). For mRNA analysis, RT-qPCR was performed using specific primers and SYBR Green PCR Master Mix (Applied Biosystems) on a QuantStudio 12k Flex System (Applied Biosystems, Foster City, CA). GAPDH was used as a normalization control. The results were analyzed using the comparative Ct (ΔΔCt) method (2^−ΔΔCt^ for logarithmic transformation). For miRNA analysis, reverse transcription and real-time PCR were conducted according to protocols specified by GenePharma (Shanghai, China). U6 was used as the normalization control. All reactions were run on a QuantStudio 12k Flex System (Applied Biosystems, Foster City, CA) and analyzed using the comparative Ct (ΔΔCt) method (2^−ΔΔCt^ for logarithmic transformation). Refer to Table S2 for corresponding primer sequences.

### sEV extraction and characterization

sEV were isolated from cell culture supernatants using differential ultracentrifugation. Briefly, cells were cultured in T75 culture flasks for 48 h to extract sEV. After culturing, the cell culture supernatant was collected and centrifuged at 330 *g* for 10 minutes to remove residual dead cells. The supernatant was transferred to new centrifuge tubes and centrifuged at 3 000 *g* for 30 minutes to remove cell debris. Subsequently, the supernatant was transferred to clean ultracentrifuge tubes and centrifuged at 10 000 *g* for 60 minutes to remove larger vesicles. Following centrifugation, the supernatant was filtered through a 0.22 μm filter and transferred to new ultracentrifuge tubes for ultracentrifugation at 120 000 *g* (Beckman Coulter) for 120 minutes to pellet sEV. After extraction, sEV proteins were lysed directly using a cell lysis buffer, and sEV RNA was extracted using Trizol. sEV were suspended in PBS, collected in enzyme-free centrifuge tubes, and stored at -80°C for up to 7 days. Total sEV protein was quantified using the BCA Protein Assay Kit (ThermoFisher Scientific). sEV identification was performed using several methods: Western blotting to investigate the expression of sEV positive proteins (CD9, CD63, Flotillin-1) and sEV negative proteins (Calnexin, β-actin); nanoparticle tracking analysis to analyze the size distribution of extracted sEV, typically ranging from 30 to 200 nm; and transmission electron microscopy to observe the typical bilayer membrane structure and confirm sizes between 30 and 200 nm. These methods collectively confirmed the standard characterization of the extracted sEV.

### sEV labeling and internalization

PKH26 dye (Sigma-Aldrich) was used to label sEV for tracking and internalization studies. Initially, 80 μL of PKH26 dye at a 1 mmol/L concentration was diluted in 10 mL of Diluent C solution to achieve a final concentration of 8 μmol/L (Solution 1). Subsequently, 1 mg of sEV dissolved in 2 mL of DPBS (Dulbecco’s Phosphate-Buffered Saline) was mixed with 8 ml of Diluent C to obtain Solution 2. Solutions 1 and 2 were combined and gently vortexed with a pipette, followed by incubation at 4 °C for 5 minutes. After incubation, 10 mL of DMEM culture medium containing 10% sEV-free serum was added to bind excess dye. The sEV were then diluted with 100 mL of DPBS and transferred to ultracentrifuge tubes. Using a swing-bucket rotor, the sEV were centrifuged at 120 000 × *g* at 4 °C for 120 minutes. After centrifugation, the supernatant was discarded, and the sEV were resuspended in an appropriate volume of DPBS (Gibco) and washed twice in the centrifuge tube to completely resuspend the dyed sEV. The dyed sEV were filtered through a 0.22 μm filter and added to the culture medium of chondrocytes. Once the sEV were internalized by the chondrocytes, the cells were washed three times with pre-warmed PBS at 37°C to remove the culture medium. To remove any non-internalized sEV, an appropriate amount of green-labeled cholera toxin subunit B was added and incubated for 10 minutes. After incubation, the cells were washed three times with pre-warmed PBS buffer and incubated with DAPI for 10–15 minutes. The cells were then washed three times with PBS and observed using a confocal laser scanning microscope (Zeiss LSM 780) to examine the intensity and distribution of red, green, and blue fluorescence.

### MicroRNA microarray analysis of sEV

High-purity sEV were isolated using differential ultracentrifugation as described previously. Total RNA was extracted from the sEV using TRIzol according to the manufacturer’s instructions. The quality of RNA samples was assessed using a 5300 Bioanalyzer (Agilent), and the quantity was determined with an ND-2000 spectrophotometer (NanoDrop Technologies). Only RNA samples meeting high-quality criteria (OD260/280 = 1.8–2.2, OD260/230 ≥ 2.0, RIN ≥ 6.5, 28S:18S ≥ 1.0, >1 μg) were selected for library construction. RNA purification, reverse transcription, library construction, and sequencing were performed by Majorbio Bio-Pharma Technology Co., Ltd. (Shanghai, China), following Illumina’s recommendations. For each sample, 1 μg of total RNA was used to prepare small RNA libraries using the QIAseq miRNA Library Kit (Qiagen). Activated 5’ and 3’ adapters were ligated to the total RNA, followed by first-strand cDNA synthesis using reverse transcriptase and random primers. PCR amplification was conducted for 11-12 cycles, and the appropriate-sized fragments were separated on a 6% Novex TBE PAGE gel. After quantification with Qubit 4.0, single-end RNA-seq libraries were sequenced using an Illumina NovaSeq Xplus sequencer. Raw reads in fastq format were processed with fastx tools to remove 3’ adapters, poly-N segments, 3’ low-quality bases (Sanger base quality < 20), and sequencing adapters, resulting in clean reads. Identical sequences ranging from 18 to 32 nucleotides were counted and removed from the initial dataset. Bowtie software was used to annotate chromosomal positions according to the reference genome data. Known miRNAs were identified by mapping small RNA tags to the miRBase 22.0 database. Remaining tags were aligned with Rfam and Repbase databases to exclude non-coding RNAs and repeat sequences. Unannotated tags were predicted as novel miRNAs using mirdeep2 based on genomic positions and hairpin structures. The expression levels of each miRNA were calculated using the TPM (transcripts per million reads) method. Differential expression analysis was conducted using DESeq2 or DEGseq, considering |log_2_FC| ≥ 1 and FDR ≤ 0.05 (DESeq2) or FDR ≤ 0.001 (DEGseq) as criteria for significantly differentially expressed miRNAs. Target gene prediction of miRNAs was performed using miRanda, and predicted target genes were annotated using the Gene Ontology (GO) and Kyoto Encyclopedia of Genes and Genomes (KEGG) databases. Functional enrichment analysis, including GO and KEGG pathway analyses, was conducted to identify significantly enriched pathways and GO terms compared to the whole genome background, with a significance level of *P*-adjust ≤ 0.05. Additionally, functional enrichment analysis for graphene oxide was performed using Goatools, and KEGG pathway analysis was conducted using KOBAS.

### Transient transfection

For transient transfection experiments, 200 μL of Opti-MEM medium (Gibco) was added to two sterile 1.5 mL centrifuge tubes labeled as Tube A and Tube B. In Tube A, transfection reagent was added, while in Tube B, the transfection product (miRNA mimics/inhibitors) (GenePharma) was added and allowed to disperse for 5 minutes to ensure thorough mixing. Subsequently, the dispersed transfection product from Tube B was slowly added to the dispersed transfection reagent in Tube A, and the two components were thoroughly mixed for 30 minutes. Within 60 minutes, the mixed solution was slowly and dropwise added to the cells to be transfected, in 2 mL of DMEM high-glucose medium containing FBS, which was added beforehand. For RNA-based experiments, transfection was conducted for 24 h; for protein-based experiments, transfection continued for 72 h. GP-transfect-Mate transfection reagent (GenePharma) was used, with 6.5 μL used per primary human chondrocyte. The miRNA mimics were used in amounts of 9 μL, and the inhibitors of miRNA were used in amounts of 18 μL. Refer to Table S3 for corresponding sequences.

### Luciferase reporter assay

Cells were seeded into a 24-well plate and co-transfected with firefly luciferase reporter constructs encoding wild-type CIAPIN1 3′-UTRs (CIAPIN1-3′-UTR-WT) or mutated CIAPIN1 3′-UTR regions (CIAPIN1-3′-UTR-Mut) (GenePharma), along with miRNA 15-29148 mimics or control mimics, using Lipofectamine 3000 (Invitrogen). Firefly and Renilla luciferase activities were measured using the Dual-Luciferase Reporter Assay Kit (Beyotime) according to the manufacturer’s instructions. All experiments were performed in triplicate to ensure statistical reliability.

### Mice

Male DBA/1J WT mice, aged 8-10 weeks, were obtained from Nanjing JiCui Biotechnology Co., Ltd. (Nanjing, China). All mice were housed under specific pathogen-free conditions with a 12:12-h light/dark cycle. The mice were utilized for collagen-induced arthritis (CIA) and complete/incomplete Freund’s adjuvant-induced arthritis experiments.

### Induction of Collagen-Induced Arthritis (CIA) Model

Collagen-induced arthritis (CIA) was induced in DBA/1J mice on days 1 and 21. Briefly, 100 μg of bovine type II collagen (Chondrex, Redmond, WA, USA) emulsified in an equal volume of complete Freund’s adjuvant (Chondrex) was subcutaneously injected at the base of the tail. On day 21, a second immunization was given with 100 μg of bovine type II collagen emulsified in an equal volume of incomplete Freund’s adjuvant (Chondrex). Following the second immunization, arthritis severity was assessed using the following scoring criteria: 0, no evidence of erythema and swelling; 1, erythema and mild swelling confined to the digits; 2, erythema and mild swelling extending from the ankle to the midfoot (tarsals); 3, moderate swelling and erythema extending from the ankle to the entire foot (metatarsals); 4, severe erythema and swelling encompassing the ankle, foot, and digits, resulting in ankylosis and/or deformity. A clinical score (0-16) was calculated by summing the scores from all limbs, assessed every 2 days. Clinical evaluations were performed in a blinded manner, and arthritis scores were analyzed using a two-way analysis of variance.

### Treatment of CIA Mice

DBA/1J mice with established collagen-induced arthritis (CIA) were randomly divided into 6 groups, each consisting of 8 mice. An additional 8 normal DBA/1J mice from the same batch were included as healthy controls, resulting in a total of 7 groups comprising 56 mice. Thirty-five days after initial immunization, CIA mice received 12 intra-articular injections into the joint cavity, administered every 5 days. The treatment groups were as follows: Healthy control group (no treatment), Saline (10 μL, PBS), NPs (5 mg/mL, 10 μL), NPs/agomir control (weight ratio 16:1, 10 μL), NPs/miRNA 15-29148 agomir (weight ratio 16:1, 10 μL), NPs/antagomir control (weight ratio 16:1, 10 μL), NPs / miRNA 15-29148 antagomir (weight ratio 16:1, 10 μL). During the treatment period, clinical parameters of CIA mice were monitored. At the end of the treatment, hind paw thickness was measured using a caliper, and RA-related behavioral assays were conducted.

### Prevention of CIA in Mice

Male DBA/1J WT mice aged 8-10 weeks were randomly divided into 3 groups, each consisting of 8 mice. One group served as the normal control group and did not receive any treatment or CIA induction. The other two groups received intra-articular injections of saline (10 μL) and NPs / miRNA 15-29148 antagomir (weight ratio 16:1, 10 μL) once a week, for a total of 6 times. After the fifth administration, these two groups of mice were subjected to initial CIA induction. After 21 days of initial induction, a booster immunization was performed. The experiment concluded 40 days after the booster immunization. During the process, the clinical parameters of CIA mice were monitored. At the end of the experiment, hind paw thickness was measured using a caliper, and RA-related behavioral assays were conducted.

### Beam walking test

A narrow beam measuring 3.0 cm wide and 75 cm long was positioned 25 cm above a platform. Prior to receiving treatments, all animals underwent training to walk on the beam. Following treatment, the time each animal required to traverse the beam was measured using a stopwatch. Three consecutive trials were conducted, and the average of these three values was calculated as the walking time for each mouse.

### Footprint assay

The plantar surface of each mouse’s hind paw was coated with black ink. Subsequently, the mouse freely walked along a corridor measuring 1 m in length and 10 cm in width, with white paper placed at the bottom. The distance between consecutive hind paw prints was measured using a digital caliper.

### Micro-computed Tomography (micro-CT) analysis

At the conclusion of the treatment period, mice were euthanized, and their hind paws were fixed in 4% (w/v) paraformaldehyde (RG-GD-01, Ketu Biotech) for 24 h. Subsequently, the hind paws were scanned using a micro-CT system with a 9 μm isotropic resolution on Quantum GXμ-CT (PerkinElmer, Ontario, Canada). The acquired images were then subjected to three-dimensional reconstruction and segmentation using MATERIALIZE MIMICS 19.0 software (Materialize, Leuven, Belgium).

### Measurement of serum inflammatory factors

At the conclusion of the animal experiment, whole blood was collected from CIA mice. The blood samples were allowed to clot at room temperature (25 °C) for 30 minutes, followed by centrifugation at 1 500 *g* for 30 minutes to separate the serum. The upper serum layer was carefully collected for further analysis. The expression levels of TNF, IL-1β, and IL-6 in the serum were measured using ELISA kits specific for each cytokine, according to the manufacturer’s instructions (R&D Systems).

### Histopathological analysis of joint tissues

Ankle joint tissues from the arthritis model were collected and fixed in 4% (w/v) paraformaldehyde at 4°C for 24 h. Decalcification was performed using 10% (w/v) EDTA (RG-TG-01, Ketu Biotech) solution at room temperature (25°C) for 3 weeks. After decalcification, the joints were embedded in paraffin, sectioned into 30 μm thick slices, and stained with hematoxylin and eosin (H&E) for general histological analysis. For cartilage evaluation, sections were stained with Safranin O/Fast Green. The stained sections were observed and photographed using an Olympus DP70 microscope (Tokyo, Japan).

### Fluorescence in situ Hybridization (FISH) and Immunohistochemistry (IHC)

Paraffin-embedded tissue sections were deparaffinized and permeabilized with proteinase K at 37 °C for 10 minutes. Endogenous peroxidase activity was blocked with 3% (vol/vol) H_2_O_2_, followed by dehydration and rehydration steps. For FISH, hybridization was performed using a 40 nmol/L double-DIG LNA™ microRNA probe specific for miRNA 15-29148 (Exiqon, Vedbaek, Denmark) at 52°C for 1 h. After washing with 5x SSC buffer, 1x SSC buffer, and 0.2x SSC buffer, the sections were blocked for 15 minutes and incubated with anti-DIG-POD antibody (Roche Applied Science, Indianapolis, IN, USA). Fluorescence detection was carried out using the TSA Plus Fluorescence System (PerkinElmer, Waltham, Massachusetts, USA) according to the manufacturer’s instructions. For IHC, paraffin-embedded tissue sections were deparaffinized in xylene, rehydrated through a graded alcohol series, and rinsed in water. The sections were then incubated with primary antibodies (see Table S1) at room temperature for 1 h, followed by incubation with biotinylated secondary antibodies (see Table S1) for 30 minutes. The staining was visualized using the Vectastain ABC Kit and DAB Peroxidase Substrate Kit (Vector Laboratories).

### Biocompatibility analysis

Upon completion of the animal experiments, blood and major organs were collected from the mice. The serum was separated using the methods described above, and concentrations of aspartate aminotransferase (AST), alanine aminotransferase (ALT), alkaline phosphatase (ALP), creatinine (CREA), and blood urea nitrogen (BUN) were quantified using an Olympus AU400 fully automated clinical chemistry analyzer. Each organ (heart, liver, spleen, lungs, kidneys) was fixed in 4% (w/v) paraformaldehyde and embedded in paraffin. Sections of 5 μm thickness were prepared from each organ and stained with hematoxylin and eosin (H&E) to detect any pathological changes.

### Immunofluorescence staining

Paraffin-embedded tissue sections were deparaffinized by heating in a 60 °C oven for 15 minutes and then hydrated through a series of xylene and graded alcohols. For antigen retrieval, heat-induced epitope retrieval was performed using a vegetable steamer: slides were incubated in 10 mmol/L citrate buffer at 60°C for 40 minutes followed by washing in PBS. At room temperature, cells were permeabilized with 0.1% Triton X-100 in PBS for 5 minutes. Subsequently, the appropriate primary antibodies (refer to Table S1) were applied and incubated overnight. The following day, corresponding fluorescent secondary antibodies (refer to Table S1) and DAPI were applied, and the samples were observed using laser confocal microscopy (Zeiss LSM 780).

### Synthesis and characterization of nanoparticles (NPs)

Forty milligrams of G5.0 PAMAM (Dendritech, Inc, MI, USA) were dissolved in 2 mL of 0.1 mol/L pH 7.4 PBS. Subsequently, 3.0 mg of Cy5.5 NHS ester (Lumiprobe Corporation, Hallandale Beach, FL), dissolved in 100 μL of DMSO, was added to the solution, which was vigorously stirred at room temperature for 24 h. Excess Cy5.5 was removed by ultrafiltration using an Amicon Ultra-15 centrifugal filter unit (MWCO 3000, Millipore, Billerica, MA). Cy5.5-PAMAM was then conjugated with NHS-PEG2000-MAL (Laysan) at a 1:6 ratio in phosphate buffer solution (pH 8.0) at room temperature for 12 h. The resulting conjugate, Cy5.5-PAMAM-PEG-MAL, was purified by ultrafiltration using a 14 kD molecular weight cut-off membrane and freeze-dried for storage at 4 °C. For the next step, 1.2 µmol of Cy5.5-PAMAM-PEG-MAL was dissolved in 5 mL of nuclease-free phosphate buffer solution (pH 7.0) and reacted with 0.6 µmol of SH-tgg2 at 4 °C for 12 h. The resulting product, Cy5.5-PAMAM-PEG-tgg2, was purified by ultrafiltration and stored at 4 °C. For nanoparticle (NPs) preparation, miRNA (15 µL, 1.5 µg) was mixed with tgg2-PEG-PAMAM-Cy5.5 (5 mg/mL) at different mass ratios (2:1, 4:1, 8:1, 16:1, 32:1, and 64:1). The mixture was gently vortexed for 30 seconds and then allowed to stand for 30 minutes to prepare stable tgg2-PEG-PAMAM-Cy5.5/miRNA 15-29148 agomir/miRNA 15-29148 antagomir NPs. Gel retardation assay was performed to determine the optimal dose of tgg2-PEG-PAMAM-Cy5.5 required for complete encapsulation of miRNA. Characterization of NPs was conducted using nuclear magnetic resonance spectroscopy (1H NMR), dynamic light scattering (DLS), and transmission electron microscopy (TEM).

### Gel Retardation Assay

Nanoparticles (NPs) were prepared by mixing miRNA 15-29148 agomir/miRNA 15-29148 antagomir with NPs at different N/P ratios. After incubation at 37°C for 30 minutes, gel retardation analysis was performed using 1% (w/v) agarose gel electrophoresis (120 V, 20 min). Bands were visualized using the Universal Hood II system (Bio-Rad, USA), with a 10 000 bp DNA ladder (Takara, Dalian, China) used as a molecular weight marker. Uncropped gel scans were provided as source data files.

### Cellular uptake and intracellular mechanism analysis of NPs

Chondrocytes were treated with PEG-PAMAM-Cy5.5 or tgg2-PEG-PAMAM-Cy5.5 and incubated at 37 °C for 1–8 h. For one set of cells, after 2, 4 and 8 h of incubation, they were fixed with 4% paraformaldehyde for 30 minutes, stained with DAPI, and observed under a laser confocal microscope (Carl Zeiss Microscopy LLC, Jena, Germany) to assess NP uptake at different time points. For another set of cells, after incubation for 1, 3 and 6 h, they were further incubated at 37°C for 1 h in cell culture medium containing 50 nmol/L DAPI and 100 nmol/L LysoTracker Green. After incubation, cells were fixed with 4% paraformaldehyde and observed under a laser confocal microscope (Carl Zeiss Microscopy LLC) to evaluate the intracellular distribution of NPs at different time points.

### In vivo targeting analysis of NPs

Male DBA/1J WT mice aged 8-10 weeks were intra-articularly injected with Free Cy5.5, PAMAM-Cy5.5, or tgg2-PAMAM-Cy5.5 at a dose of 5 mg/mL, 10 μL. Mice were euthanized on days 1, 3, 5 and 10, and their organs and limbs were dissected. Tissues were examined using the CALIPER IVIS Lumina III in vivo imaging system (PerkinElmer, USA) to detect tissue fluorescence (λex: 640 nm, λem: 680 nm).

### Statistical analysis

Data are presented as the mean ± SD of three independent experiments. Differences between two groups were determined using the Wilcoxon rank-sum test or Student’s t-test, while variance analysis (ANOVA) or the Kruskal–Wallis test was used for comparisons among three groups, based on the data type. Spearman correlation was used to assess the correlation between clinical information and the differential abundance of taxa. All graphs and statistical analyses were performed using GraphPad Prism 9 (GraphPad Software Inc., La Jolla, CA, USA).

## Supplementary information

Supplementary material

## Data Availability

Data are available on reasonable request.
