# Author Correction: Synovial fibroblast derived small extracellular vesicles miRNA15-29148 promotes articular chondrocyte apoptosis in rheumatoid arthritis

**DOI:** 10.1038/s41413-025-00468-3

**Published:** 2025-09-30

**Authors:** Zhenyu Zhang, Lulu Liu, Huibo Ti, Minnan Chen, Yuechun Chen, Deyan Du, Wenjing Zhan, Tongtong Wang, Xian Wu, Junjie Wu, Dong Mao, Zhengdong Yuan, Jingjing Ruan, Genxiang Rong, Feng-lai Yuan

**Affiliations:** 1 Institute of Integrated Chinese and Western Medicine, Affiliated Hospital of Jiangnan University, Jiangsu, China; 2 Biomedical engineering facility of National Infrastructures for Translational Medicine, State Key Laboratory of Complex Severe and Rare Diseases in Peking Union Medical College Hospital, Chinese Academy of Medical Science and Peking Union Medical College, Beijing, China; 3 Nantong First People’s Hospital, Nantong, China; 4 The Key Laboratory of Synthetic and Biological Colloids, Ministry of Education, School of Chemical and Material Engineering, Jiangnan University, Wuxi, China; 5 The Key Laboratory of Anti-Inflammatory and Immune Medicine, Ministry of Education, Anhui Medical University, Hefei, China; 6 Orthopaedic Institute, Wuxi 9th People’s Hospital Affiliated to Soochow University, Wuxi, China; 7 Department of Respiratory and Critical Care Medicine, The First Affiliated Hospital of Anhui Medical University, Hefei, China; 8 Department of Orthopedics, The First Affiliated Hospital of Anhui Medical University, Hefei, China

**Keywords:** Pathogenesis, Bone

Correction to: *Bone Res.* 10.1038/s41413-025-00430-3, published online 12 June 2025

Following the publication of article,^1^ the authors identified unintentional image layout errors in the “WB bands of BAX” in Fig. 3m, t, as well as in the “TUNEL staining results of the NPs/antagomir control group” in Fig. 7l. These image misuses resulted from inconsistencies between the published and accepted versions due to embedded link errors during the process of providing editable figures after the manuscript was taken over. Therefore, corrections to these two sections are required. Despite the necessity of these amendments, they do not alter the conclusions of this study or the overall interpretation of the article.

Original Fig. 3m, t the BAX bands in the Western blot:
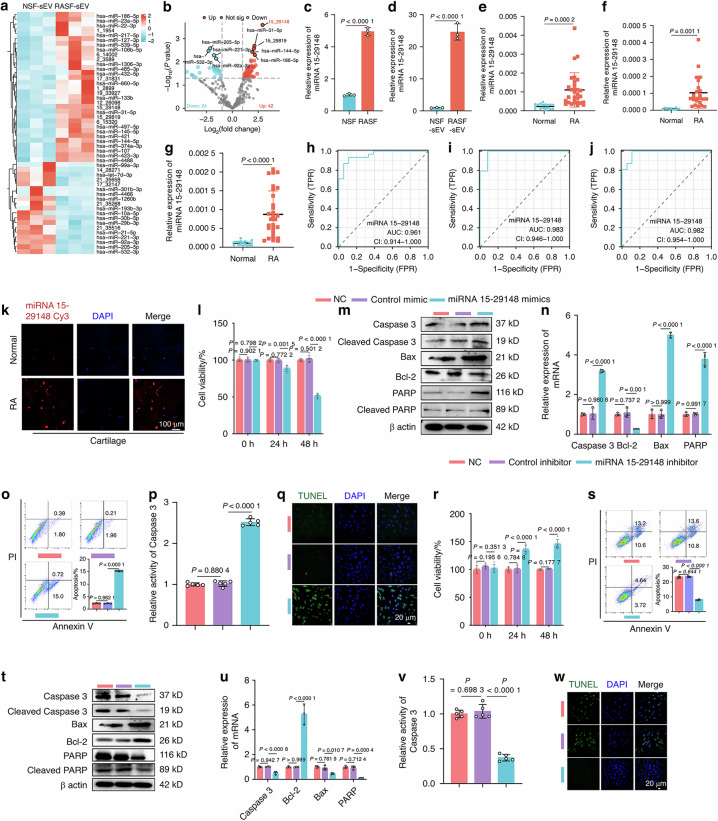


**Fig. 3**. miRNA 15-29148 in SEV Secreted by RASF Promotes Apoptosis of AC. **a** Heat map depicting the top 50 differentially expressed miRNAs in RASF sEV. **b** Volcano plot of miRNA expression level differences between RA patients and controls in sEV. **c**, **d** miRNA 15-29148 expression levels in human primary synovial fibroblasts and their secreted sEVs. **e**–**g** miRNA 15-29148 expression levels in synovial tissues synovial fluid cartilage of RA patients or controls. **c**–**g** All bar graphs are presented as the mean ± SD. by Student’s *t* test. **h**–**j** Sensitivity and specificity of the ROC curve in assessing the prediction of RA by miRNA 15-29148 expression level in synovial tissue, joint fluid and cartilage tissue. **k** FISH analysis of RA cartilage tissues levels of miRNA 15-29148. **l**, **r** CCK8 analysis of NAC and RAAC cell viability (*n* = 5). **m**, **t** Western blot analysis detected the expression levels of apoptosis-related proteins in NCR and RAAC (*n* = 3). **n**, **u** The expression levels of apoptosis-related genes in NAC and RAAC (*n* = 3). **o**, **s** Apoptosis analysis of NAC and RAAC using flow cytometry with Annexin V-FITC/PI staining (*n* = 3). **p**, **v** Caspase-3 activity in NAC and RAAC (*n* = 5). **q**, **w** TUNEL staining of NAC and RAAC. **l**, **n**–**p**, **r**, **s**, **u**, **v** Data are presented as mean ± SD. One-way ANOVA was used to calculate *P*-values

The revised Fig.3m, t the BAX bands in the Western blot should read:
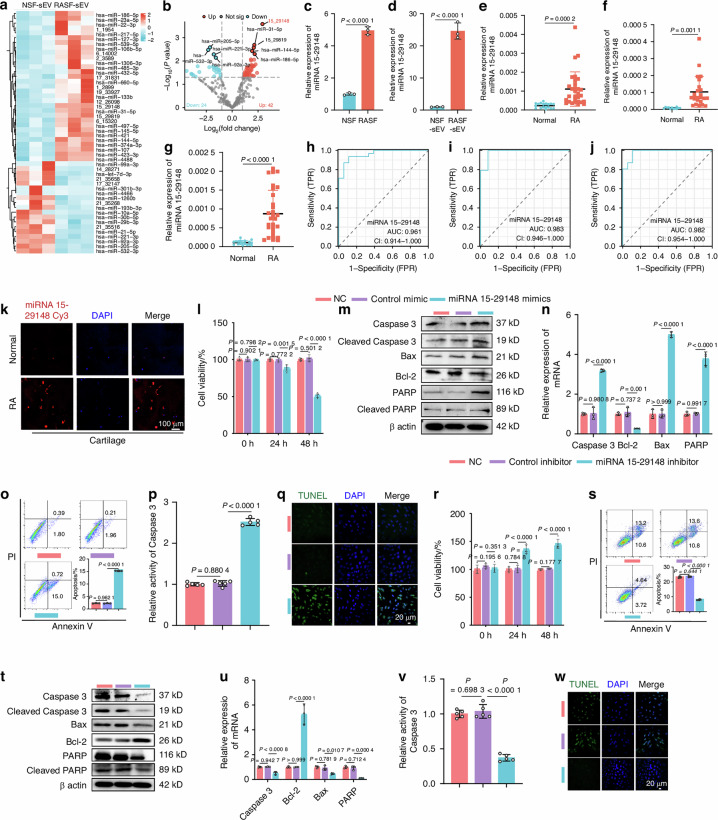


**Fig. 3**. miRNA 15-29148 in SEV Secreted by RASF Promotes Apoptosis of AC. **a** Heat map depicting the top 50 differentially expressed miRNAs in RASF sEV. **b** Volcano plot of miRNA expression level differences between RA patients and controls in sEV. **c**, **d** miRNA 15-29148 expression levels in human primary synovial fibroblasts and their secreted sEVs. **e**–**g** miRNA 15-29148 expression levels in synovial tissues synovial fluid cartilage of RA patients or controls. **c**–**g** All bar graphs are presented as the mean ± SD. by Student’s *t* test. **h**–**j** Sensitivity and specificity of the ROC curve in assessing the prediction of RA by miRNA 15-29148 expression level in synovial tissue, joint fluid and cartilage tissue. **k** FISH analysis of RA cartilage tissues levels of miRNA 15-29148. **l**, **r** CCK8 analysis of NAC and RAAC cell viability (*n* = 5). **m**, **t** Western blot analysis detected the expression levels of apoptosis-related proteins in NCR and RAAC (*n* = 3). **n**, **u** The expression levels of apoptosis-related genes in NAC and RAAC (*n* = 3). **o**, **s** Apoptosis analysis of NAC and RAAC using flow cytometry with Annexin V-FITC/PI staining (*n* = 3). **p**, **v** Caspase-3 activity in NAC and RAAC (*n* = 5). **q**, **w** TUNEL staining of NAC and RAAC. **l**, **n**–**p**, **r**, **s**, **u**, **v** Data are presented as mean ± SD. One-way ANOVA was used to calculate *P*-values

Original Fig. 7l TUNEL staining results of the NPs/antagomir control group:
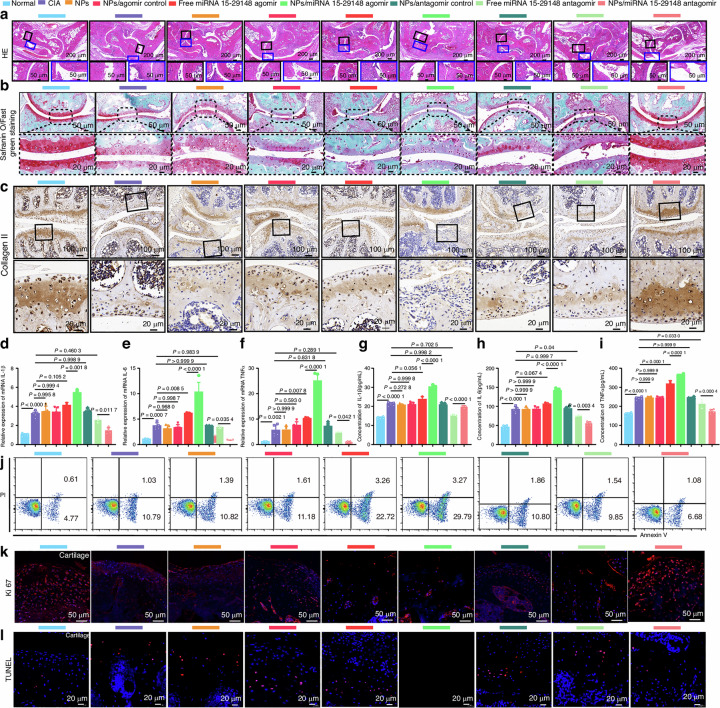


**Fig. 7** tgg2-PEG2000-PAMAM6.0-Cy5.5/miRNA 15-29148 Antagomir Nanoparticles Reverse Cartilage Damage, Bone Erosion, and Inflammation Levels in CIA Mice. **a** Histological changes in the ankle joint analyzed by H&E staining. **b** Articular cartilage of the ankle identified by Safranin O/Fast Green staining. **c** Expression of Collagen II in arthritic joints detected by immunohistochemistry. **d**–**f** Relative mRNA expression levels of IL1β, IL6 and TNF-α in knee tissue detected by RT-qPCR after 8 weeks of administration. **g**–**i** Serum concentrations of proinflammatory cytokines (IL1β, IL6, TNF-α) measured by ELISA after 8 weeks of administration. **d**–**i** Data are expressed as mean ± SD (*n* = 5). One-way analysis of variance and LSD test were used for statistical analysis. **j** Apoptosis analysis of CIA mouse chondrocytes after 8 weeks of administration using flow cytometry based on Annexin V-FITC/PI staining (*n* = 3). **k**, **l** Anti-proliferation and anti-apoptosis effects of NPs/miRNA 15-29148 antagomir nanoparticles on the synovial tissue or cartilage tissue of CIA mice observed by TUNEL and Ki67 immunofluorescence staining

The revised Fig. 7l TUNEL staining results of the NPs/antagomir control group should read:
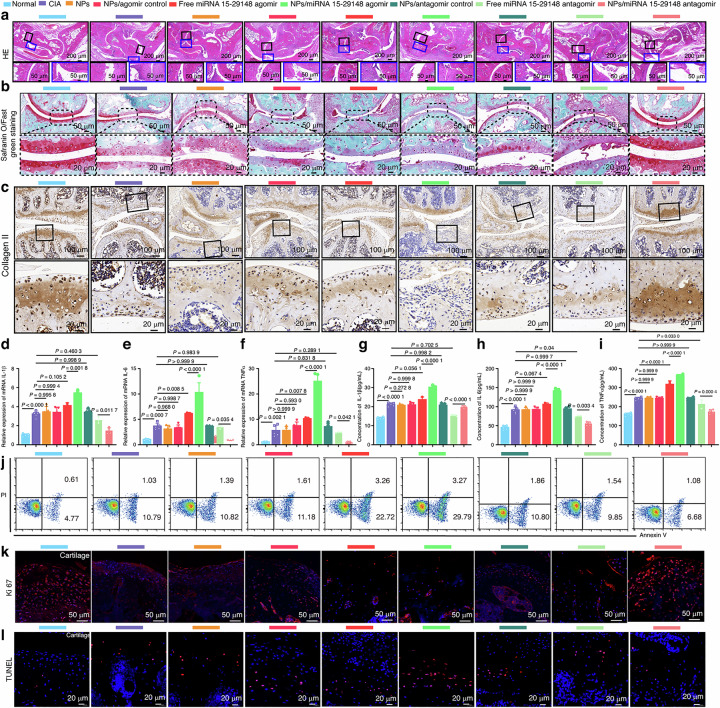


**Fig. 7** tgg2-PEG2000-PAMAM6.0-Cy5.5/miRNA 15-29148 Antagomir Nanoparticles Reverse Cartilage Damage, Bone Erosion, and Inflammation Levels in CIA Mice. **a** Histological changes in the ankle joint analyzed by H&E staining. **b** Articular cartilage of the ankle identified by Safranin O/Fast Green staining. **c** Expression of Collagen II in arthritic joints detected by immunohistochemistry. **d**–**f** Relative mRNA expression levels of IL1β, IL6 and TNF-α in knee tissue detected by RT-qPCR after 8 weeks of administration. **g**–**i** Serum concentrations of proinflammatory cytokines (IL1β, IL6, TNF-α) measured by ELISA after 8 weeks of administration. **d**–**i** Data are expressed as mean ± SD (*n* = 5). One-way analysis of variance and LSD test were used for statistical analysis. **j** Apoptosis analysis of CIA mouse chondrocytes after 8 weeks of administration using flow cytometry based on Annexin V-FITC/PI staining (*n* = 3). **k**, **l** Anti-proliferation and anti-apoptosis effects of NPs/miRNA 15-29148 antagomir nanoparticles on the synovial tissue or cartilage tissue of CIA mice observed by TUNEL and Ki67 immunofluorescence staining

The original article^1^ was updated.
