# Supplementary material for: Synovial fibroblast derived small extracellular vesicles miRNA15-29148 promotes articular chondrocyte apoptosis in rheumatoid arthritis
Source: Bone Res. 2025 Jun 12;13:61. doi: 10.1038/s41413-025-00430-3 (PMC12162823; doi:10.1038/s41413-025-00430-3)
Supplement: Supplementary file 1 — Supplementary material [file 41413_2025_430_MOESM1_ESM.pdf]

Materials and methods

Intra-articular Injection Protocol

Mice were anesthetized using a respiratory anesthesia apparatus and positioned supine upon achieving full anesthesia. The knee joint of one hind limb was flexed manually to maximize exposure of the patellar articular surface. The fur surrounding the knee joint was removed with a shaver, and the site was sterilized sequentially with iodophor and alcohol. A 2-mm bend was introduced at the distal tip of the syringe needle, creating a 9° angle. The needle was then carefully inserted along the angular bisector of the knee joint, with gentle lateral displacement of the patellar suspensory ligament to avoid obstruction. The designated solution was subsequently administered into the joint cavity.

Isolation and Purification of Synovial Macrophages

Synovial tissue was enzymatically digested with type II collagenase, and the resulting suspension was passed through a 70 μm cell strainer to obtain a single-cell suspension. The cells were seeded in cell culture flasks without prior collagen coating. Once the adherent cells reached an appropriate density, they were digested with trypsin. Upon observation of fibroblast detachment under a microscope, the digestion was terminated by carefully adding complete culture medium. The cell suspension containing fibroblasts was aspirated, and fresh medium was added to the remaining adherent cells for continued culture. As synovial fibroblasts are highly sensitive to trypsin, whereas synovial macrophages are resistant, this differential trypsin sensitivity was exploited to selectively remove fibroblasts. The process was repeated 2–3 times to achieve high-purity synovial macrophages.

Isolation and Culture of Bone Marrow Mesenchymal Stem Cells and Bone Marrow Macrophages

The femurs were carefully isolated from mice and thoroughly cleaned 3–5 times with 75% ethanol followed by PBS. The cleaned femurs were placed in 10 mL of culture medium. Using sterile scissors, the ends of each femur were excised, and a syringe was employed to flush the bone marrow from the medullary cavity until the bone tissue appeared white. The collected bone marrow suspension was passed through a 70 μm cell strainer to obtain a single-cell suspension. For the isolation of bone marrow mesenchymal stem cells (BM-MSCs), the single-cell suspension was cultured in medium lacking macrophage colony-stimulating factor (M-CSF) for 24 hours. During this period, adherent cells represented BM-MSCs. The non-adherent cells were removed after 24 hours, centrifuged, and resuspended in medium supplemented with 30 ng/mL M-CSF. The resuspended cells were seeded in new culture flasks and maintained for 48 hours, during which adherent cells were identified as bone marrow macrophages (BMMs). After 48 hours, the non-adherent cells were discarded, and the adherent cells were provided with fresh medium for continued culture until further use.

Cell-Specific Analysis of miRNA 15-29148 In Vivo

Free miRNA 15-29148, sEVs-miRNA 15-29148, or NPs-miRNA 15-29148 were locally administered via intra-articular injection in mice. After 48 hours, bone marrow mesenchymal stem cells (BM-MSCs), bone marrow macrophages/monocytes, and synovial fibroblasts were isolated from the joint tissues. The expression levels of

miRNA 15-29148 were subsequently assessed in chondrocytes and synovial macrophages to determine its cell-specific distribution and activity.

Intra-Articular Injection of sEVs in CIA Mice

To evaluate the regulatory effects of RASF-sEVs on arthritis, 5 μL of NSF-sEVs or RASF-sEVs were administered via intra-articular injection to CIA mice every five days over a 60-day period. Behavioral assessments were conducted following each administration to evaluate therapeutic outcomes. Upon completion of the experimental timeline, the mice were euthanized, and the femur, tibia, and serum were collected for subsequent analyses.

Intra-Articular Injection of Free miRNA 15-29148 Agomir in CIA Mice

To investigate the regulatory effects of free miRNA 15-29148 agomir on arthritis, 5 μL of miRNA 15-29148 agomir or miRNA 15-29148 antagomir was administered to CIA mice via intra-articular injection every five days for a duration of 60 days. Behavioral assessments were conducted after each administration to evaluate therapeutic efficacy. At the conclusion of the study, the mice were euthanized, and the femur, tibia, and serum were collected for further analysis.

In Vitro Stability Analysis of miRNA 15-29148

The stability of miRNA 15-29148 was assessed in vitro using ultracentrifuged supernatant from articular fluid as the medium. Free miRNA 15-29148, sEVs-miRNA 15-29148, or NSF-miRNA 15-29148 were dispersed in the medium and incubated for 0–7 days. At specified time points (day 0, 0.25, 1, 3, 5, and 7), 300 μL of the medium was collected. miRNA 15-29148 was subsequently recovered from the collected samples and quantified using quantitative PCR (qPCR).

Supplementary tables

Supplementary table 1. Antibody Details Table

Antibodies	SOURCE	IDENTIFIER
Caspase-3	Abcam	ab184787
Cleaved Caspase-3	Abcam	ab32042
BAX	Cell Signaling Technology	#5023
BCL-2	Cell Signaling Technology	#15071
PARP	Abcam	ab191217
Cleaved PARP	Abcam	ab32561
β-actin	Cell Signaling Technology	#3700
GAPDH	Cell Signaling Technology	#97166
CIAPIN 1	GeneTex	GTX55572
CD9	Abcam	ab236630
CD63	Abcam	ab271286
Flotillin 1	Abcam	ab78178
Calnexin	Abcam	ab92573
Ostecalcin	Abcam	ab93876
CollagenII	Abcam	ab307674
Caspase-1	Abcam	ab207802
Cleaved Caspase-1	Cell Signaling Technology	#89332S
NLRP3	Abcam	ab263899
Podoplanin	Abcam	ab319138
aggrecan	Abcam	ab3778
Histone H3	Abcam	ab308373
Anti-rabbit IgG, HRP-linked Antibody	Cell Signaling Technology	#7074
Anti-mouse IgG, HRP-linked Antibody	Cell Signaling Technology	#7076
Anti-rabbit IgG (H+L), F(ab')2 Fragment (Alexa Fluor® 488 Conjugate)	Cell Signaling Technology	#4412
Anti-rabbit IgG (H+L), F(ab')2 Fragment (Alexa Fluor® 647 Conjugate)	Cell Signaling Technology	#4414

Supplementary table 2. mRNA/miRNA RT-QPCR primer sequence details

Genes	species	form	Primer sequence (5′-3′)
CIAPIN1	Human	Forward Primer	GAGGAAGTACAGTCTGTTCGAGA
		Reverse Primer	AAGCTGCCTAGAAGAACCCAC
	Mouse	Forward Primer	GGAGTTTGGGATCTCCCCTG
		Reverse Primer	ACCCGACAGAATGACATCGAA
Caspase-3	Human	Forward Primer	CATGGAAGCGAATCAATGGACT
		Reverse Primer	CTGTACCAGACCGAGATGTCA
	Mouse	Forward Primer	CTGACTGGAAAGCCGAAACTC
		Reverse Primer	CGACCCGTCCTTTGAATTTCT
BAX	Human	Forward Primer	CCCGAGAGGTCTTTTTCCGAG
		Reverse Primer	CCAGCCCATGATGGTTCTGAT
	Mouse	Forward Primer	AGACAGGGGCCTTTTTGCTAC
		Reverse Primer	AATTCGCCGGAGACACTCG
BCL-2	Human	Forward Primer	GGTGGGGTCATGTGTGTGG
		Reverse Primer	CGGTTCAGGTACTCAGTCATCC
	Mouse	Forward Primer	GCTACCGTCGTGACTTCGC
		Reverse Primer	CCCCACCGAACTCAAAGAAGG
PARP	Human	Forward Primer	CGGAGTCTTCGGATAAGCTCT
		Reverse Primer	TTTCCATCAAACATGGGCGAC
	Mouse	Forward Primer	GCTTTATCGAGTGGAGTACGC
		Reverse Primer	GGAGGGAGTCCTTGGGAATAC
GAPDH	Human	Forward Primer	GGAGCGAGATCCCTCCAAAAT
		Reverse Primer	GGCTGTTGTCATACTTCTCATGG
	Mouse	Forward Primer	AGGTCGGTGTGAACGGATTTG
		Reverse Primer	GGGGTCGTTGATGGCAACA
IL1β	Mouse	Forward Primer	GAAATGCCACCTTTTGACAGTG
		Reverse Primer	TGGATGCTCTCATCAGGACAG
IL6	Mouse	Forward Primer	TCTATACCACTTCACAAGTCGGA
		Reverse Primer	GAATTGCCATTGCACAACTCTTT
TNF-α	Mouse	Forward Primer	CCACCACGCTCTTCTGTCTAC
		Reverse Primer	GCCATTTGGGAACTTCTCATC
U6	Human	Forward Primer	CAGCACATATACTAAAATTGGAACG
		Reverse Primer	ACGAATTTGCGTGTCATCC
	Mouse	Forward Primer	CTCGCTTCGGCAGCACA
		Reverse Primer	AACGCTTCACGAATTTGCGT
miRNA 15-29148	Human/ Mouse	Forward Primer	AGGAAAGTAGCACAGTGCCAGG
		Reverse Primer	TATGGTTGTTGACGACTGGTTGAC
Has-mir-31-5p	Human	Forward Primer	GTGTTGTTCTAAAGGCAAGATGC
		Reverse Primer	TATGGTTGTTCTCGTCTCTGTGTC
Has-mir-221-3p	Human	Forward Primer	GAAGTTCGTCCAGCTACATTGTCT
		Reverse Primer	TATGGTTGTTCTCGCTCTGTGTC
Has-mir-144-5p	Human	Forward Primer	GCTCATCCACGGATATCATCAT
		Reverse Primer	TATGCTTGTTCTCGTCTCTGTGTC
Has-mir-145-5p	Human	Forward Primer	GCAGGTCCAGTTTTCCCA
		Reverse Primer	TCCAGTTTTTTTTTTTTTTAGGGATTC
Has-mir-523-5p	Human	Forward Primer	GAATCCCTCCTGGCTCACTG
		Reverse Primer	TATGGTTGTTGACGACTGGTTGAC
Has-mir-205-5p	Human	Forward Primer	CACGCCAGGCTCCA
		Reverse Primer	CGGGCCCCCCGAACATT
Has-mir-186-5p	Human	Forward Primer	AAGAATTCTCCTTTTGGGCT
		Reverse Primer	GTGCGTGTCGTGGAGTCG
Has-mir-92a-3p	Human	Forward Primer	AACCGGCCTATTGCACTTGTC
		Reverse Primer	TATGGTTGTTCACGACTCCTTCAC
miRNA 15-29819	Human	Forward Primer	TGCAGTAGGGGAGCTACAGTCTT
		Reverse Primer	TATCCTTCTTCACGACTCCTTCAC

MKI67	Human	Forward Primer	AGAAGAAGTGGTGCTTCGGAA
		Reverse Primer	AGTTTGCGTGGCCTGTACTAA
PCNA	Human	Forward Primer	ACACTAAGGGCCGAAGATAACG
		Reverse Primer	ACAGCATCTCCAATATGGCTGA
NLRP3	Human	Forward Primer	GATCTTCGCTGCGATCAACAG
		Reverse Primer	CGTGCATTATCTGAACCCCAC
Caspase1	Human	Forward Primer	TTTCCGCAAGGTTCGATTTTCA
		Reverse Primer	GGCATCTGCGCTCTACCATC
IL 18	Human	Forward Primer	TCTTCATTGACCAAGGAAATCGG
		Reverse Primer	TCCGGGGTGCATTATCTCTAC
Aggrecan	Human	Forward Primer	CCCCTGCTATTTCATCGACCC
		Reverse Primer	GACACACGGCTCCACTTGAT

Supplementary table 3. sequences for vector construction

Primer	Sequence (5'-3')
Negative control sense	UUCUCCGAACGUGUCACGUTT
Negative control antisense	ACGUGACACGUUCGGAGAATT
INHIBITOR Negative control	CAGUACUUUUGUGUAGUACAA
miRNA 15-29148 mimics sense	AUUCUACCCAGCUGCACC
miRNA 15-29148 mimics antisense	UGCAGCUGGGUAGAAAUU
miRNA 15-29148 inhibitor	UGGCACUGUGCUACUUUCCU
hsa-mir-221-3p mimics sense	AGCUACAUUGUCUGCUGGGUUUC
hsa-mir-221-3p mimics antisense	AACCCAGCAGACAAUGUAGCUUU
hsa-mir-221-3p inhibitor	GAAACCCAGCAGACAAUGUAGCU
hsa-miR-144-5p mimics sense	GGAUAUCAUCAUAUACUGUAAG
hsa-miR-144-5p mimics antisense	UACAGUAUAUGAUGAUAUCCUU
hsa-mir-144-5p inhibitor	CUUACAGUAUAUGAUGAUAUCO
miRNA 15-29819 mimics sense	AUUCUACCCAGCUGCACC
miRNA 15-29819 mimics antisense	UGCAGCUGGGUAGAAUUU
miRNA 15-29819 inhibitor	GGUGCAGCUGGGUAGAAU
SI-CIAPIN1-Homo sense	GCUUCUGAGUGAUAGCAAUTT
SI-CIAPIN1-Homo antisense	AUUGCUAUCACUCAGAAGCTT

Supplementary figure

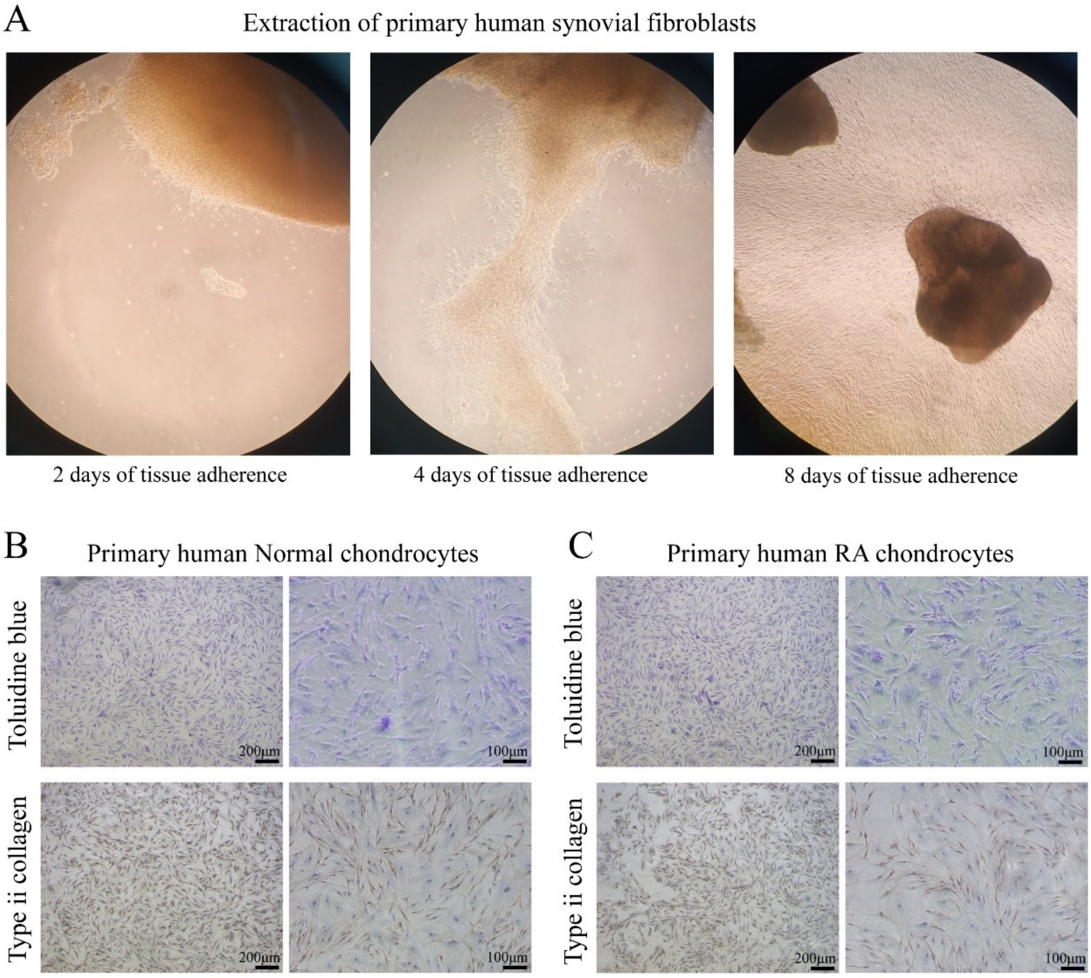

Supplemental Figure 1: Extraction of Human Primary Synovial Cells and Identification of Human Primary Chondrocytes (A) Synovial cells at days 2, 4, and 8 after attachment to synovial tissue. (B) Primary chondrocytes were identified by toluidine blue and collagen II immunohistochemistry. (C) Primary chondrocytes from RA patients were identified using toluidine blue and collagen II immunohistochemistry. Representative images of three biologically independent samples in each group are displayed in panels (A-C).

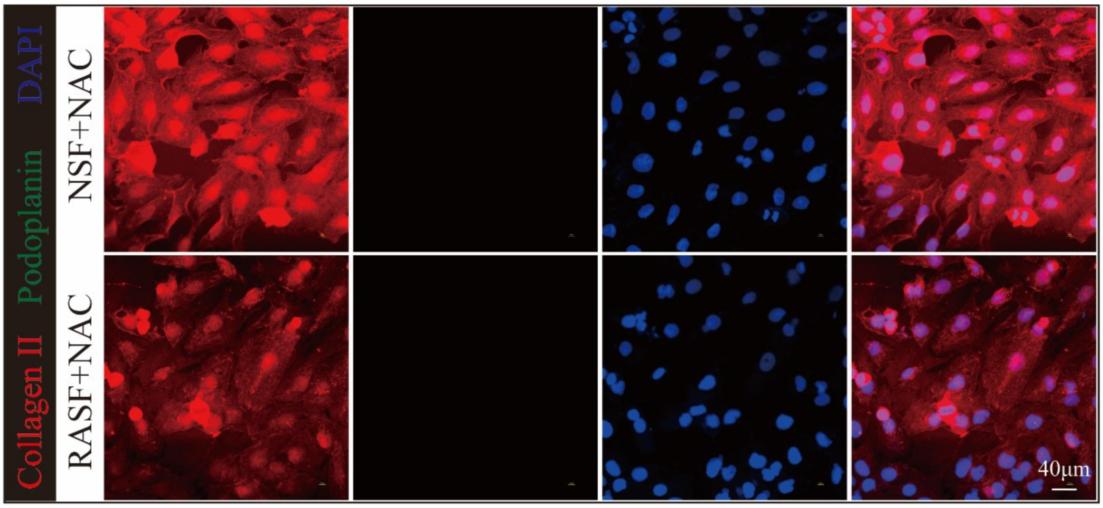

Supplemental Figure 2: Purity verification of recipient cells after co-culture of chondrocytes and synovial fibroblasts. A representative image of three biologically independent samples from each group is shown.

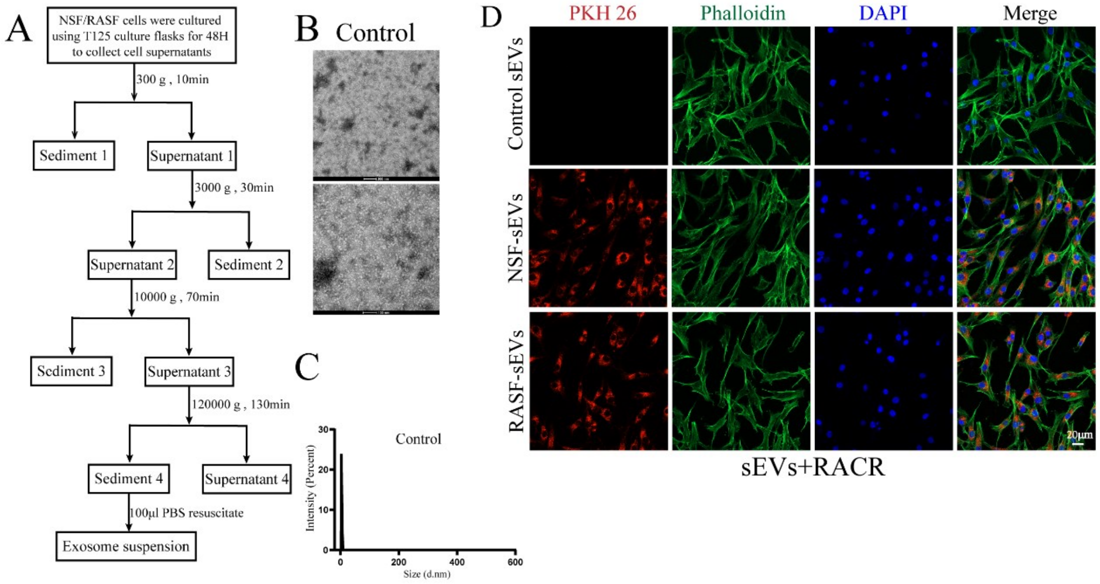

Supplemental Figure 3: Extraction, Identification, and Fusion of RASF Exosomes (A) Detailed flow chart of exosome extraction. (B) Transmission electron microscopy results of the exosome control group. (C) Nanoparticle tracking analysis (NTA) results of the exosome control group. (D) RASF exosomes stained with PKH26 red membrane dye. The RACR cytoskeleton is labeled with Phalloidin (green), and nuclei are counterstained with DAPI (blue). Scale bars, 20 μm. A representative image of 3 biologically independent experiments from each group is shown in panels (B, D).

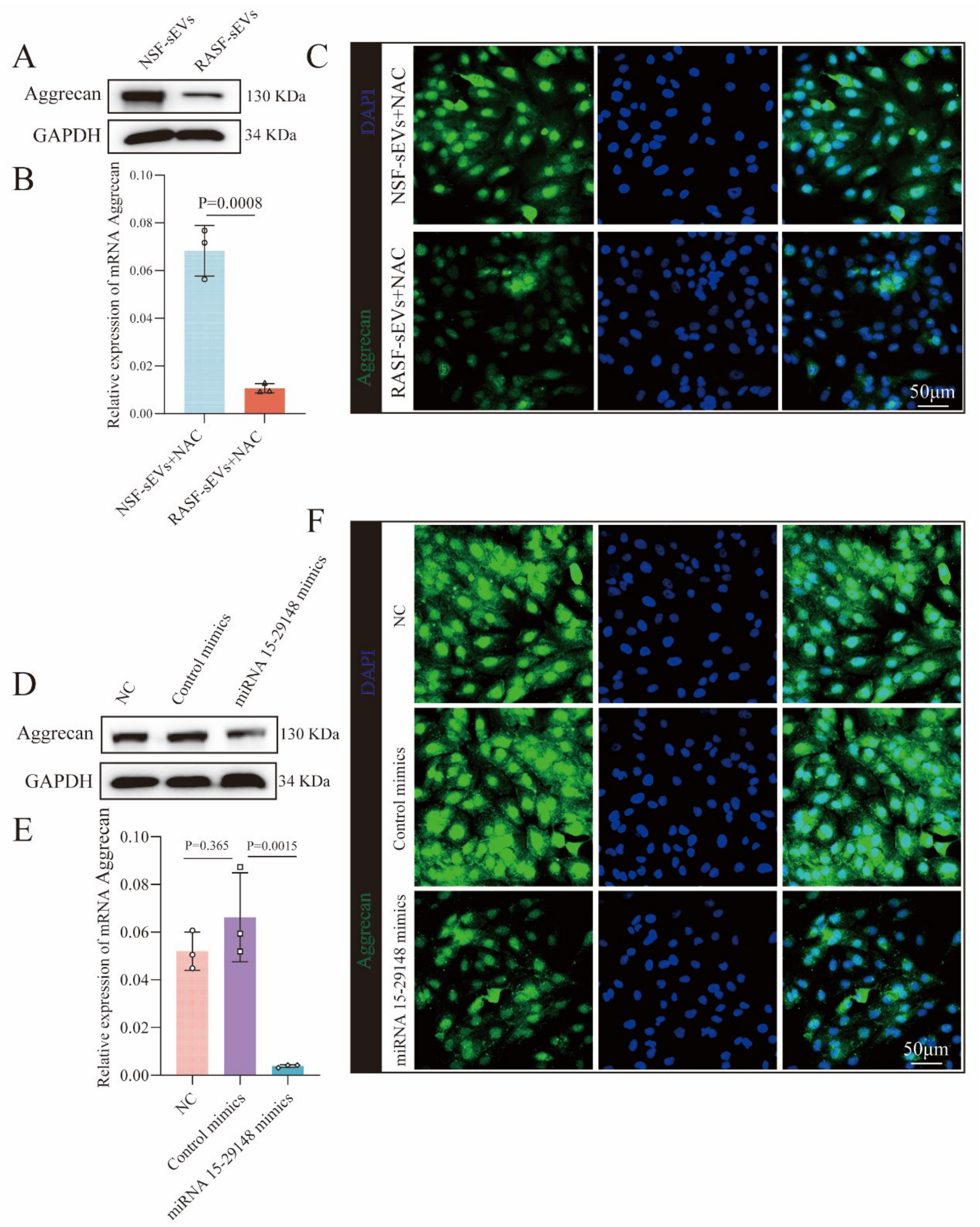

Supplemental Figure 4: aggrecan expression was down-regulated by RASF-sEVs and miRNA 15-29148（A-C）After NSF-sEVs or RASF-sEVs stimulated NAC for 48 h, WB (A) and immunofluorescence (C) were used to detect the relative expression level of aggrecan protein, and QPCR (B) was used to detect the relative expression level of aggrecan mRNA. (D-F) The relative expression level of aggrecan protein was detected by WB (D) and immunofluorescence (F), and the relative expression level of aggrecan mRNA was detected by QPCR (E) 48 h after miRNA 15-29148 or its control was transfected into NAC. (A,C,D,F) A representative image of three biologically independent samples from each group is shown. (B,E) Data are expressed as mean ± SD (n = 3 independent experiments). The p-value was calculated by one-way ANOVA.

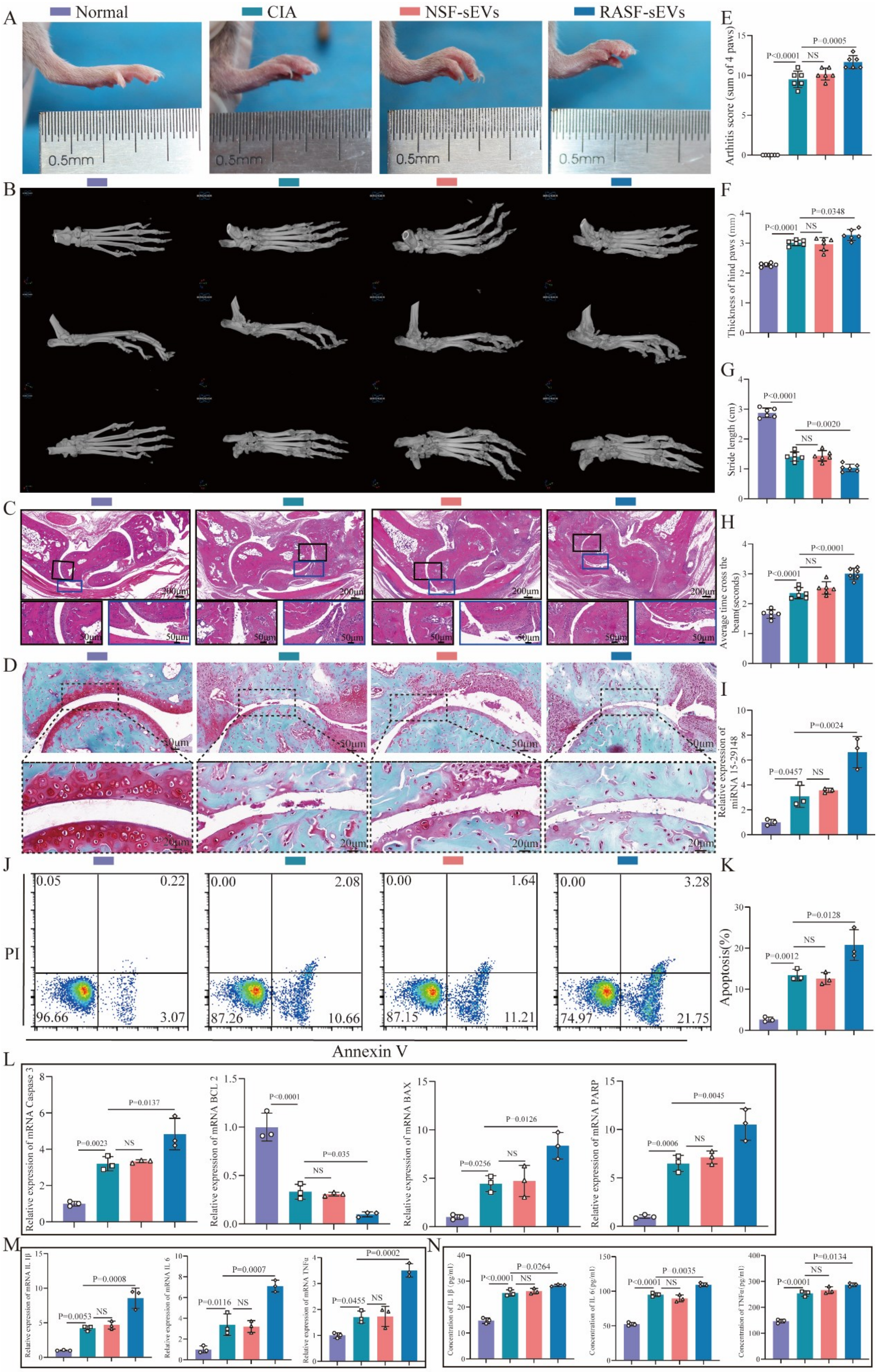

Supplemental Figure 5: RASF-sEVs promote the progression of rheumatoid

arthritis in mice

(A)Severity of soft tissue swelling and bone erosion in the paws of mice evaluated by macroscopic observation. (B)Representative microCT images of a mouse foot paw.
(C)Histological changes in the ankle joint analyzed by H&E staining. Scale: 200 μm; cartilage, black area (lower left, scale: 50 μm); synovial tissue, blue area (lower right, scale: 50 μm).(D)Articular cartilage of the ankle identified by Safranin O/Fast Green staining. Scale: 50 μm (upper panel), 20 μm (lower panel).(E)clinical scores of CIA mice.(n = 6) (F)Hind paw thickness.(n = 6) (G)Step length measured by hind paw distance.(n = 6) (H)Beam walking test in CIA mice, with the time to cross the 72 cm beam recorded.(n = 6) (I)Relative mRNA expression of miRNA 15-29148in cartilage measured by qPCR.（J-K）CIA mouse chondrocyte apoptosis analysis using flow cytometry based on Annexin V-FITC/PI staining.(n = 3).(L) Relative mRNA expression of Caspase-3 , BCL-2 , BAX , and PARP in cartilage measured by qPCR (n = 3) .(M) Relative mRNA expression levels of IL1β , IL6, and TNF-α in knee tissue detected by qPCR, with expression levels calculated as the ratio of cytokines to Actb.(n = 5). (N) Serum concentrations of proinflammatory cytokines (IL1β (L), IL6 (M), TNF-α (N)) measured by ELISA after 60 days of initial immunization. (E-I, K-N) Data are presented as mean ± SD. One-way analysis of variance and LSD test were used for statistical analysis. (A-D, J) Representative images of three biologically independent samples in each group.

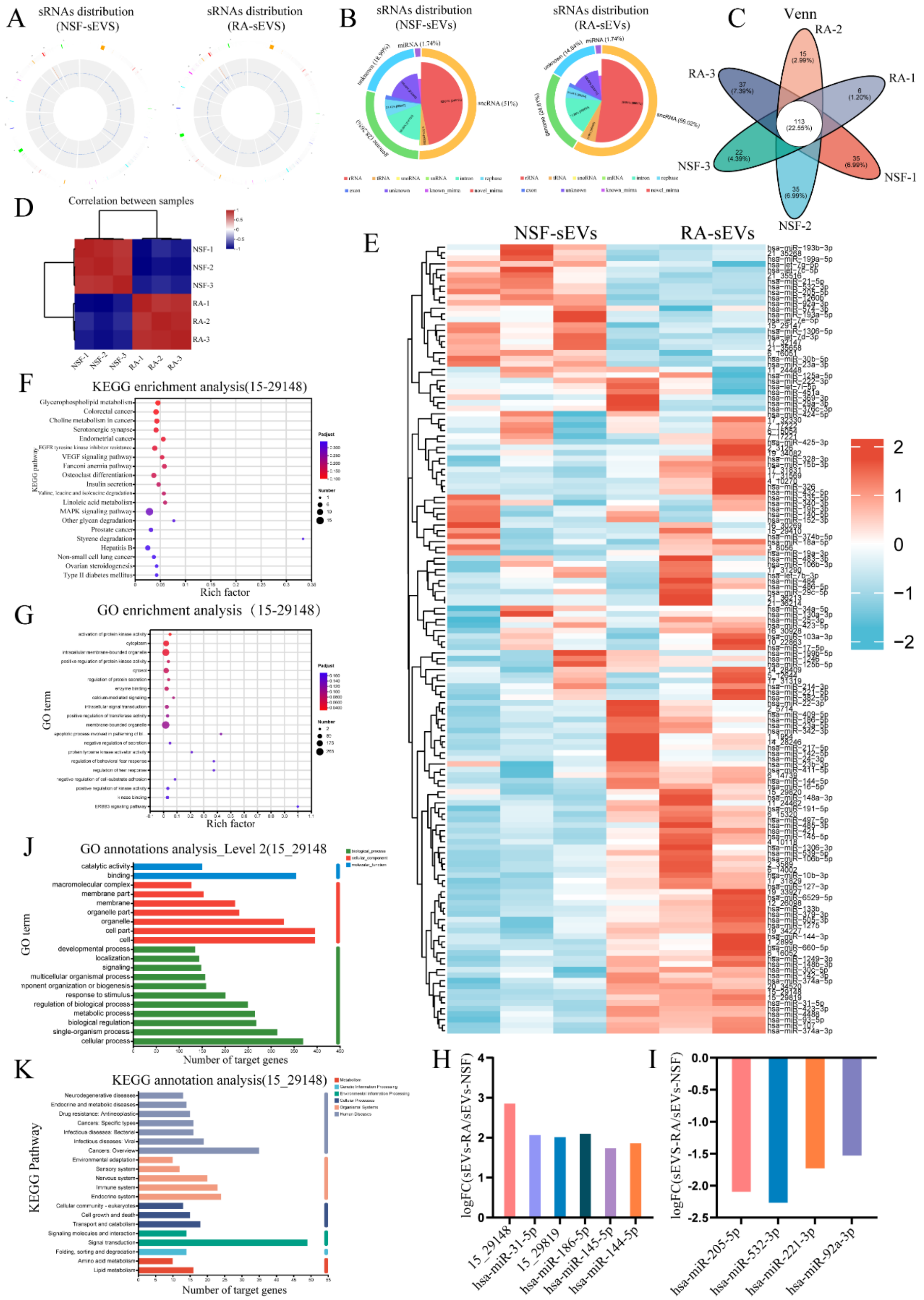

Supplemental Figure 6: miRNA Transcriptome Sequencing of NSF Exosomes and RASF Exosomes (A, B) Small RNA (sRNA) distribution of NSF-EXO and RA-EXO in the miRNA transcriptome. (C) Venn diagram showing the distribution of gene expression types in the six miRNA transcriptome samples. (D) Intersample correlation analysis of the six miRNA transcriptome samples. (E) Heat map showing differentially expressed genes in the RA-EXO group compared to the NSF-EXO group. (F) Bubble

map illustrating KEGG enrichment analysis of miRNA 15-29148. (G) Bubble map depicting GO enrichment analysis of miRNA 15-29148. (H, I) Histogram of the top 10 differentially expressed genes, with 6 genes (H) up-regulated and 4 genes (I) down-regulated in the RA-EXO group compared to the NSF-EXO group. (J) Histogram of GO annotations analysis of miRNA 15-29148. (K) Bar diagram of KEGG annotations analysis of miRNA 15-29148. A representative image of three biologically independent experiments from each group is shown in panels (B, D).

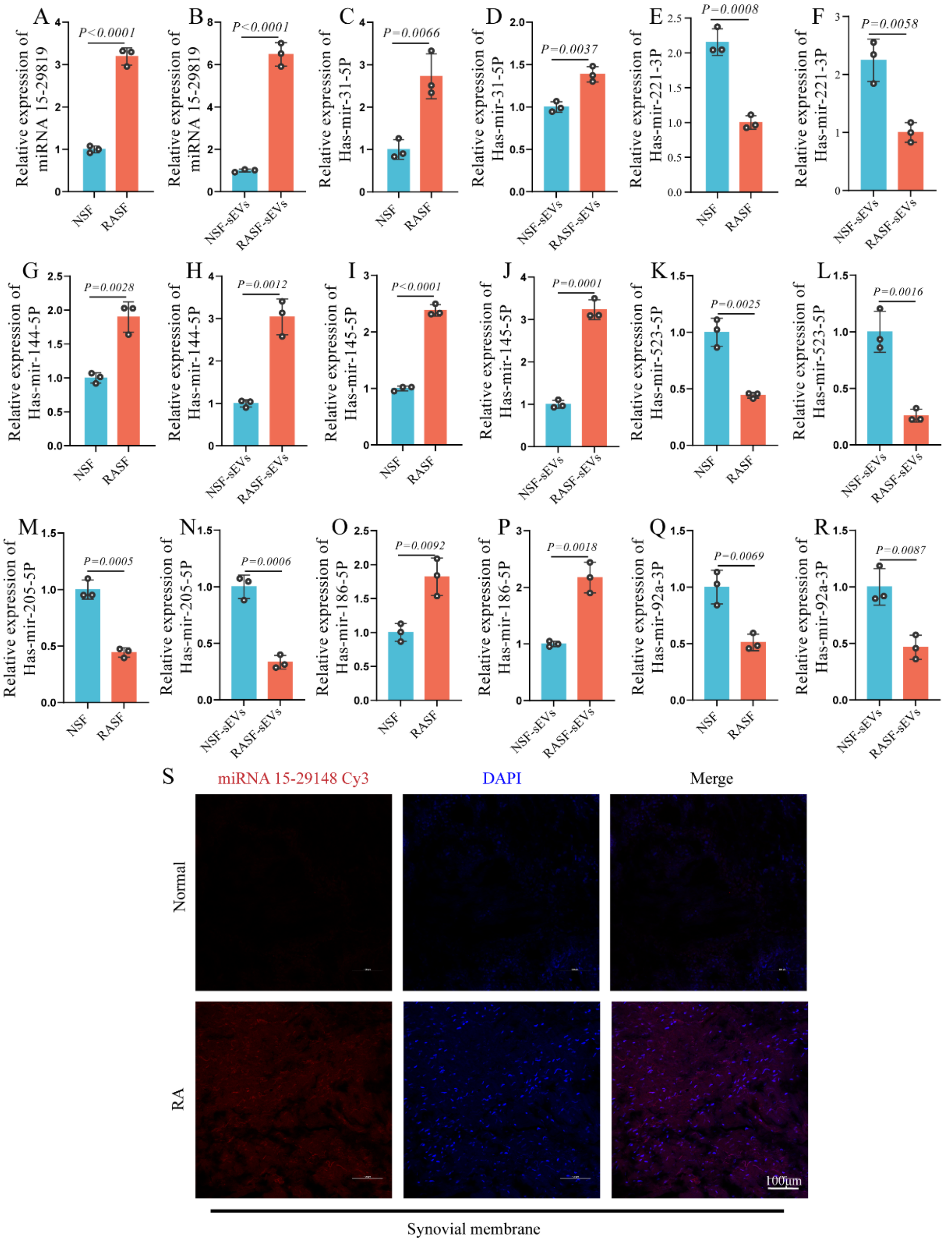

Supplemental Figure 7: miRNA Transcriptome Sequencing of Differential Genes
The expression levels of various miRNAs in human primary synovial cells (A, C, E, G, I, K, M, O, Q) and their secreted exosomes (B, D, F, H, J, L, N, P, R) were detected by RT-qPCR. (A, B) Expression level of miRNA 15-29819. (C, D) Expression level of hsa-miR-31-5p. (E, F) Expression level of hsa-miR-221-3p. (G, H) Expression level of hsa-miR-144-5p. (I, J) Expression level of hsa-miR-145-5p. (K, L) Expression level of hsa-miR-532-3p. (M, N) Expression level of hsa-miR-205-5p. (O, P) Expression level of hsa-miR-186-5p. (Q, R) Expression level of miR-92a-3p. (S) FISH analysis of RA synovial tissues demonstrating increased levels of miRNA 15-29148. Nuclei are stained blue (DAPI), and miRNA 15-29148 is stained red (Cy3). Scale bar: 100 μm. Data are

presented as mean value ± SD (n = 3 independent experiments). Student's t-test was used to calculate p-values. A representative image of three biologically independent experiments from each group is shown in panel (S).

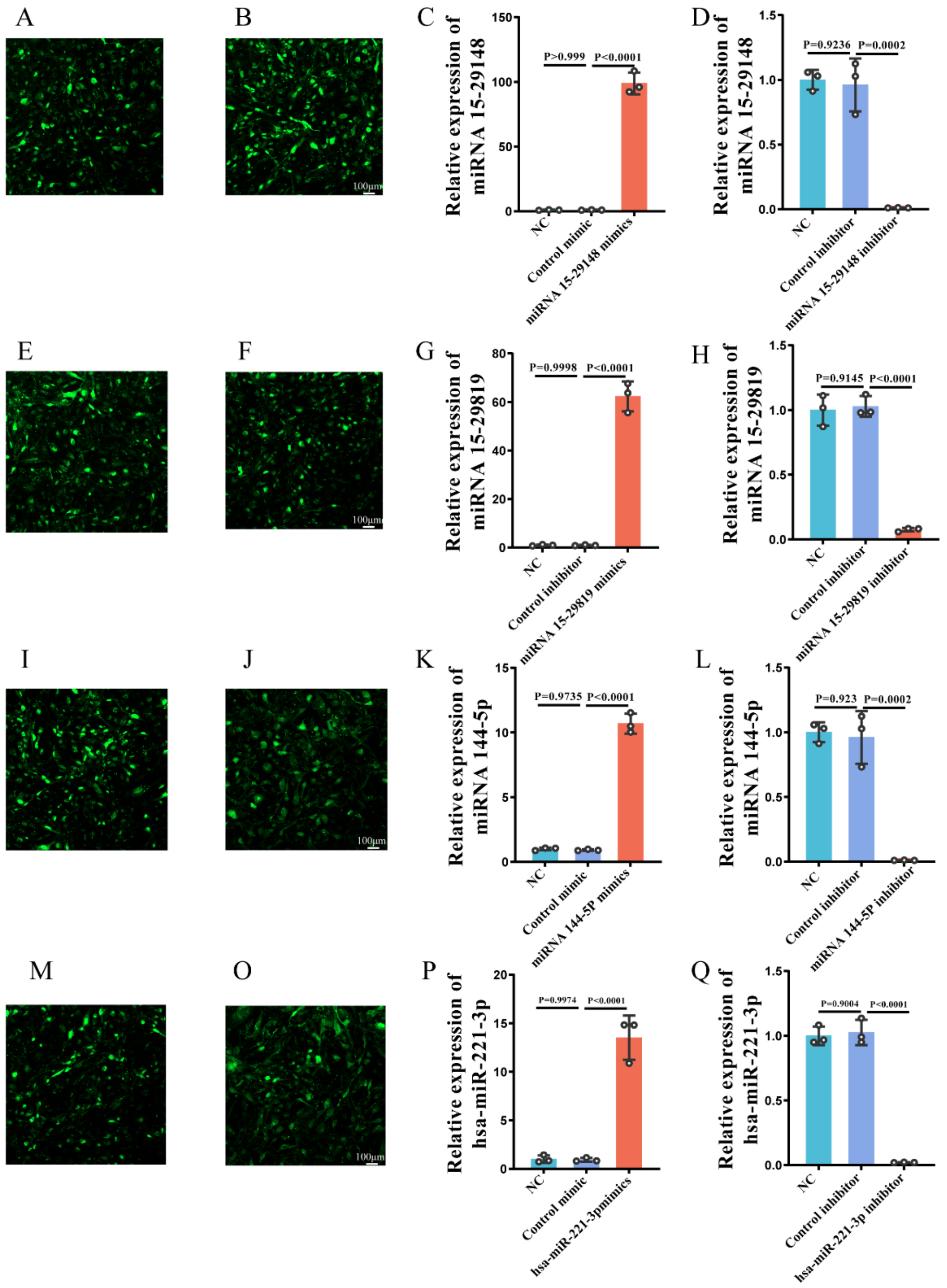

Supplemental Figure 8: Transfection Validation of miRNA in Human Primary Chondrocytes

After transfecting miRNA mimics and inhibitors in human primary chondrocytes (NCR and RACR), the expression levels were validated using fluorescence microscopy and RT-qPCR. (A, B) Transfection of miRNA 15-29148 mimics in NCR and inhibitors in RACR. (A) Fluorescence microscopy for miRNA 15-29148 mimics. (B) Fluorescence microscopy for miRNA 15-29148 inhibitors. (C) RT-qPCR validation for miRNA 15-29148 mimics. (D) RT-qPCR validation for miRNA 15-29148 inhibitors. (E, F) Transfection of miRNA 15-29819 mimics in NCR and inhibitors in RACR. (E)

Fluorescence microscopy for miRNA 15-29819 mimics. (F) Fluorescence microscopy for miRNA 15-29819 inhibitors. (G) RT-qPCR validation for miRNA 15-29819 mimics. (H) RT-qPCR validation for miRNA 15-29819 inhibitors. (I, J) Transfection of hsa-miR-144-5p mimics in NCR and inhibitors in RACR. (I) Fluorescence microscopy for hsa-miR-144-5p mimics. (J) Fluorescence microscopy for hsa-miR-144-5p inhibitors. (K) RT-qPCR validation for hsa-miR-144-5p mimics. (L) RT-qPCR validation for hsa-miR-144-5p inhibitors. (M, O) Transfection of hsa-miR-221-3p mimics in NCR and inhibitors in RACR. (M) Fluorescence microscopy for hsa-miR-221-3p mimics. (O) Fluorescence microscopy for hsa-miR-221-3p inhibitors. (P) RT-qPCR validation for hsa-miR-221-3p mimics. (Q) RT-qPCR validation for hsa-miR-221-3p inhibitors. Scale bars: 100 μm. A representative image of three biologically independent experiments from each group is shown in panels (A, B), (E, F), (I, J), and (M, O). Data are presented as mean value ± SD (n = 3 independent experiments). One-way ANOVA was used to calculate p-values in panels (C, D), (G, H), (K, L), and (P, Q).

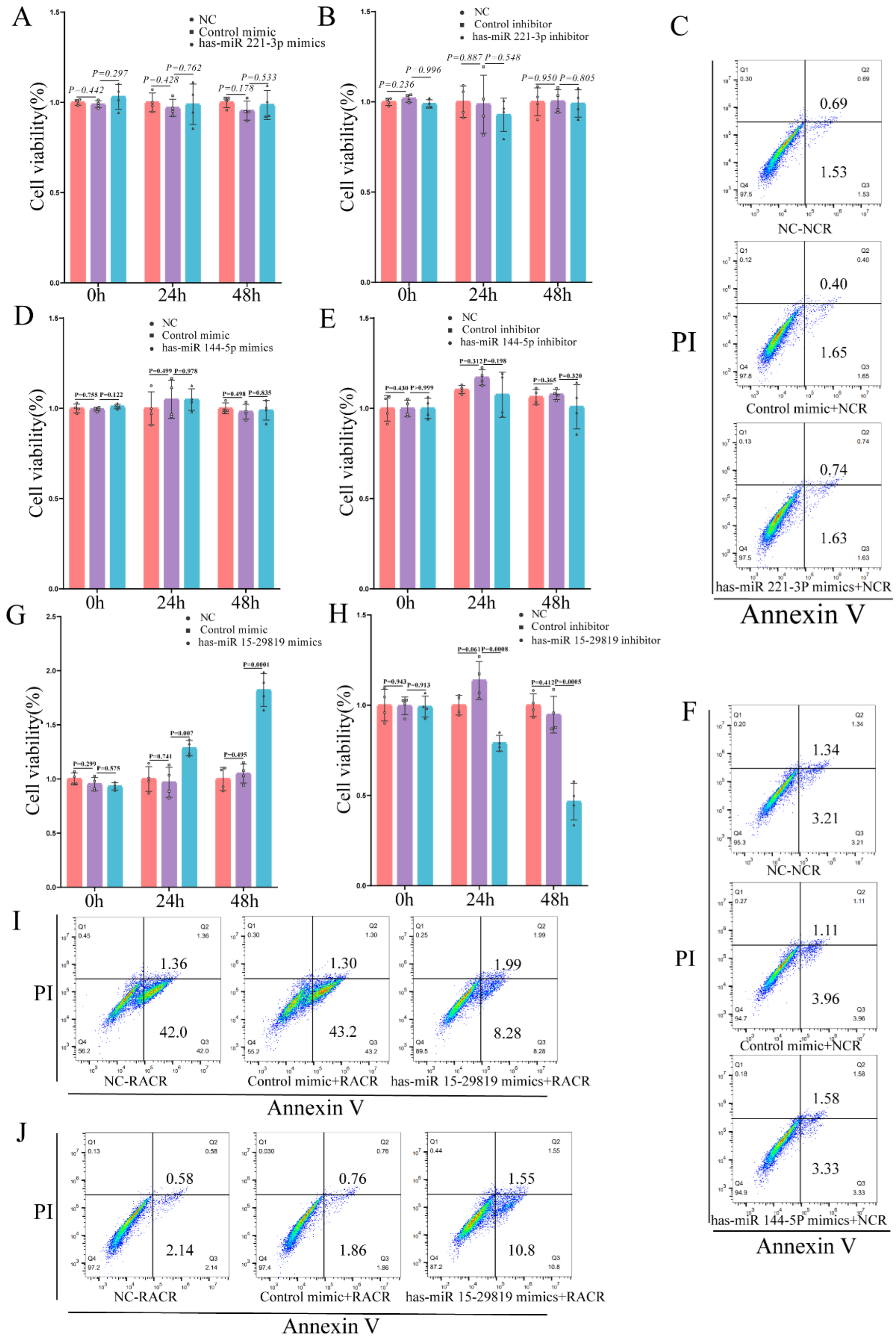

Supplemental Figure 9: Efficacy of Apoptosis-Related miRNAs in Regulating Apoptosis of Human Primary Chondrocytes (A-C) hsa-miR-221-3p does not regulate apoptosis of human primary chondrocytes. (A) Cell viability changes detected by CCK8 after transfection of hsa-miR-221-3p mimics in NCR. (B) Cell viability detected by CCK8 after transfection with hsa-miR-221-3p inhibitor in RACR. (C) Percentage of apoptosis detected by flow cytometry after transfection of hsa-miR-221-

3p mimics in NCR. (D-F) hsa-miR-144-5p does not regulate apoptosis of human primary chondrocytes. (D) Cell viability changes detected by CCK8 after transfection of hsa-miR-144-5p mimics in NCR. (E) Cell viability detected by CCK8 after transfection with hsa-miR-144-5p inhibitor in RACR. (F) Percentage of apoptosis detected by flow cytometry after transfection of hsa-miR-144-5p mimics in NCR. (G-J) miRNA 15-29819 inhibits apoptosis of human primary chondrocytes. (G) Cell viability changes detected by CCK8 after transfection of miRNA 15-29819 mimics in NCR. (H) Cell viability detected by CCK8 after transfection with miRNA 15-29819 inhibitor in RACR. (I) Percentage of apoptosis detected by flow cytometry after transfection of miRNA 15-29819 mimics in NCR. (J) Percentage of apoptosis detected by flow cytometry after transfection with miRNA 15-29819 inhibitor in RACR. Data are presented as mean value ± SD (n = 5 independent experiments). One-way ANOVA was used to calculate p-values in (A, B), (D, E), (G, H). Representative images of three biologically independent samples in each group are displayed in panels (C), (F), (I), and (J).

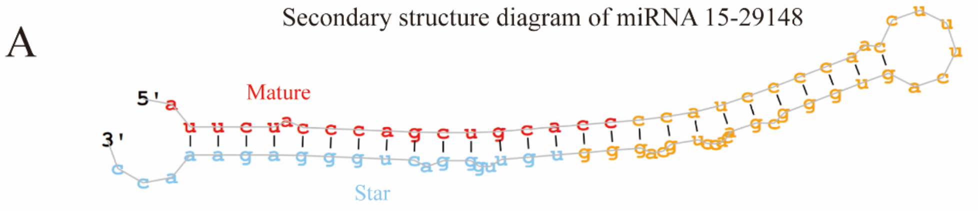

B

miRNA 15-29148 Basic information

miRNA name	miRNA seq	miRNA len	pre-miRNA name	pre-miRNA seq	pre-miRNA len	pre position
15-29148	auucuac<br>ccagcug<br>cacc	18	15-29148	auucuacccagcu<br>gcaccccauccccca<br>accuuucaguggg<br>cgacaccugcagg<br>guguugggacugg<br>gagaaacc	74	15(+):3<br>019076<br>5-<br>301908<br>39

Supplemental Figure 10: Basic information of miRNA 15-29148 (A) Schematic diagram of miRNA 15-29148 secondary structure; (B) Basic information and sequence of miRNA 15-29148.

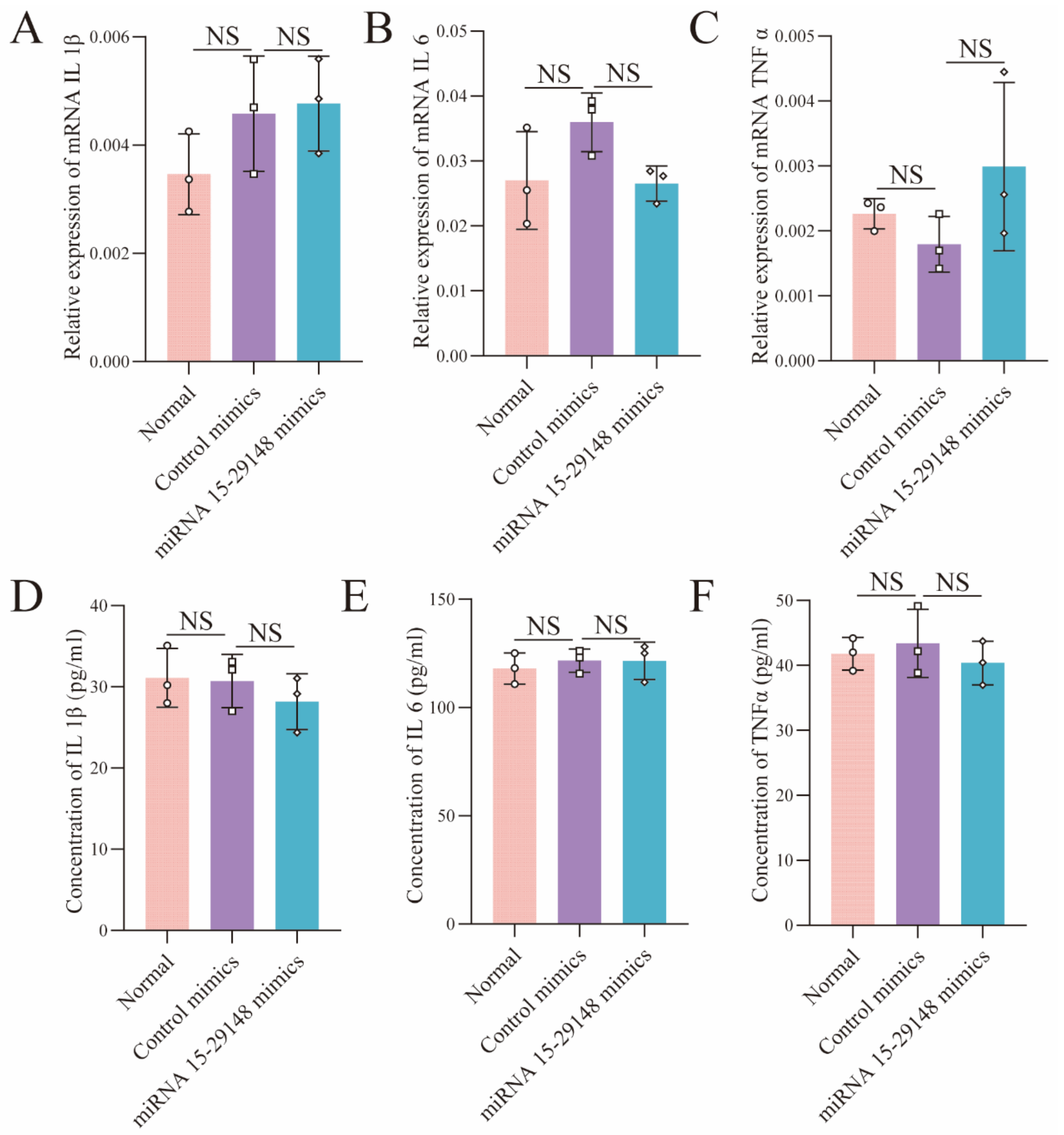

Supplemental Figure 11: Regulation of chondrocyte inflammation levels by miRNA15-29148. (A-C)Relative mRNA expression levels of IL 1β, IL 6, and TNF α in NCR measured by qPCR after treatment with miRNA 15-29148 mimics. (D-F)Relative expression levels of IL 1β, IL 6, and TNF α in NCR measured by ELISA after treatment with miRNA 15-29148 mimics. Data are expressed as mean ± SD (n = 3 independent experiments). The p-value was calculated by one-way ANOVA.

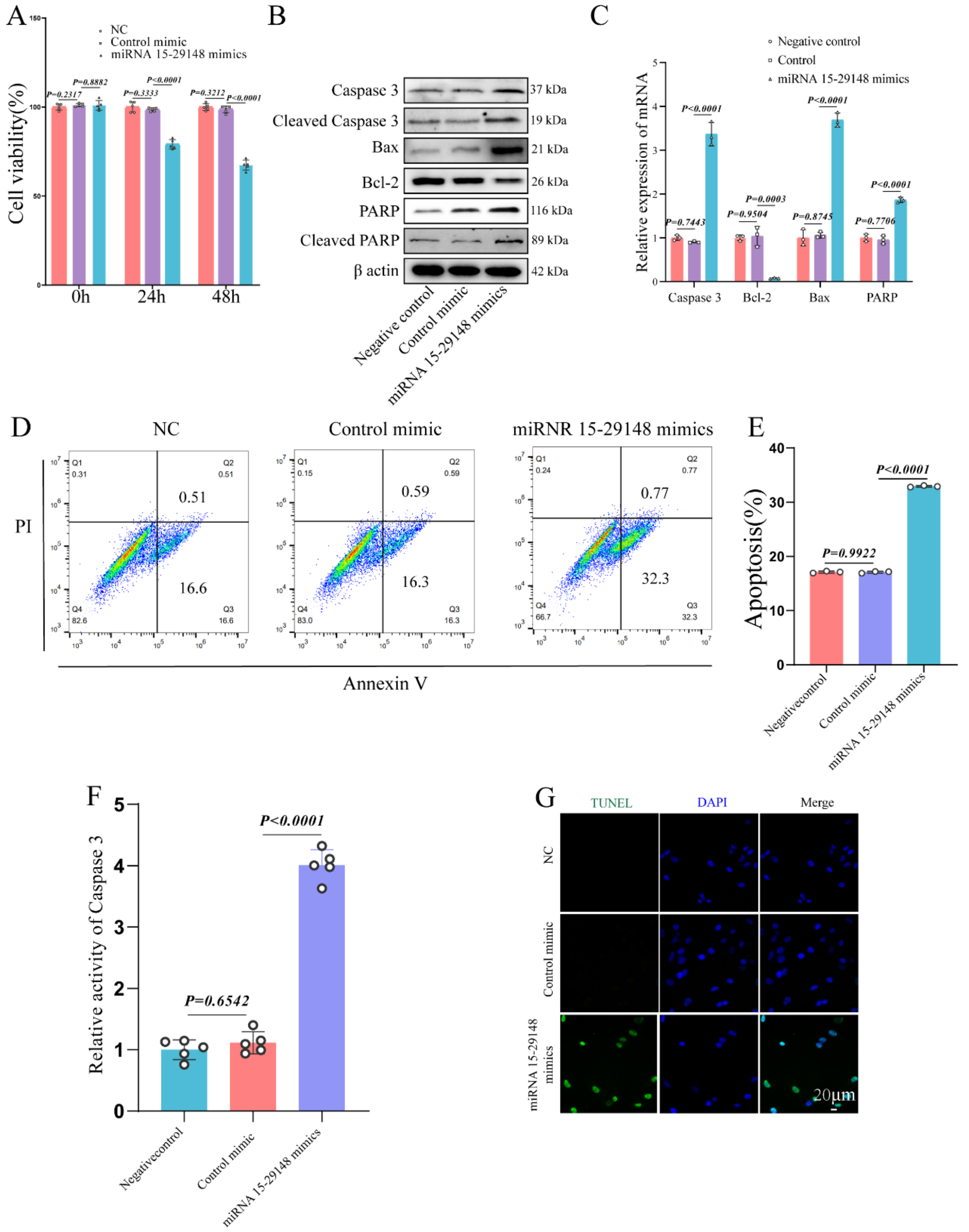

Supplemental Figure 12: miRNA 15-29148 Further Promotes Apoptosis of Primary Chondrocytes in RA Patients (A) Cell viability of RACR treated with miRNA 15-29148 mimics for different durations analyzed by CCK8 assay. Data are presented as mean value ± SD (n = 5 independent experiments). One-way ANOVA was used to calculate p values. (B) Western blot analysis of Caspase-3, Cleaved Caspase-3, BAX, BCL-2, PARP, and Cleaved PARP expression levels in RACR treated with miRNA 15-29148 mimics for 48 hours (n = 3 independent experiments). (C) Relative mRNA expression levels of Caspase-3, BAX, BCL-2, and PARP in RACR measured by qPCR after treatment with miRNA 15-29148 mimics. Data are presented as mean

value ± SD (n = 3 independent experiments). One-way ANOVA was used to calculate p values. (D, E) Flow cytometry analysis of apoptosis in RACR based on Annexin V-FITC/PI staining after treatment with miRNA 15-29148 mimics. Data are presented as mean value ± SD (n = 3 independent experiments). One-way ANOVA was used to calculate p values. (F) Caspase-3 activity in RACR detected after 48 hours of culture with miRNA 15-29148 mimics. Data are presented as mean value ± SD (n = 5 independent experiments). One-way ANOVA was used to calculate p values. (G) TUNEL staining of RACR cultured with miRNA 15-29148 mimics for 48 hours. Nuclei are stained blue (DAPI); TUNEL-positive cells are stained green. Scale bar: 20 μm. A representative image of 3 biologically independent experiments from each group is shown.

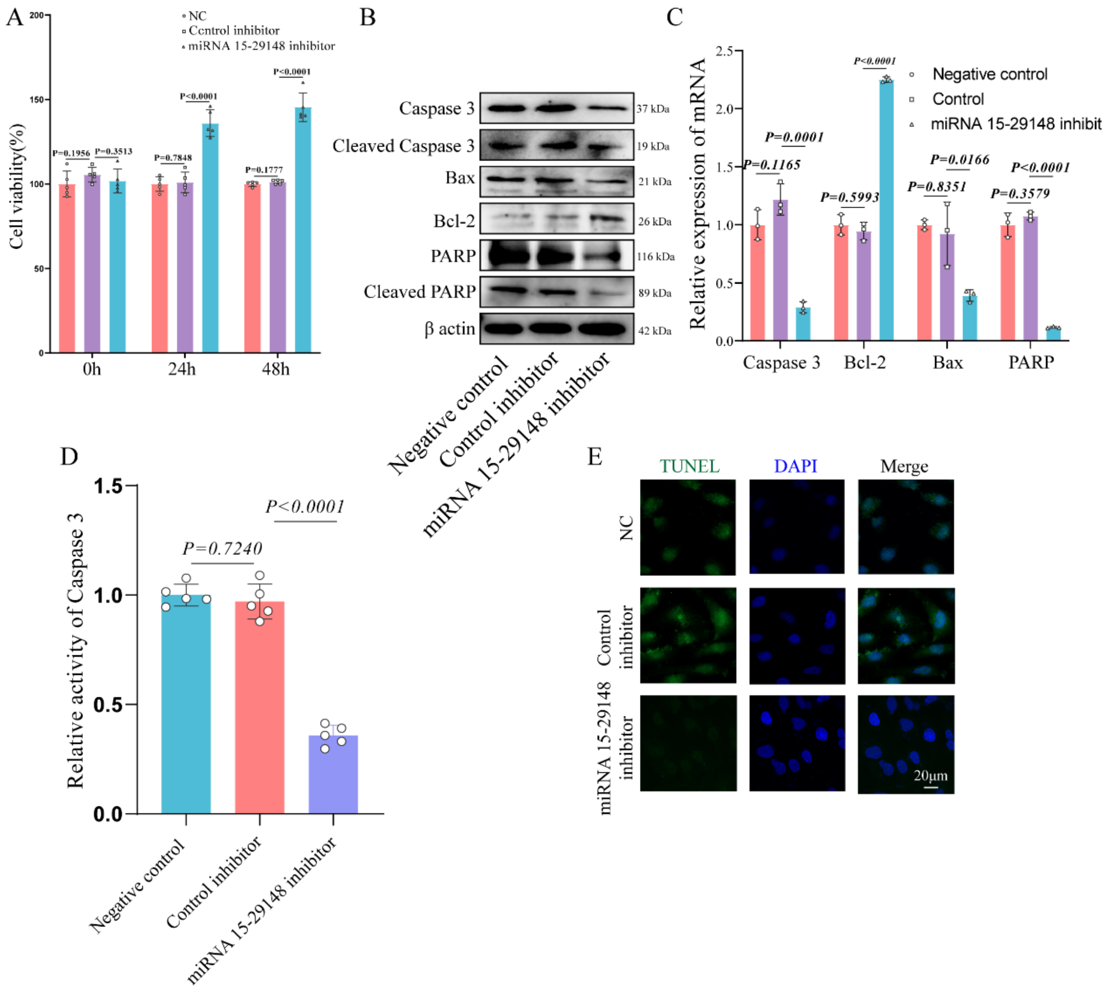

Supplemental Figure 13: miRNA 15-29148 Further Inhibits Apoptosis of Normal Primary Chondrocytes (A) Cell viability of NCR treated with miRNA 15-29148 inhibitor for different durations analyzed by CCK8 assay. Data are presented as mean value ± SD (n = 5 independent experiments). One-way ANOVA was used to calculate p values. (B) Western blot analysis of Caspase-3, Cleaved Caspase-3, BAX, BCL-2, PARP, and Cleaved PARP expression levels in NCR treated with miRNA 15-29148 inhibitor for 48 hours (n = 3 independent experiments). (C) Relative mRNA expression levels of Caspase-3, BAX, BCL-2, and PARP in NCR measured by qPCR after treatment with miRNA 15-29148 inhibitor. Data are presented as mean value ± SD (n = 3 independent experiments). One-way ANOVA was used to calculate p values. (D) Caspase-3 activity in NCR detected after 48 hours of culture with miRNA 15-29148 inhibitor. Data are presented as mean value ± SD (n = 5 independent experiments). One-way ANOVA was used to calculate p values. (E) TUNEL staining of NCR cultured with miRNA 15-29148 inhibitor for 48 hours. Nuclei are stained blue (DAPI); TUNEL-positive cells are stained green. Scale bar: 20 μm. A representative image of 3 biologically independent experiments from each group is shown.

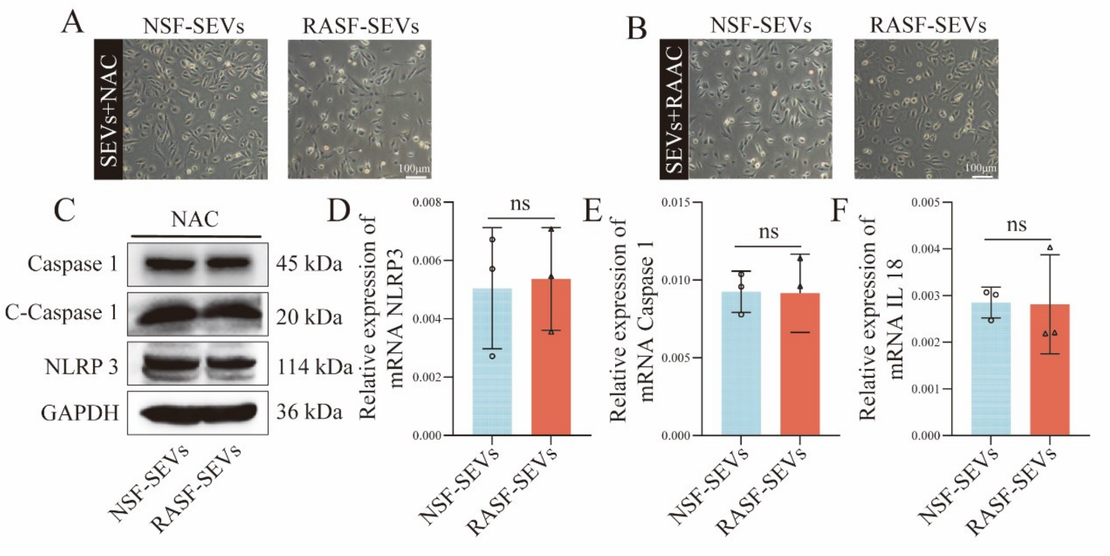

Supplemental Figure 14: RASF-sEVs has no significant effect on the level of AC pyrodeath (A-B) Cell morphology after NSF-sEVs or RASF-sEVs stimulation of NAC (A) or RAAC (B) for 48 h. (C) WB was used to detect the expression level of pyrodeath related proteins; (D-F) QPCR was used to detect the relative expression level of pyroptosis related mRNA. (A-C) Representative images of three biologically independent samples in each group. (D-F) Data were expressed as mean±SD (n = 3 independent experiments). P-values were calculated using one-way analysis of variance.

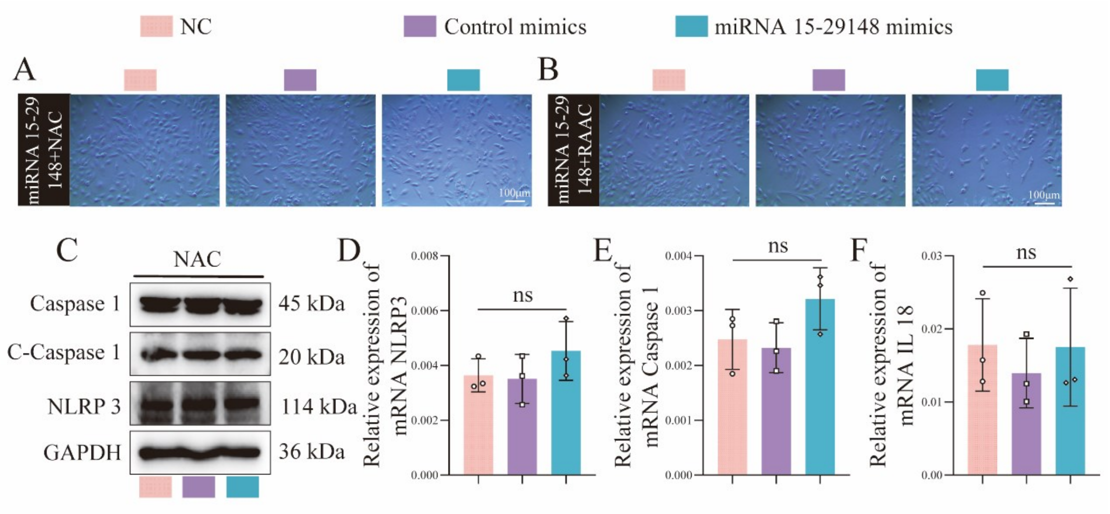

Supplemental Figure 15: miRNA 15-29148 has no significant effect on the level of AC pyrodeath. (A-B) Cell morphology after miRNA 15-29148 or its control was transfected into NAC (A) or RAAC (B) for 48 h. (C) WB was used to detect the expression level of pyrodeath related proteins; (D-F) QPCR was used to detect the relative expression level of pyroptosis related mRNA. (A-C) Representative images of three biologically independent samples in each group. (D-F) Data were expressed as mean±SD (n = 3 independent experiments). P-values were calculated using one-way analysis of variance.

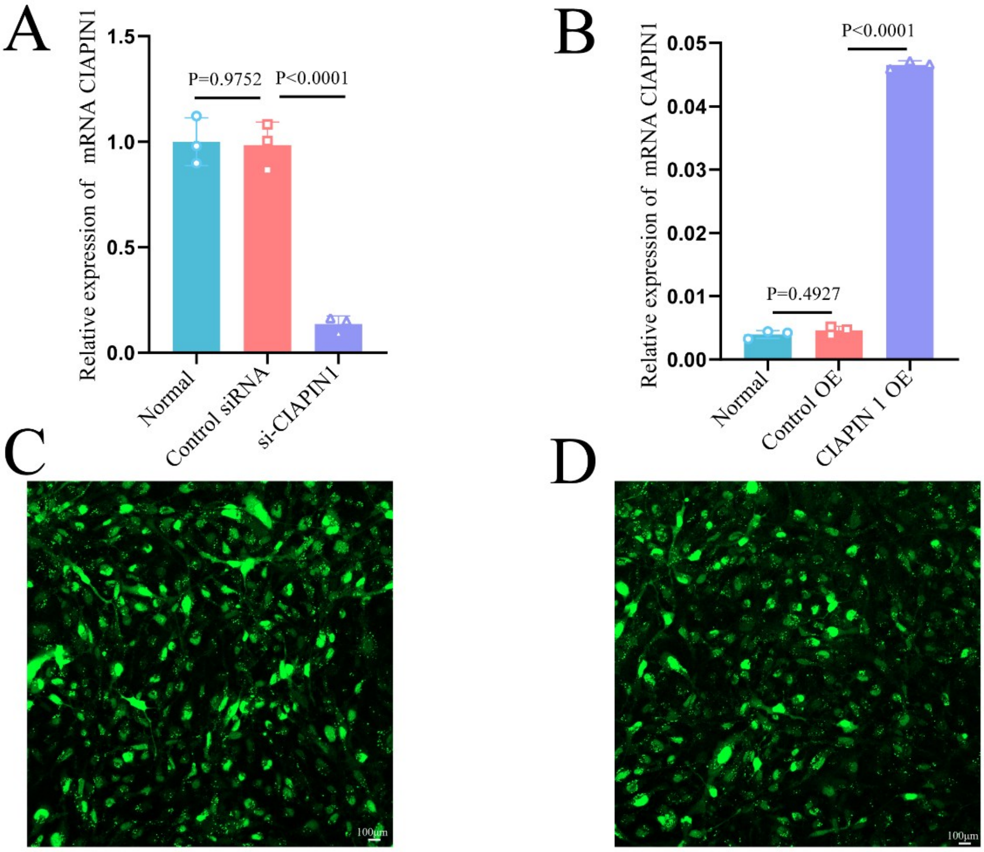

Supplemental Figure 16: Transfection Validation in Human Primary Chondrocytes After transfecting si-CIAPIN 1 in normal human primary chondrocytes (NCR): (A) RT-QPCR was performed to confirm the expression level of CIAPIN 1. (C) The expression level of CIAPIN 1 was verified by fluorescence microscopy. After transfection with CIAPIN 1 overexpression (OE) in rheumatoid arthritis human primary chondrocytes (RACR): (B) RT-QPCR was performed to verify the expression level of CIAPIN 1. (D) Fluorescence microscopy was used to confirm the expression level of CIAPIN 1. (C,D)A representative image of three biologically independent samples from each group is shown.

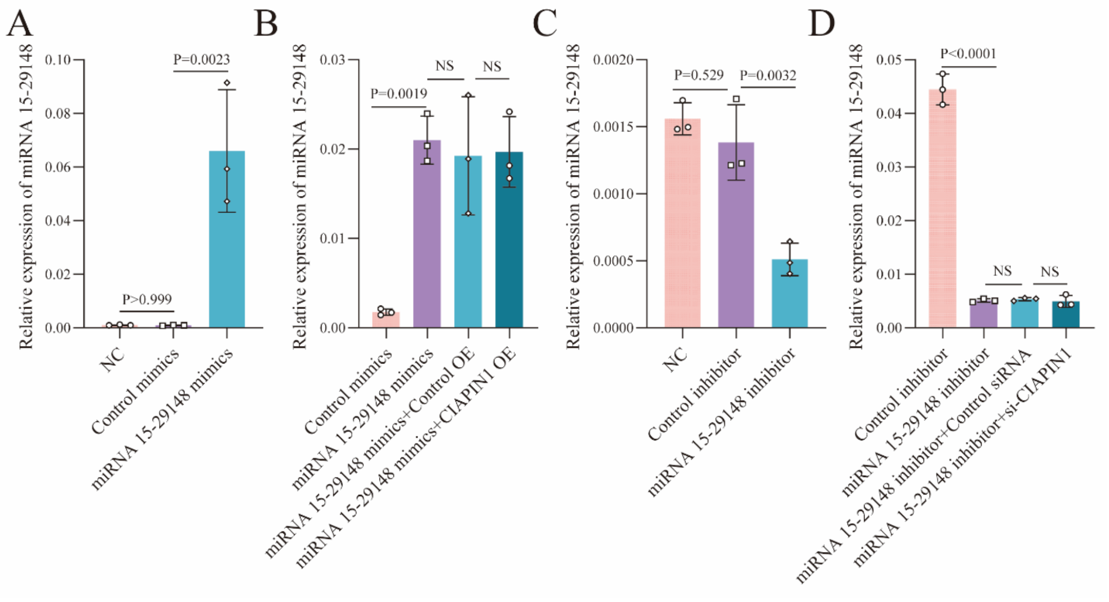

Supplemental Figure 17: Expression level of miRNA 15-29148 after transfection with miRNA 15-29148 mimics/inhibitor (A-B) Expression levels of miRNA 15-29148 in NAC after miRNA 15-29148 mimics transfection; (C-D) Expression levels of miRNA 15-29148 in NAC after miRNA 15-29148 inhibitor transfection.

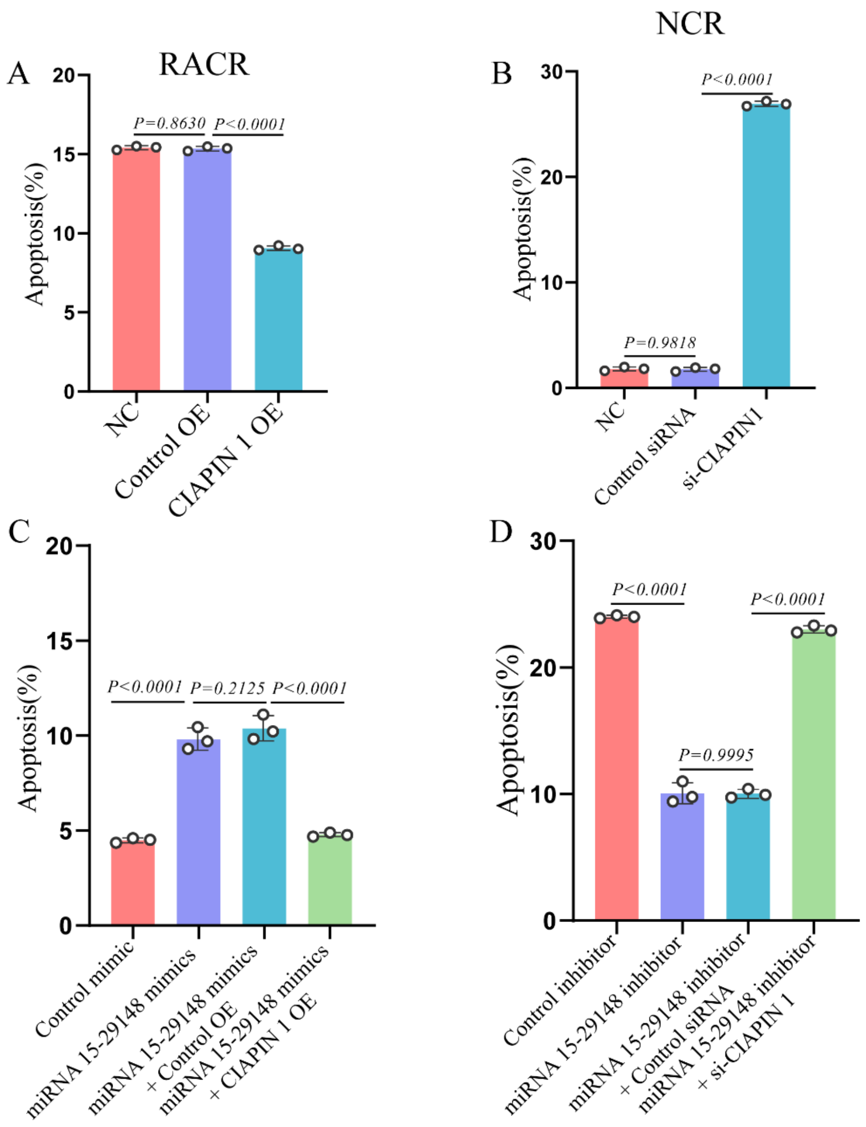

Supplemental Figure 18: Flow Cytometry Analysis of Apoptosis Flow cytometry based on Annexin V-FITC/PI staining was used to analyze: (A) The apoptosis ratio of rheumatoid arthritis human primary chondrocytes (RACR) after transfection with CIAPIN1 overexpression (OE). (B) The apoptosis ratio of normal human primary chondrocytes (NCR) after transfection with si-CIAPIN1. (C) The apoptosis ratio of NCR after co-transfection with miRNA 15-29148 inhibitor and si-CIAPIN1. (D) The apoptosis ratio of NCR after co-transfection with miRNA 15-29148 mimics and CIAPIN1 OE. Data are expressed as mean ± SD (n = 3 independent experiments). The p-value was calculated by one-way ANOVA.

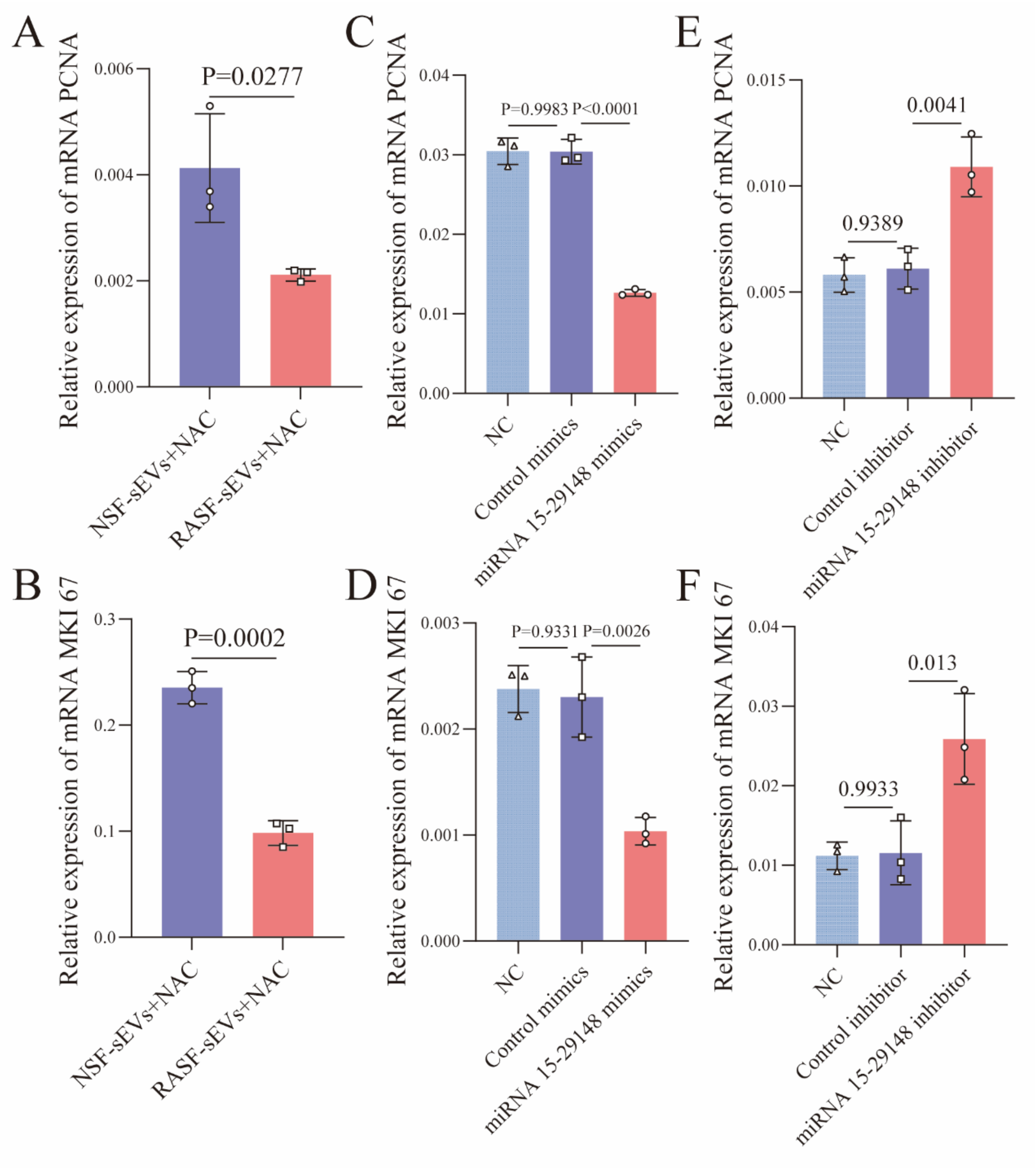

Supplemental Figure 19: RASF-sEVs and miRNA 15-29148 inhibit chondrocyte proliferation. (A-B) mRNA expression levels of NAC proliferation markers KI67 and PCNA were detected by QPCR after 48 h of RASF-sEVs or its control. (C-D) QPCR was used to detect the mRNA expression levels of NAC proliferation markers KI67 and PCNA 48 h after miRNA 15-29148 mimics transfection. (E-F) QPCR was used to detect mRNA expression levels of NAC proliferation markers KI67 and PCNA 48 h after transfection with miRNA 15-29148 inhibitor. Data are expressed as mean ± SD (n = 3 independent experiments). The p-value was calculated by one-way ANOVA.

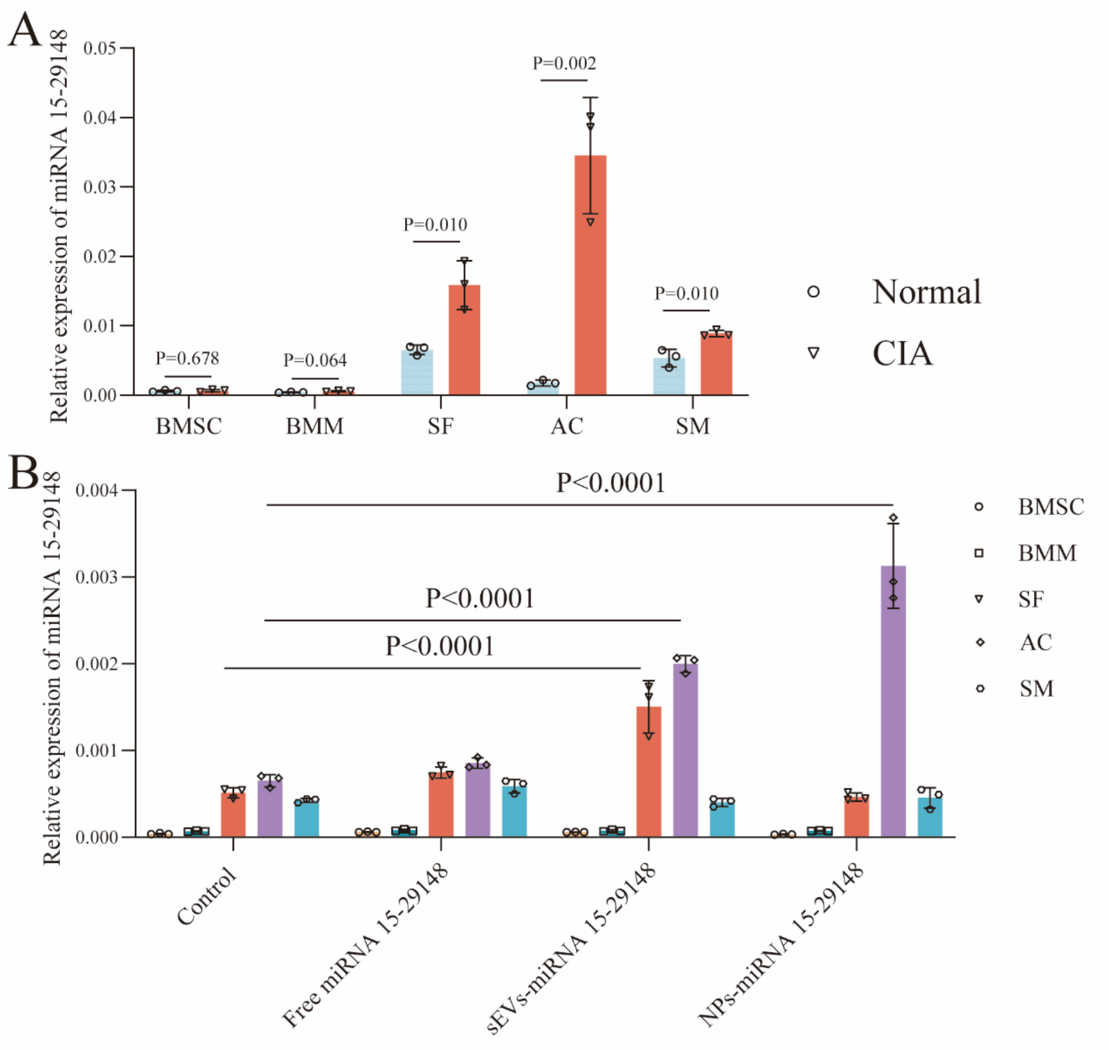

Supplemental Figure 20: miRNA 15-29148 specific expression. (A) Relative expression of miRNA 15-29148 in bone marrow mesenchymal stem cells (BMSC), bone marrow macrophages (BMM), synovial fibroblasts (SF), articular chondrocytes (AC), and synovial macrophages (SM) in the joints of CIA or control mice, respectively, was examined using QPCR. (B) Relative expression of miRNA 15-29148 in BMSC, BMM, SF, AC, SM in the joints 48 hours after intra-articular injection of free miRNA 15-29148, sEVs-miRNA 15-29148 or NPs-miRNA 15-29148 was detected using QPCR. Data are presented as the mean ± s.d., with biologically individual data points shown. P values were determined by ordinary one-way ANOVA test with Tukey's multiple comparisons.

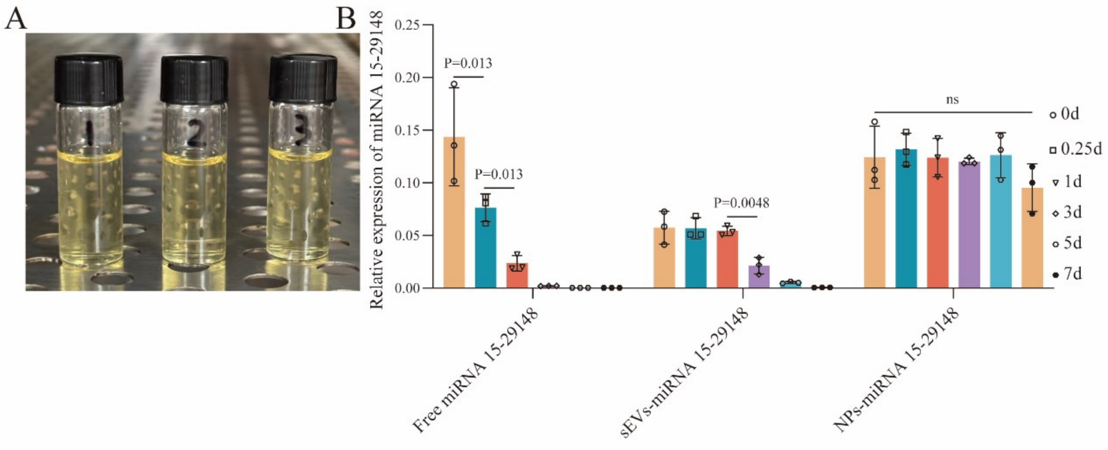

Supplemental Figure 21: Stability of miRNA 15-29148

(A) Photos of different mirnas 15-29148 dispersed in joint fluid after incubation at 37℃ for different time. (B) The expression levels of miRNA 15-29148 were detected by QPCR after incubation at 37℃ for different time. Data are presented as mean ± SD. One-way analysis of variance and LSD test were used for statistical analysis.

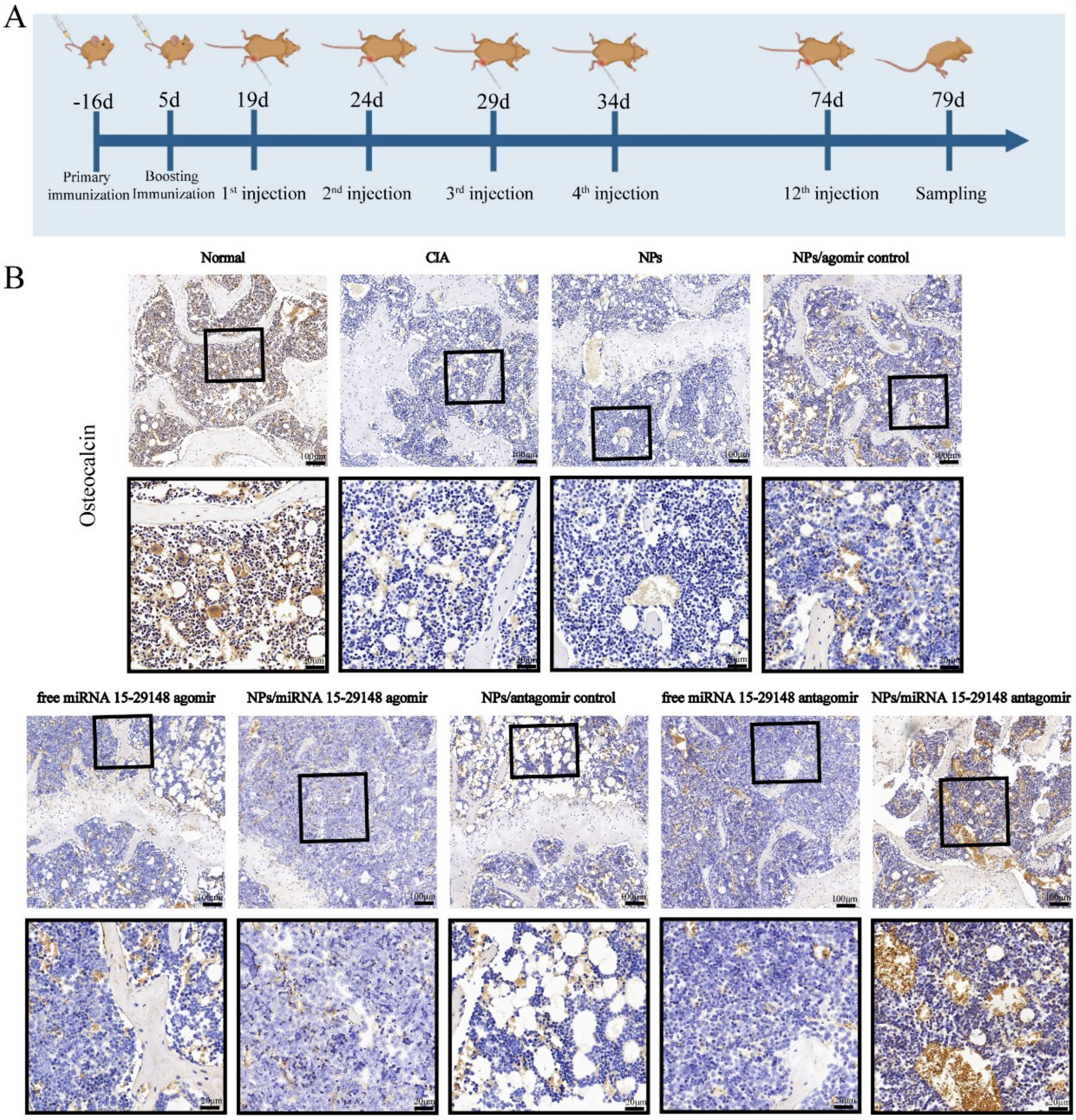

Supplemental Figure 22: tgg2-PEG2000-PAMAM6.0-Cy5.5 / miRNA 15-29148 Antagomir Alleviates Bone Erosion in CIA Mice (A) Experimental set-up overview with injections of miRNA 15-29148 agomir, miRNA 15-29148 antagomir, or their negative control NPs. (B) Immunohistochemistry detection of Osteocalcin expression in arthritic joints. Scale: 100 µm (top), 20 µm (bottom). Representative images of three biologically independent samples in each group are shown.

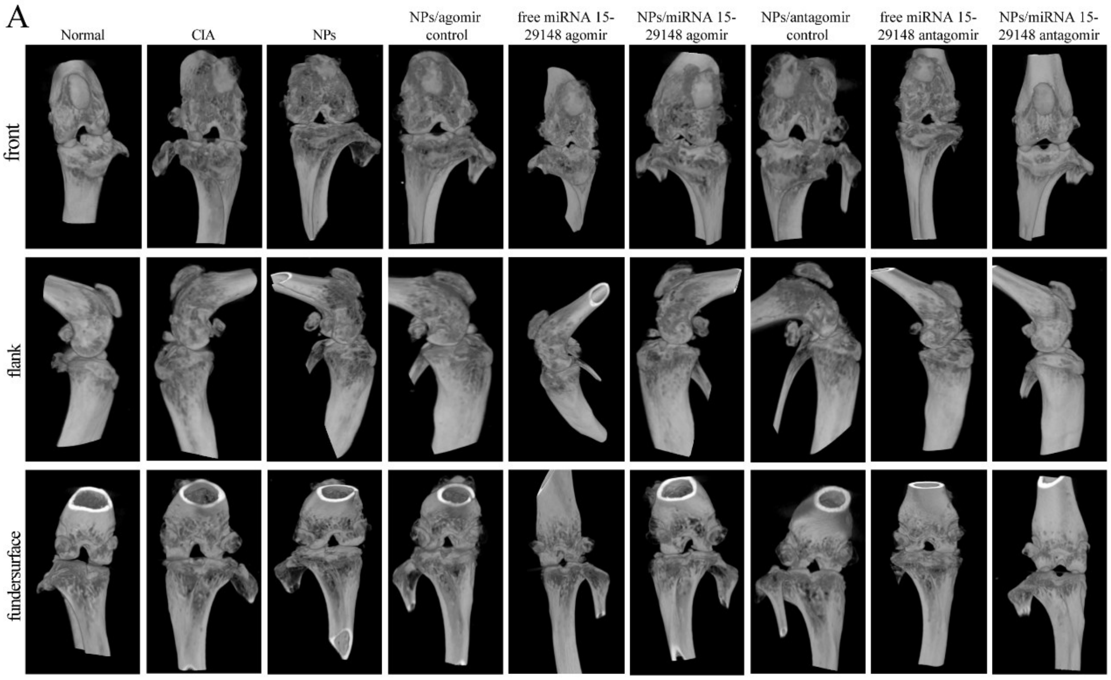

Supplemental Figure 23: (A) Representative microCT images of the knee joint 8 weeks after administration. A representative image of three biologically independent samples in each group is shown.

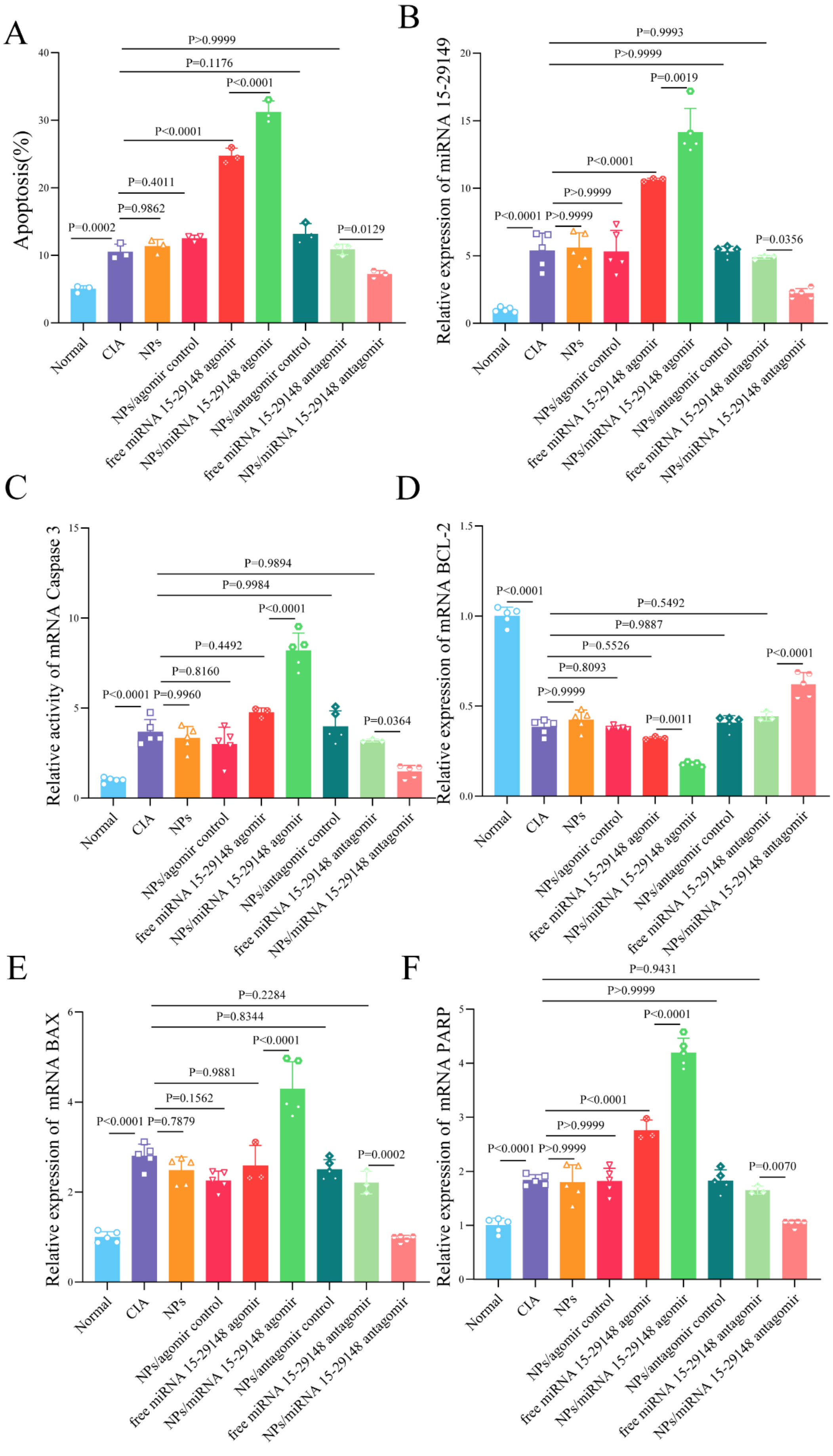
A
Apoptosis(%)
P>0.9999
P=0.1176
P<0.0001
P<0.0001
P=0.4011
P=0.9862
P=0.0002
P=0.0129
B
Relative expression of miRNA 15-29149
P=0.9993
P>0.9999
P=0.0019
P<0.0001
P>0.9999
P<0.0001
P>0.9999
P=0.0356
C
Relative activity of mRNA Caspase 3
P=0.9894
P=0.9984
P<0.0001
P=0.4492
P=0.8160
P<0.0001
P=0.9960
P=0.0364
D
Relative expression of mRNA BCL-2
P<0.0001
P=0.5492
P=0.9887
P=0.5526
P=0.8093
P>0.9999
P=0.0011
P<0.0001
E
Relative expression of mRNA BAX
P=0.2284
P=0.8344
P<0.0001
P=0.9881
P=0.1562
P<0.0001
P=0.7879
P=0.0002
F
Relative expression of mRNA PARP
P=0.9431
P>0.9999
P<0.0001
P<0.0001
P>0.9999
P<0.0001
P>0.9999
P=0.0070
Normal
CIA
NPs
NPs/agomir control
free miRNA 15-29148 agomir
NPs/miRNA 15-29148 agomir
NPs/antagomir control
free miRNA 15-29148 antagomir
NPs/miRNA 15-29148 antagomir

Supplemental Figure 24: (A) Analysis of chondrocyte apoptosis in CIA mice after 8 weeks of administration using flow cytometry based on Annexin V-FITC/PI staining. Data are presented as mean value ± SD (n = 3 independent experiments). One-way ANOVA was used to calculate p values. (B) Relative mRNA expression levels of miRNA 15-29148, (C) Caspase-3, (D) BCL-2, (E) BAX, and (F) PARP in knee tissue were detected by qPCR after 60 days of initial immunization. The expression levels were calculated as the ratio of respective cytokines to Actb. Data are expressed as mean ± SD (n = 5 independent animals). One-way analysis of variance (ANOVA) and LSD test were used for statistical analysis.

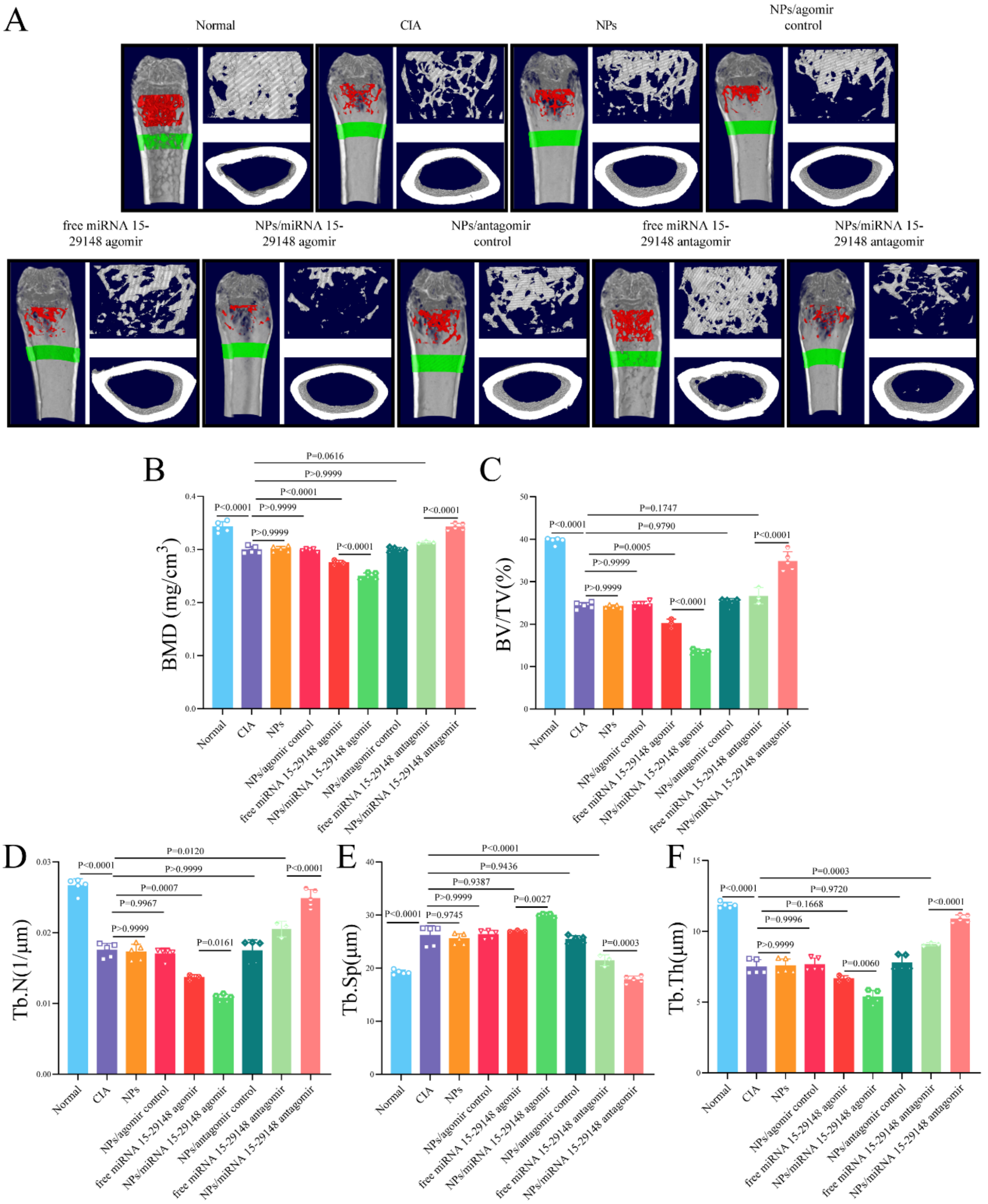

Supplemental Figure 25: Bone protection of NPs/miRNA 15-29148 antagomir nanoparticles in CIA mice. (A) Representative micro-CT images of CIA mice after 8 weeks of administration with NPs/miRNA 15-29148 antagomir nanoparticles. (B-F) Quantitative micro-CT analysis of bone parameters: (B) Cortical bone mineral density (BMD) (C) Bone volume / Tissue volume (BV/TV) (D) Trabecular number (Tb. N) (E) Trabecular spacing (Tb. Sp) (F) Trabecular thickness (Tb. Th) Data are expressed as mean ± SD (n = 5 independent animals). One-way analysis of variance (ANOVA) and LSD test were used for statistical analysis.

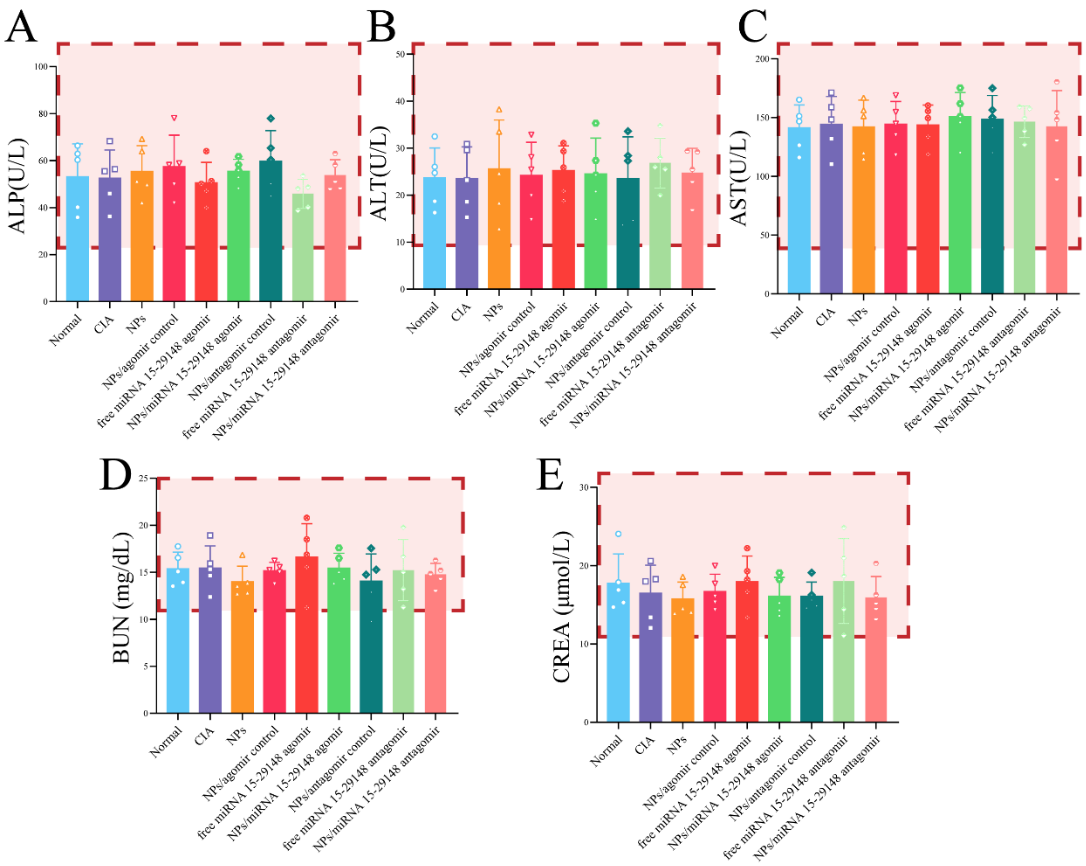

Supplemental Figure 26: In vivo systemic toxicity of nanoparticles in mice. (A) Alkaline phosphatase (ALP), (B) Alanine aminotransferase (ALT), (C) Aspartate aminotransferase (AST), (D) Blood urea nitrogen (BUN), and (E) Creatinine (CREA) levels of mice, where serum was collected after 8 weeks of administration. The dashed lines represent the average range of serum biochemical parameters in healthy mice. Data are presented as mean value ± SD (n = 5 independent animals).

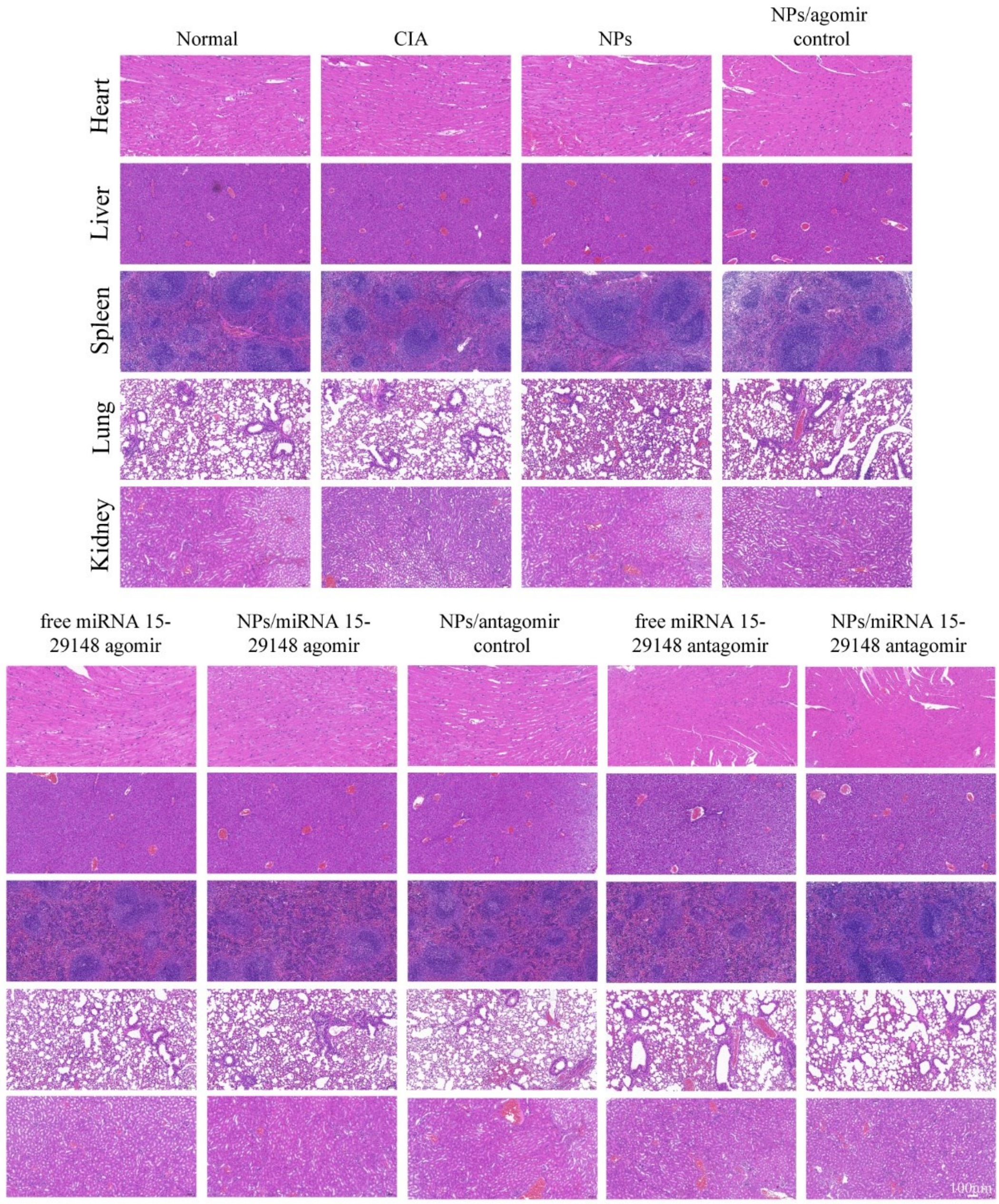

Supplemental Figure 27: Hematoxylin and eosin (H&E) staining of heart, liver, spleen, lung, and kidney of mice, where tissues were collected after 8 weeks of administration. Scale bar: 100 μm. A representative image of three biologically independent samples from each group is shown.

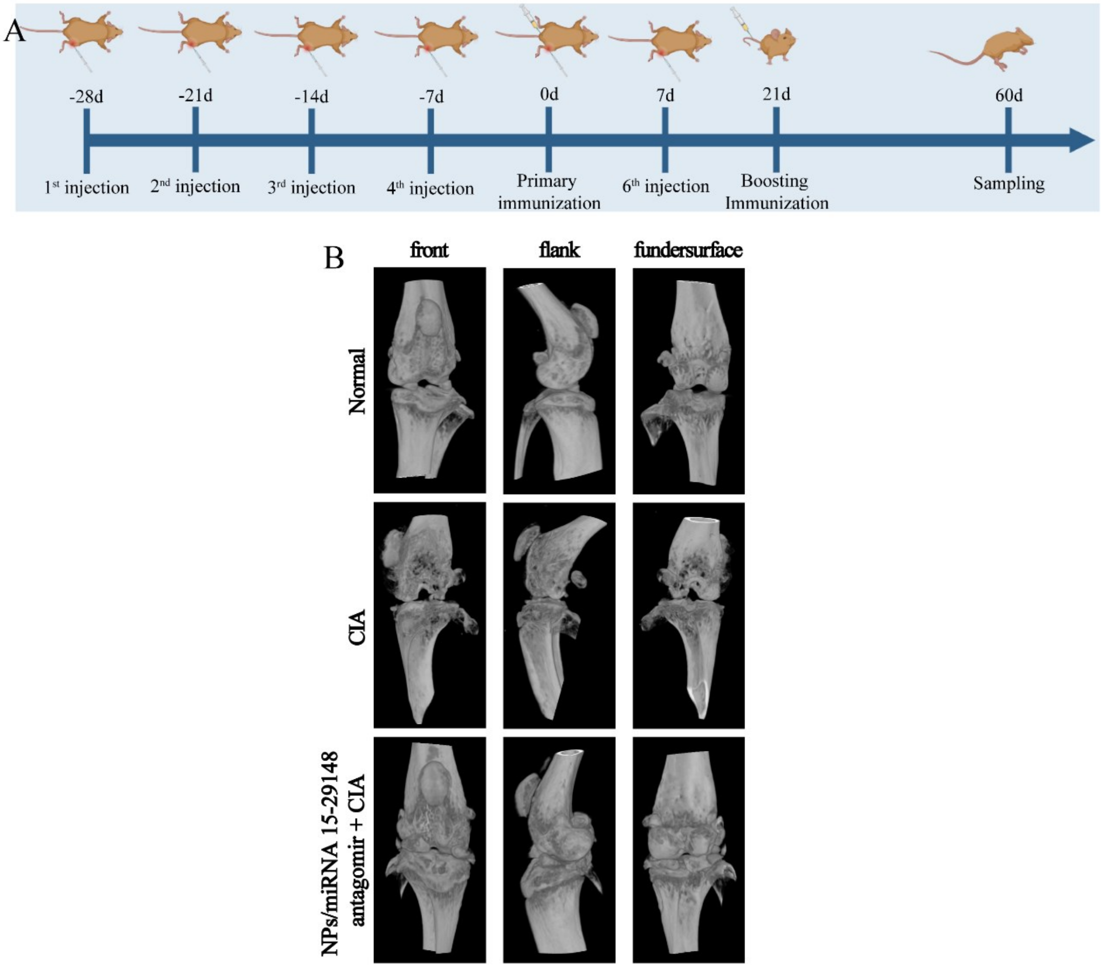

Supplemental Figure 28: (A) Summary of the experimental setup for prophylactic injection of miRNA 15-29148 antagomir or its control.

(B) Representative microCT images of the knee joint 60 days after initial immunization. Representative images of three biologically independent samples in each group are shown.

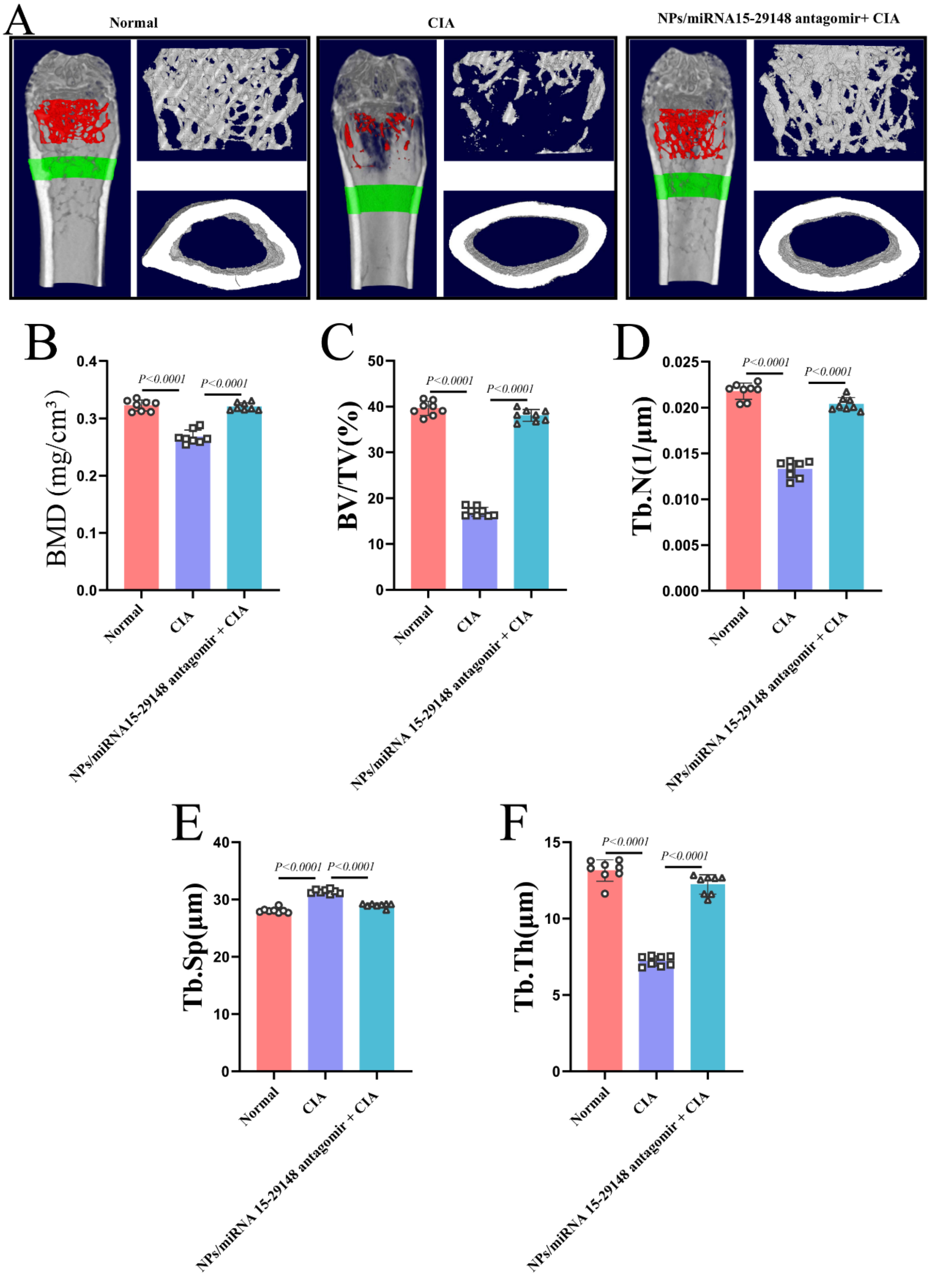

Supplemental Figure 29: (A) The representative micro-CT images of CIA mice 60 days after initial immunization. (B-F) Quantitative micro-CT analysis of: (B) Cortical bone mineral density (BMD), (C) Bone volume / Tissue volume (BV/TV), (D) Trabecular number (Tb. N), (E) Trabecular spacing (Tb. Sp), (F) Trabecular thickness (Tb. Th). Data are expressed as mean ± SD (n = 5 independent animals). One-way analysis of variance and LSD test were used for unilateral statistical analysis.

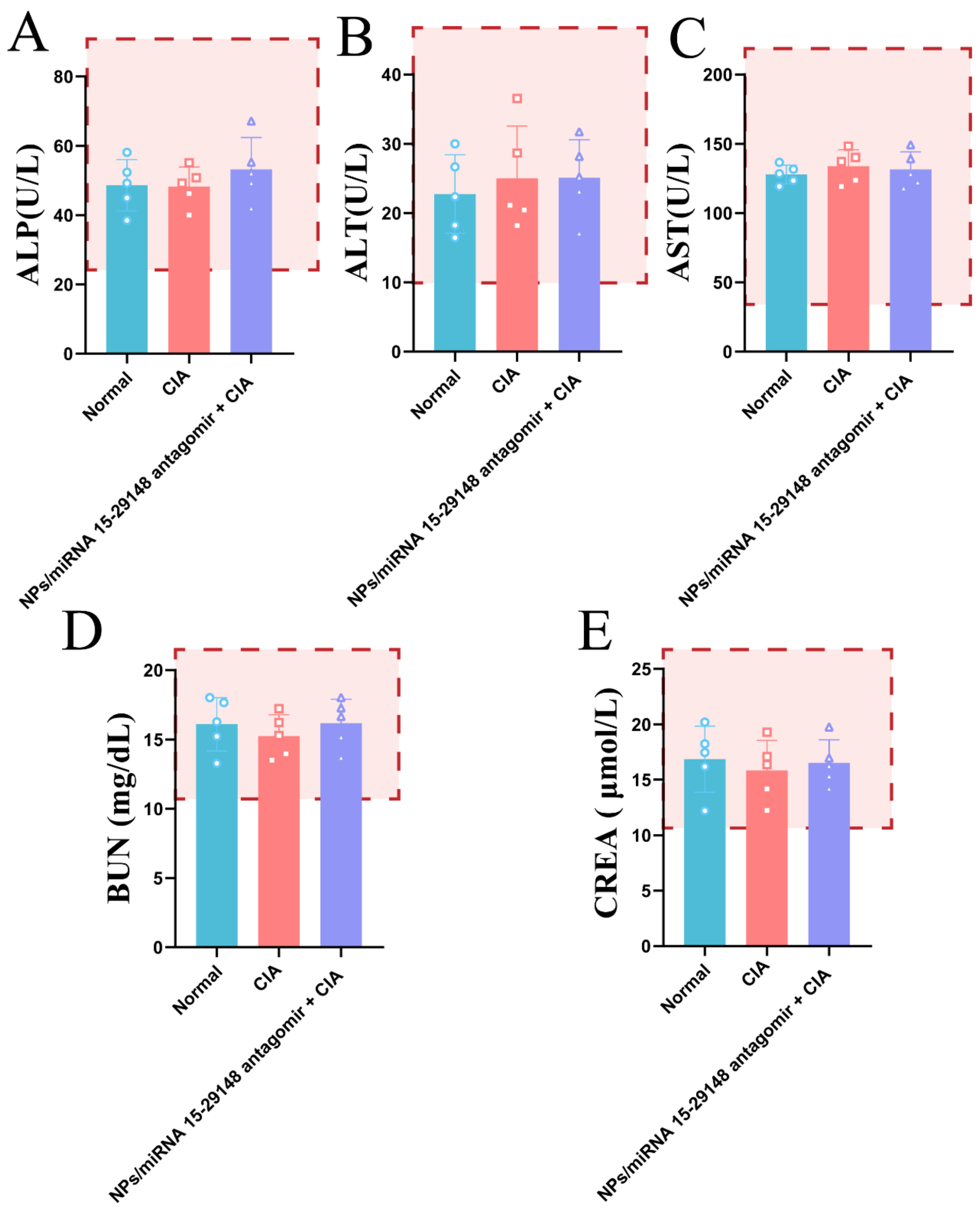

Supplemental Figure 30: (A) Alkaline phosphatase (ALP), (B) Alanine aminotransferase (ALT), (C) Aspartate aminotransferase (AST), (D) Blood urea nitrogen (BUN), and (E) Creatinine (CREA) levels of mice, in which the serum was collected after 8 weeks of administration. The dashed lines represent the average range of serum biochemical parameters in healthy mice. Data are presented as mean value ± SD (n = 5 independent animals).

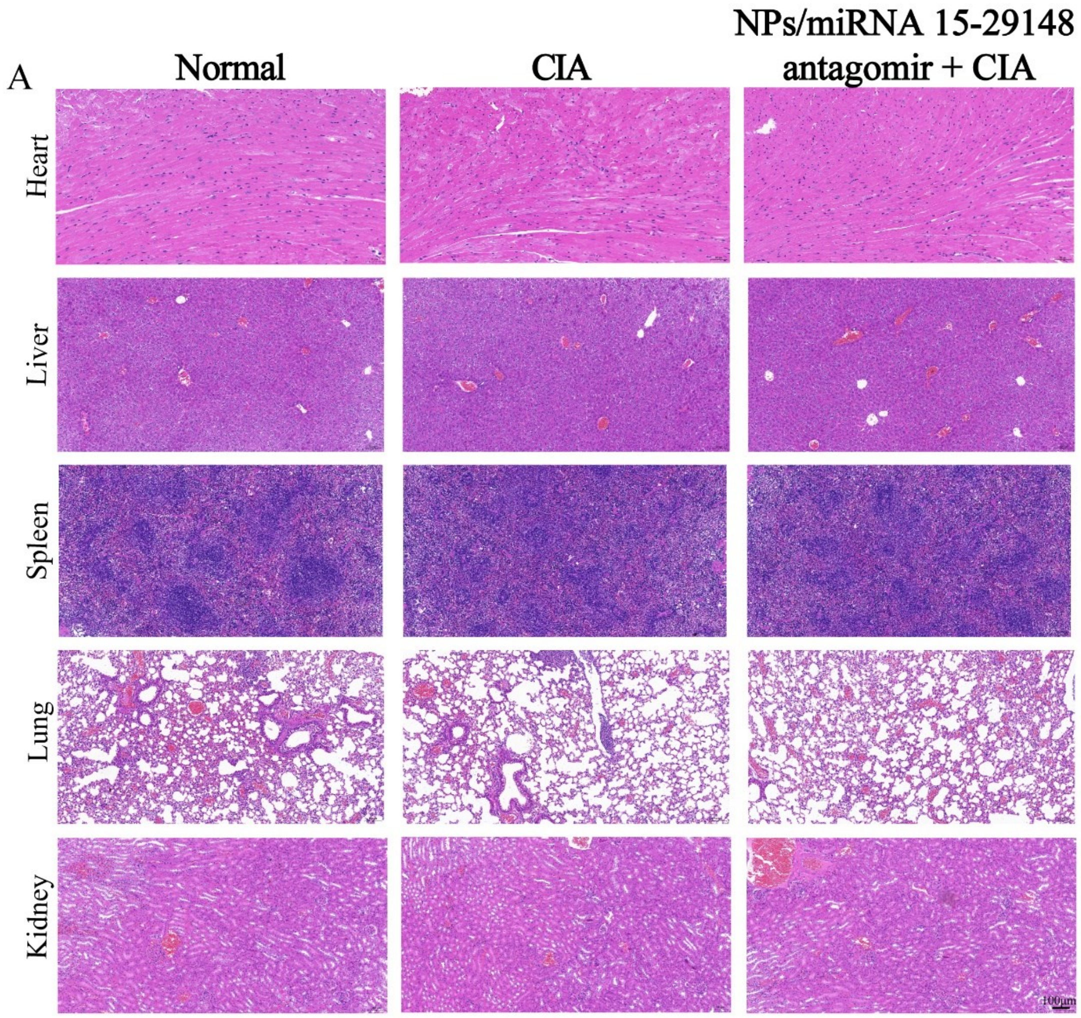

Supplemental Figure 31: (A) H&E staining of heart, liver, spleen, lung, and kidney tissues collected from mice after 8 weeks of administration. Scale bar: 100 μm. A representative image of three biologically independent samples from each group is shown.
